# Increasing risk of mortality across the spectrum of aortic stenosis is independent of comorbidity & treatment: An international, parallel cohort study of 248,464 patients

**DOI:** 10.1371/journal.pone.0268580

**Published:** 2022-07-11

**Authors:** Jordan B. Strom, David Playford, Simon Stewart, Stephanie Li, Changyu Shen, Jiaman Xu, Geoff Strange

**Affiliations:** 1 Department of Medicine, Cardiovascular Division, Beth Israel Deaconess Medical Center, Boston, Massachusetts, United States of America; 2 Richard A. and Susan F. Smith Center for Outcomes Research in Cardiology, Beth Israel Deaconess Medical Center, Boston, Massachusetts, United States of America; 3 Harvard Medical School, Boston, Massachusetts, United States of America; 4 Institute for Health Research, The University of Notre Dame, Fremantle, Western Australia, Australia; 5 School of Medicine, University of Notre Dame, Fremantle, Western Australia, Australia; 6 Center for Healthcare Delivery Sciences, Beth Israel Deaconess Medical Center, Boston, Massachusetts, United States of America; 7 Heart Research Institute, Sydney, New South Wales, Australia; 8 Department of Cardiology, Royal Prince Alfred Hospital, Sydney, New South Wales, Australia; 9 Faculty of Medicine and Health, University of Sydney, New South Wales, Australia; Charité Universitätsmedizin Berlin - Campus Virchow-Klinikum: Charite Universitatsmedizin Berlin - Campus Virchow-Klinikum, GERMANY

## Abstract

**Background:**

While large scientific and medical evidence has demonstrated the increased risk of death and cardiovascular mortality in patients with severe AS, the independent contribution of moderate AS to an increased risk of death remains uncertain.

**Methods and findings:**

We conducted a multicenter study including a cohort of 30,865 US patients and another cohort of 217,599 Australian patients with equivalent echocardiographic and aortic valve profiling over the same period (2003–2017). During a median 5.2 years (US) and 4.4 years (Australian) follow-up, the risk of death (hazard ratio) of patients with moderate AS as compared to those without AS was 1.66 (95%CI 1.52–1.80) and 1.37 (95%CI 1.34–1.41) in the US and Australian cohorts, even after adjusting this analysis for age and sex. This increased risk of death and cardiovascular mortality (odds ratio) in patients with moderate AS was consistent also across subgroups of left ventricular ejection fraction (LVEF) (subgroups of LVEF < 40%, 40–49%, 50–59%, and ≥ 60%: OR of moderate AS for CV mortality 2.0 [95%CI 1.4–2.7], 1.7 [95%CI 1.2–2.4], 1.5 [95%CI 1.1–1.9], and 1.4 [95%CI 1.2–1.6], respectively).

**Conclusions:**

The findings of this study suggest that patients with moderate AS have a potential increased risk of death and cardiovascular mortality, regardless of age, sex, and LVEF. Hence, these data suggest the need to develop specific strategies to detect and treat individuals with moderate AS.

## Introduction

Aortic stenosis (AS) is one of the most prevalent forms of valvular heart disease within the ageing populations of high-income countries [1]. Characterized by increasing obstruction to left ventricular outflow due to calcification of the aortic valve (AV) leaflets and a parallel rise in transvalvular velocities [2], it is a progressive, degenerative condition with a steep age-related incidence rate (~18 cases per 1000 person-years follow-up overall) among adult cardiac patients [3]. Historically, AS management has predominantly focused on surgical aortic valve replacement (SAVR) for those with severe symptomatic AS due to a high mortality risk without surgery [4]. The therapeutic management of such cases has been expanded with the advent of transcatheter aortic valve replacement (TAVR) [5]. However, for the remainder of AS cases (including asymptomatic severe AS and those with mild-to-moderate AS), a more conservative “watchful waiting” strategy, has been long recommended [5].

Whether this essentially dichotomised, watchful wait approach to AS management (largely based on the evidence derived from a relatively small series of single-centre historical studies) is warranted has been the subject of ongoing debate.[6] This debate has intensified with the emergence of outcome data from large, real-world, longitudinal cohort studies, suggesting that moderate AS is also associated with high mortality rates could be more proactively addressed [7]. This emerging evidence has explicitly shaped new European guidelines for the management of AS, with recommended annual follow-up of those presenting with moderate AS; ideally with a referral to a Heart Valve Clinic if indicated [8]. However, whether mild-to-moderate forms of AS independently contribute to the excess mortality seen in such patients remains unknown. Specifically, the presence of clinical comorbidities and their treatments, such as coronary artery disease (CAD) or heart failure (HF), may confound this the observed risk [6].

As part of an international collaboration, we therefore tested the hypothesis that a continuum of increasing mortality across the spectrum of AS severity would be evident in two very different and geographically diverse healthcare systems. Furthermore, we sought to determine if a similar pattern of mortality would persist after accounting for demographic, echocardiographic parameters, and a comprehensive list (>30) of clinical conditions and therapeutics that could impact an individual’s prognosis.

## Methods

### Study design

This was an international, multicentre, parallel, observational study of two large real-world patient cohorts with individual data linkage. It adheres to the REporting of studies Conducted using Observational Routinely-collected health Data (RECORD) Statement [9]. Ethical approval was provided by the Institutional Review Boards at the Beth Israel Deaconess Medical Centre (Boston, Massachusetts) in the US, and the Royal Prince Alfred Hospital (Sydney, NSW) and the University of Notre Dame (Perth, WA) in Australia with a waiver of informed consent from both Institutional Review Boards.

### Study cohorts

The two study cohorts were purposefully selected and matched (with independent analysis conducted in parallel), due to their complementary data elements (S1 Fig). A total of 631,824 patients from 23 centres across Australia with echocardiographic report data linked to all-cause mortality from the Australian National Death Index were enrolled between January 1^st^ 2003 to December 31^st^ 2017 as part of the National Echocardiographic Database of Australia (NEDA). This is a predominantly outpatient-derived cohort [10]. An equivalent, parallel cohort of 66,846 patients referred for echocardiography at the Beth Israel Deaconess Medical Centre in Boston, Massachusetts was included during the same period, with predominantly inpatient-derived echocardiograms. The US data were directly linked to 100% inpatient Medicare Fee-for-service claims, 2003–2017. Individuals with at least one documented echocardiogram and sufficient data to categorise AS severity according to contemporary guidelines [2] were included. Those aged <65 years (due to inability to link them to mortality outcomes in the US cohort) or those who had undergone aortic valve replacement (AVR) were excluded. The data supporting this study are not available due to prior data use agreements with the Centers for Medicare and Medicaid Services (US) and NEDA partner institutions.

### Aortic stenosis classification

All individuals were categorised based on standard echocardiographic criteria [2, 11] as having–

- No AS (mean AV gradient <10.0 mmHg or peak AV velocity <2.0 m/s),
- Mild AS (10.0–19.9 mmHg / 2.0–2.9 m/s and AV area [AVA] ≥1.0 cm^2^),
- Moderate AS (20.0–39.9 mmHg / 3.0–3.9 m/s and AVA ≥1.0 cm^2^), or
- Severe AS (≥40.0 mmHg / ≥4.0 m/s or AVA <1.0 cm^2^), using the last documented echocardiogram (for those with multiple echocardiograms) to define AS severity.

Also consistent with contemporary guidelines [2, 11], an AVA of <1.0 cm^2^ was used to further reclassify those with severe, low-gradient AS (AVA <1.0 cm^2^ and mean AV gradient <40.0 mmHg or peak AV velocity <4.0 m/s) and severe, high-gradient AS (≥40.0 mmHg or ≥4.0 m/s).

### Study variables

In both cohorts, age, sex, anthropometric profile, vital signs at the time of investigation and a standard list of left and right heart parameters routinely assessed by echocardiography were extracted from the reports (S1 Table). In those individuals (8,714 US and 56,026 Australian patients) with multiple qualifying echocardiograms, the known time an individual spent in each AS stage was quantified.

For the US cohort only, comprehensive additional data on the clinical profile and treatment strategies (including comorbidities such as hypertension, diabetes, CAD, the syndrome HF, chronic kidney disease, cancer, and dementia, findings from laboratory investigations, and cardiovascular and non-cardiovascular interventional strategies and medications) were derived from linkage to institutional datasets and Medicare Chronic Comorbidity Warehouse indicators (S1 Table). The latter represents indicators for comorbidity status based on validated algorithms using inpatient and outpatient Medicare claims for the 1–2 years preceding the index echocardiogram.

### Study outcomes

The primary outcome was all-cause death, determined via linkage to the National Death Index administered by the Australian Institute of Health and Welfare [12] or vital status in the US Medicare Beneficiary Summary File [13]. While all included patients received echocardiograms between January 1^st^ 2003 and December 31^st^ 2017, vital status information was available for a minimum of 12 months (up to December 31^st^ 2018 in the Australian cohort and December 31^st^ 2017 in the US cohort). Individuals alive on these dates were censored.

### Statistical analyses

Parallel analyses applying the same statistical methods were performed separately for both cohorts (S2–S5 Tables). The baseline characteristics of participants at the time of their index echocardiogram are described according to AS category using means and standard deviations or medians and interquartile ranges (IQR) for continuous variables and numbers and proportions for categorical variables. Actual mortality rates were determined at 1- and 5-year in 26,673 and 15,880 US, and 214,140 and 104,613 Australian patients, respectively, with complete follow-up at these timepoints. The Kaplan-Meier method was used to estimate all-cause mortality within 10-year of the index echocardiogram. Cox Proportional Hazards models were used to generate hazard ratios (HR) and 95% confidence intervals (CI) for all-cause mortality across AS severity categories. A series of sensitivity analyses were also conducted to address potential confounding and important clinical subgroups. No imputation was performed. Based on 14,481 and 89,054 all-cause deaths in US and Australian cohorts respectively, all relevant covariates could be included in Cox models without significant model overfitting. The US and Australian analyses were performed by JS, JX, and CS, and SS and GS and conducted in SAS v 9.4 (SAS Institute, Cary NC) and SPSS v26.0 (IBM Corp, Armonk NY), respectively. A two-tailed p-value <0.01 for statistical significance was applied to account for multiple hypothesis testing.

## Results

### Study cohorts

**Fig 1:**
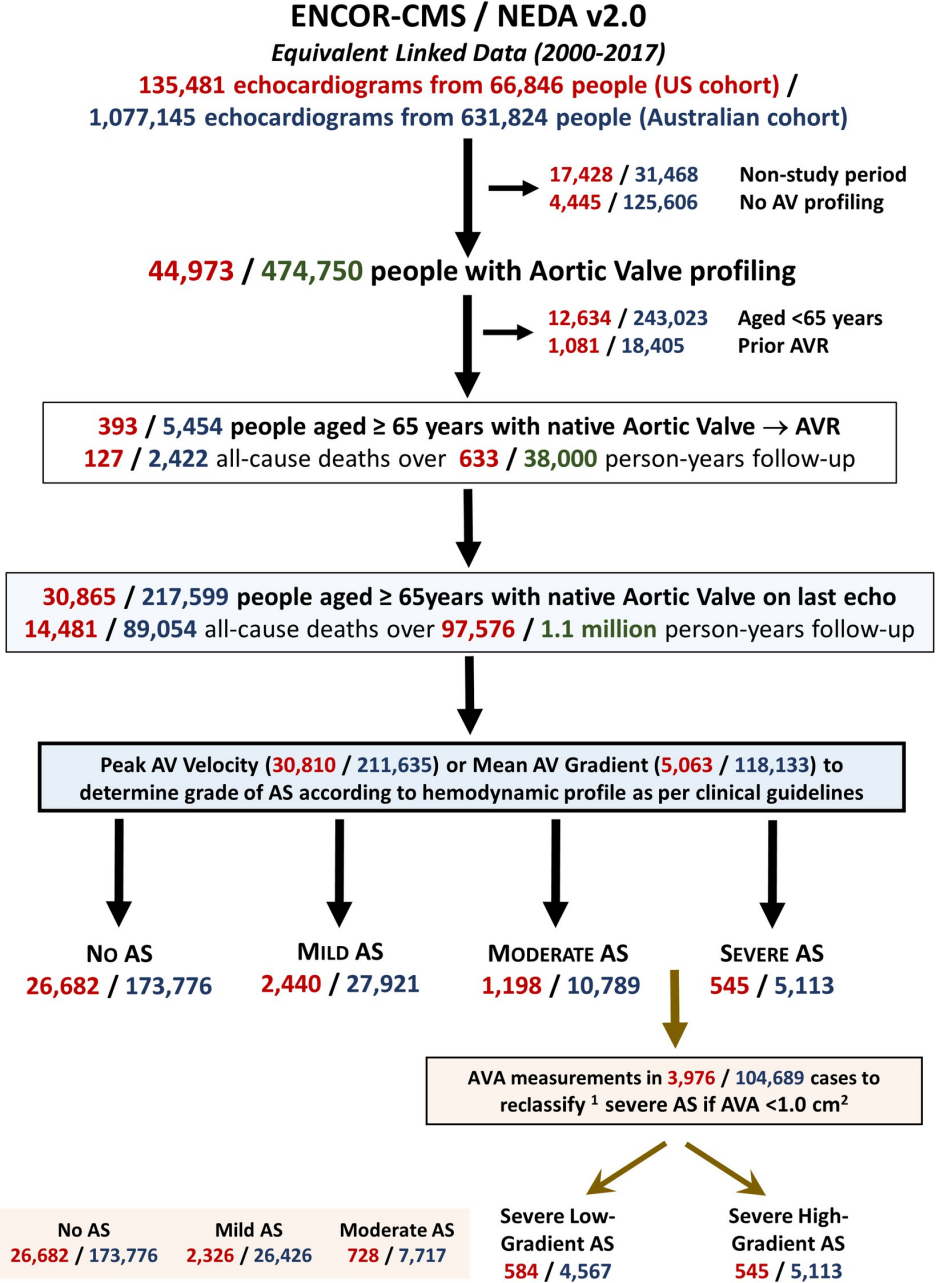
Study flowchart. This flowchart shows the parallel points of inclusion and exclusion across the two study cohort–including 1,081 (US) / 184,405 (Australia) cases with evidence of prior aortic valve replacement excluded from study analyses.

A total of 30,865 US (mean age 77.4 years, 52.2% women) and 217,599 Australian (mean age 76.0 years, 49.3% women) individuals aged ≥65 years with AV profiling were identified (Fig 1). Baseline characteristics according to AS stage are provided in Table 1 (US cohort) and S6 Table (Australian cohort). In the US cohort, a total of 26,682 (86.5%), 2,440 (7.8%), 1,198 (3.9%) and 545 (1.8%) individuals were categorised as no, mild, moderate, and severe AS, respectively. In the Australian cohort, an equivalent total of 173,776 (79.9%), 27,921 (12.8%), 10,789 (5.0%) and 5,113 (2.3%) individuals were categorised as no, mild, moderate, and severe AS, respectively.

**Table 1:** Baseline characteristics (US cohort).

	Total (N = 30,865)	No AS (N = 26,682)	Mild AS (N = 2440)	Moderate AS (N = 1198)	Severe AS (N = 545)
**Demographic profile**					
Age, years	77.4 ± 8.3	76.8 ± 8.1	80.9 ± 8.3	81.8 ± 8.2	82.7 ± 8.5
Women, %	16,116 (52.2)	13,973 (52.4)	1267 (51.9)	563 (47.0)	313 (57.4)
White, %	25,339 (82.5)	21,624 (81.4)	2119 (87.5)	1100 (92.0)	496 (91.5)
Black, %	2800 (9.1)	2577 (9.7)	146 (6.0)	58 (4.8)	19 (3.5)
**Clinical Profile**					
Inpatient, %	18,401 (59.6)	15,595 (58.4)	1597 (65.5)	827 (69.0)	382 (70.1)
BMI, kg/m^2^	27.5 ± 6.1	27.5 ± 6.1	27.9 ± 6.1	27.3 ± 6.0	26.8 ± 5.9
Systolic BP, mmHg	131 ± 21.8	131 ± 21.6	132 ± 22.7	129 ± 23.2	126 ± 20.8
Diastolic BP, mmHg	69.4 ± 13.4	69.8 ± 13.2	67.5 ± 14.4	65.3 ± 13.6	65.8 ± 13.7
Heart rate, bpm	73.8 ± 15.9	73.8 ± 15.8	74.3 ± 16.4	73.5 ± 15.7	73.7 ± 15.4
eGFR, L/min/1.73m	76.6 (0–262)	81.7 (0–269)	56.9 (0–219)	0 (0–192)	102 (0–227)
NT-proBNP, pg/ml	2722 (803–8105)	2479 (712–7488)	3651 (1179–10927)	5541 (1658–12730)	5601 (2391–17495)
**Past Medical History**					
Diabetes Mellitus, %	9253 (30.0)	7819 (29.3)	828 (33.9)	441 (36.8)	165 (30.3)
Hypertension, %	19,858 (64.3)	16,779 (62.9)	1764 (72.3)	902 (75.3)	413 (75.8)
IHD, % / Revascularized, %	15,055 (48.8) / 2158 (14.3)	12,496 (46.8) / 1854 (14.8)	1378 (56.5) / 167 (12.1)	812 (67.8) / 102 (12.6)	369 (67.7) / 35 (9.5)
HF, %	12,698 (41.1)	10,340 (38.8)	1263 (51.8)	756 (63.1)	339 (62.2)
**Aortic Valve Profile**					
Peak velocity, m/s	1.7 ± 0.7	1.5 ± 0.3	2.5 ± 0.3	3.5± 0.3	4.7 ± 0.5
Mean gradient, mmHg	20.6 ± 14.8	8.1 ± 6.0	13.9 ± 3.9	28.3 ± 5.9	53.6 ± 12.2
Valve area, cm^2^	1.4 ± 0.6	2.1 ± 0.7	1.4 ± 0.4	1.0 ± 0.3	0.7 ± 0.2
AR, %	276 (2.5)	184 (2.0)	26 (3.4)	37 (7.2)	19 (9.4)
**Right Ventricular Function & Dimensions**					
Peak TR velocity, m/s	2.8 ± 0.5	2.7 ± 0.5	2.9 ± 0.5	3.0 ± 0.5	3.1 ± 0.6
Moderate or greater TR, %	1727 (17.1)	1395 (16.2)	186 (20.8)	107 (25.6)	39 (23.2)
**Left Ventricular Function & Dimensions**					
LAVI, mL/m^2^	30.8 ± 11.4	30.2 ± 11.2	33.4 ± 11.5	35.4 ± 12.3	41.5 ± 13.6
LVEDD, cm	4.5 ± 0.8	4.5 ± 0.8	4.5 ± 0.8	4.5 ± 0.8	4.4 ± 0.7
LVESD, cm	2.8 ± 0.8	2.8 ± 0.8	2.8 ± 0.8	2.9 ± 0.8	2.8 ± 0.8
LVEF, %	61.7 ± 16.6	61.8 ± 16.5	61.4 ± 17.2	60.6 ± 18.3	63.2 ± 16.6
Transmitral E/e’ ratio	12.2 ± 5.5	11.8 ± 5.1	14.0 ± 6.3	15.8 ± 7.7	17.0 ± 6.8
Transmitral E/A ratio	1.1 ± 0.7	1.1 ± 0.7	1.1 ± 0.7	1.2 ± 0.7	1.1 ± 0.7
SVI, mL/m^2^	39.1 ± 11.9	38.7 ± 11.6	41.9 ± 13.2	41.1 ± 11.9	40.3 ± 13.9
Moderate or greater MR, %	1564 (15.0)	1203 (13.6)	171 (18.6)	137 (30.5)	53 (29.0)
**Pharmacotherapy**					
Anticoagulant, %	3990 (12.9)	3416 (12.8)	336 (13.8)	170 (14.2)	68 (12.5)
Diuretic, %	9100 (29.5)	7692 (28.8)	833 (34.1)	402 (33.6)	173 (31.7)
Neurohormonal antagonist, %	9908 (32.1)	8554 (32.1)	833 (34.1)	374 (31.2)	147 (27.0)
Antiplatelet, %	11,269 (36.5)	9719 (36.4)	903 (37.0)	465 (38.8)	182 (33.4)
Anti-arrhythmic, %	12,018 (38.9)	10,345 (38.8)	998 (40.9)	473 (39.5)	202 (37.1)
Beta-blocker, %	12,615 (40.9)	10,938 (41.0)	1002 (41.1)	475 (39.6)	200 (36.7)

Data are presented as the mean ± SD, n (%) or median (95% CI’s). Body mass index, BMI; blood pressure (BP); estimated glomerular filtration rate, eGFR; ischemic heart disease, IHD; heart failure, HF; aortic regurgitation, AR; tricuspid regurgitation, TR; left atrial volume index, LAVI; left ventricular end-diastolic dimension, LVEDD; left ventricular end-systolic dimension, LVESD; left ventricular ejection fraction, LVEF; stroke volume index, SVI; mitral regurgitation, MR.

### Overall pattern of mortality

During a median (IQR) follow-up of 5.2 (2.1–9.1) years, there were 14,481 deaths (46.9%) in the US cohort. These comprised 11,815 (81.6%) deaths among those with no AS and 1,453 (10.0%), 821 (5.7%), and 392 (2.7%) in those with mild, moderate, and severe AS, respectively (Table 2). Total mortality was 44.3% in those without AS, 59.5% in those with mild AS, 68.5% in those with moderate AS, and 71.9% in those with severe AS (univariate HRs in S7 Table).

**Table 2:** All-cause mortality outcomes according to aortic stenosis severity.

	Actual 1-year Mortality (%)	Actual 5-year Mortality (%)	Died within 10 years (%)	Median Time to Death (years)	Age- & Sex-adjusted HRs (95% CI)
**All-cause deaths, %**					
US cohort (N = 30,865)	7,430 (24.1)	12,386 (40.1)	14,245 (46.2)	5.2 (2.1 to 9.1)	-
Australian cohort (N = 217,599)	22,171 (10.4)	46,137 (44.1)	83,593 (38.4)	2.7 (0.90 to 5.1)	-
**No AS, %**					
US cohort (N = 26,682)	5,904 (22.1)	9,975 (37.4)	11,606 (43.5)	6.0 (1.2–13.5)	***Reference Groups***
Australian cohort (N = 173,776)	15,886 (9.3)	32,648 (29.8)	60,641 (34.9)	2.7 (0.9 to 5.2)	
**Mild AS, %**					
US cohort (N = 2,449)	781 (32.0)	1,288 (52.8)	1,434 (58.8)	2.7 (0.4–6.9)	**1.37 (1.28 to 1.47)**
Australian cohort (N = 27,921)	3,566 (13.0)	7,661 (40.0)	13,439 (48.1)	2.7 (0.9 to 5.0)	**1.19 (1.17 to 1.21)**
**Moderate AS, %**					
US cohort (N = 1,198)	515 (43.0)	762 (63.6)	819 (68.4)	1.3 (0.2–4.9)	**1.66 (1.52 to 1.80)**
Australian cohort (N = 10,789)	1,706 (16.0)	3,675 (47.0)	6,179 (57.3)	2.4 (0.9 to 4.8)	**1.37 (1.34 to 1.41)**
**Severe AS, %**					
US cohort (N = 545)	233 (42.8)	363 (66.7)	388 (71.2)	1.4 (0.2–5.7)	**1.67 (1.48 to 1.88)**
Australian cohort (N = 5,113)	1,013 (20.0)	2,153 (54.5)	3,334 (65.2)	2.2 (0.7 to 4.6)	**1.53 (1.48 to 1.58)**

US Cohort–Actual 1-year and 5-year mortality were calculable in 26,673 (86.3%) and 15,880 (51.4%) of cases. The age and sex-adjusted model of all-cause mortality included 14,481 deaths and 16,384 censored cases during median (interquartile range) of 5.2 (2.1 to 9.1) years follow-up. Female sex (hazard ratio 0.80, 95% CI 0.77–0.83) and age per year (hazard ratio 1.06, 95% CI 1.06–1.06); P< 0.001 for all comparisons. Australian cohort–Actual 1-year and 5-year mortality were calculable in 214,140 (98.4%) and 140,613 (64.6%) of cases. The age and sex-adjusted model of all-cause mortality included 89,054 deaths and 128,545 censored cases during median (interquartile range) 4.4 (2.3 to 7.2 years) follow-up. Female sex (hazard ratio 0.72 (95% CI, 0.71–0.73) and age per year (hazard ratio 1.09, 1.09–1.09); P <0.001 for all comparisons.

During a median (IQR) follow-up of 4.4 (2.3–7.2) years, there were 89,054 deaths in the Australian cohort. These comprised 64,969 (73.0%) deaths among those with no AS, and 14,121 (15.9%), and 6,481 (7.3%), and 3,483 (3.9%) among those with mild, moderate, and severe AS, respectively. Total mortality was 37.4% in those with without AS, 50.6% in those with mild AS, 60.1% in those with moderate AS, and 68.1% in those with severe AS.

### Comparison of mortality in the US and Australian cohorts

**Fig 2:**
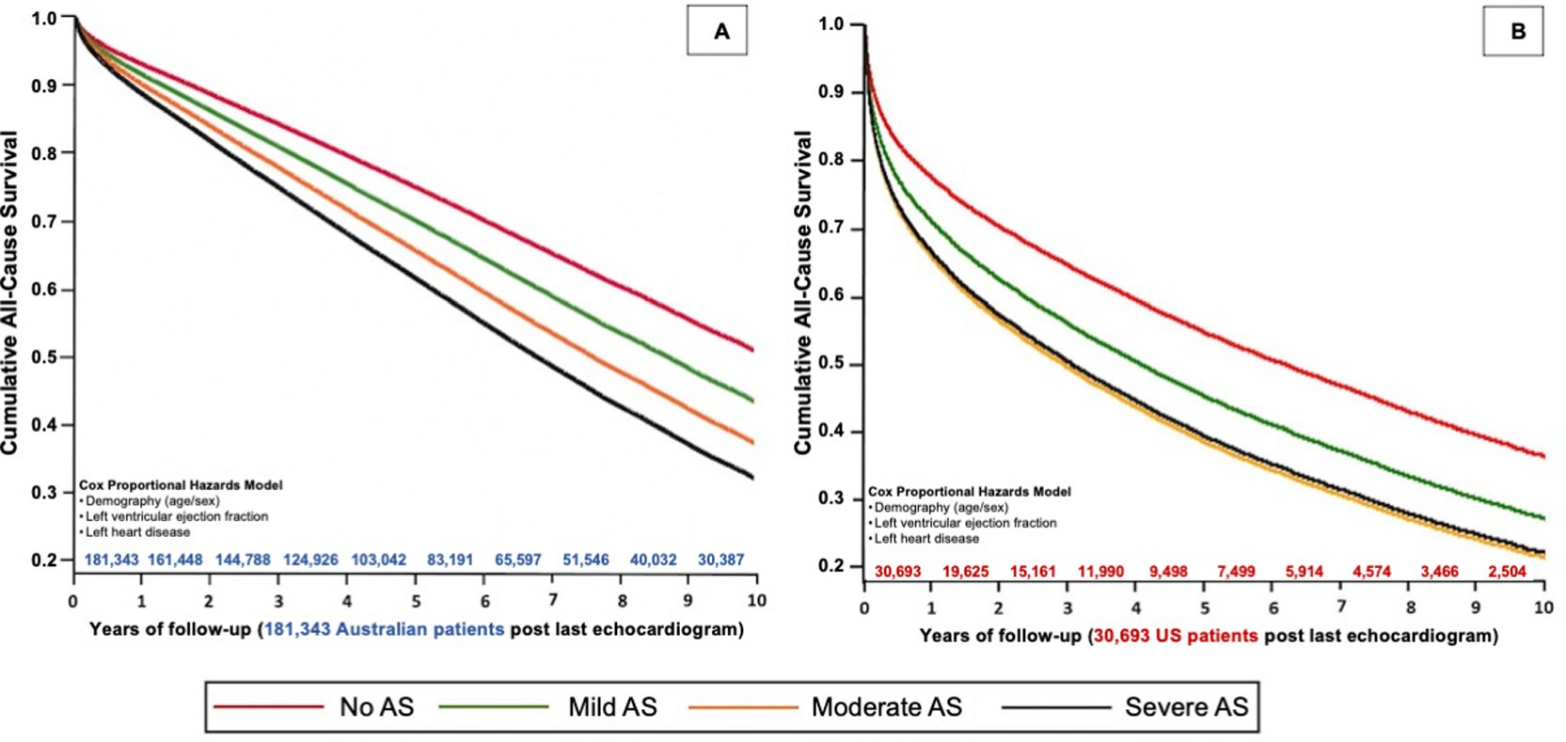
Adjusted all-cause mortality according to AS stage. These adjusted Kaplan-Meier curves evaluate the risk of all-cause mortality over 10-years from the last echocardiogram according to AS severity in Australian (2A; left) and US (2B; right) cohorts when adjusting for age, sex, left ventricular ejection fraction, and presence of left heart disease.

S8 Table (Australian cohort) and S9 Table (US cohort) provide univariate comparisons of baseline characteristics by 10-year mortality. Adjusting for age, sex, left ventricular ejection fraction, and evidence of left heart disease, the HR for all-cause mortality in the Australian cohort, compared to those with no AS, was 1.24 (95% CI 1.21–1.26) for mild AS, 1.47 (95% CI 1.43–1.51) for moderate AS, and 1.67 (95% CI 1.61–1.73) for severe AS (Fig 2A). In an equivalent model, the HR for all-cause mortality in the US cohort, compared to those with no AS, was 1.35 (95% CI 1.28–1.43) for mild AS, 1.68 (95% CI 1.57–1.81) for moderate AS, and 1.63 (95% CI 1.47–1.80) for severe AS (Fig 2B).

**Fig 3:**
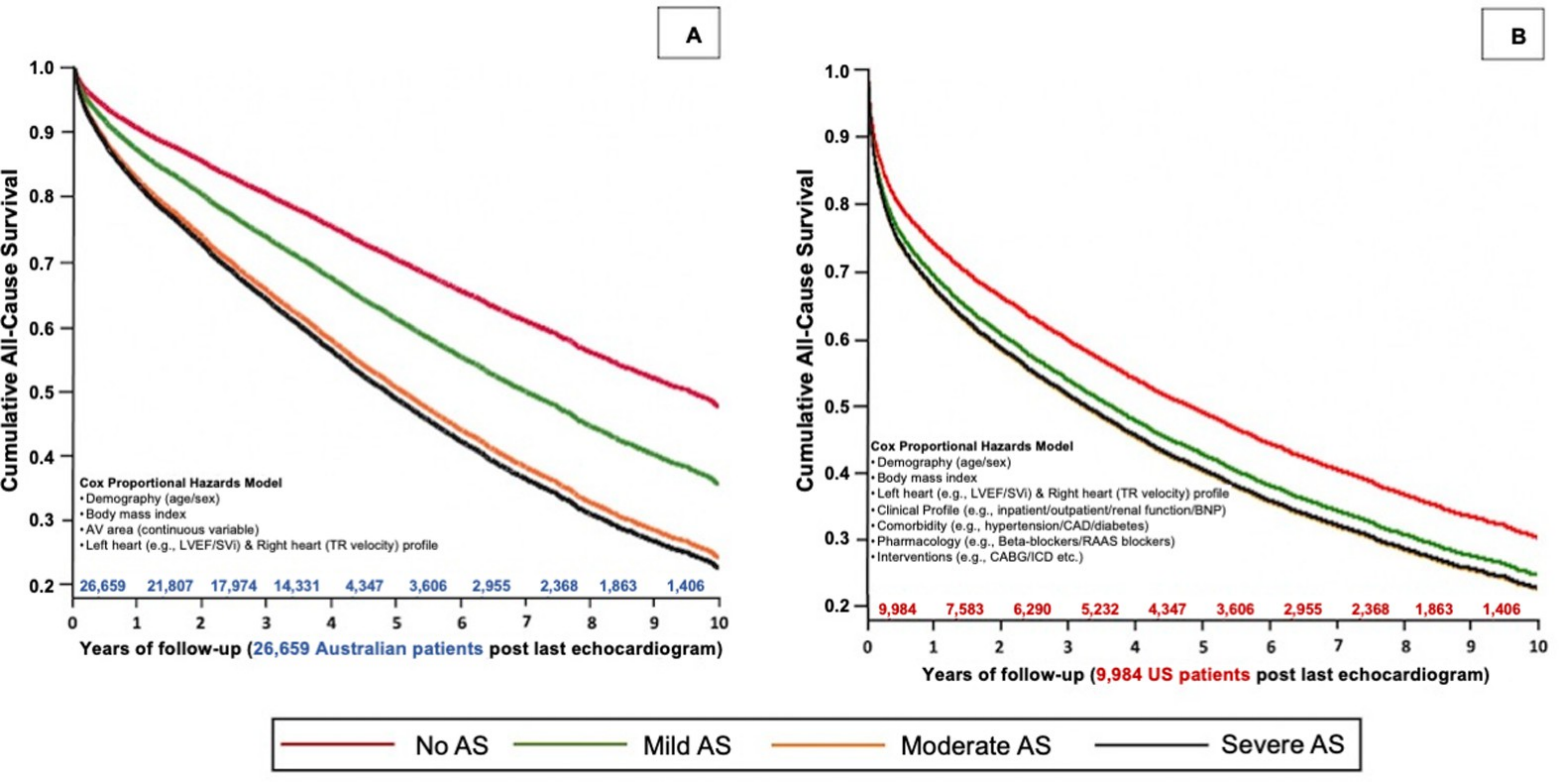
Fully adjusted all-cause mortality according to AS stage. These adjusted Kaplan-Meier curves evaluate the risk of all-cause mortality over 10-years from the last echocardiogram according to AS severity in Australian (A) and US (B) cohorts when fully adjusting for all confounders (based on available data for each cohort.

After additional adjustment for AVA as a continuous variable in the Australian cohort (S10 Table), those with moderate (HR 1.22, 95% CI 1.16–1.27) and severe AS (HR 1.35, 95% CI 1.29–1.44) continued to have similar long-term risk of mortality. In the fully adjusted Australian model (Fig 3A and S11 Table), compared to those with no AS, the HR for all-cause mortality was 1.36 (95% CI, 1.14–1.61) for mild AS, 2.06 (95% CI, 1.80–2.32) for moderate AS, and 2.03 (95% CI, 1.78–2.32) for severe AS. The equivalent fully adjusted survival curves for the US cohort (see section below) are shown in Fig 3B.

### Adjustment for clinical confounders and analysis of cardiovascular-related death

**Fig 4:**
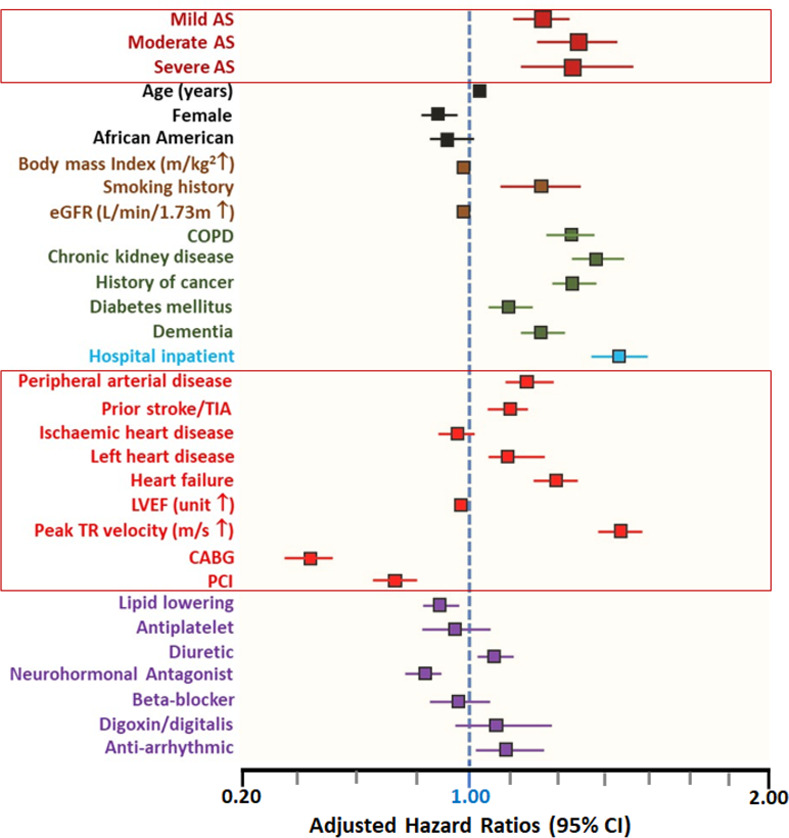
Risk of all-cause mortality according to clinical factors. The forest plot denotes the risk of all-cause mortality occurring within 10 years across the full spectrum of AS when further adjusting for a comprehensive list of clinical factors.

Fig 4 presents the risk of all-cause mortality occurring within 10 years across the full spectrum of AS when further adjusting for a comprehensive list of clinical factors. This included a history of hypertension, diabetes, ischemic heart disease, coronary revascularisation, chronic kidney disease, cancer, dementia and >30 other clinical, laboratory, and medication parameters. On this basis, compared to those with no AS, the HR for all-cause mortality was 1.26 (95% CI, 1.16–1.36) for mild AS, 1.38 (95% CI, 1.24–1.53) for moderate AS and 1.36 (95% CI, 1.17–1.59) for severe AS (all p<0.001) (S12 Table).

Additionally, as individuals with AS may be at elevated risk of non-cardiovascular death, Table 3 details the risk of cardiovascular-related death in the Australian cohort, after adjustment for age, sex, presence of left heart disease, and left ventricular ejection fraction. After full adjustment for age, sex, body mass index, presence of left heart disease, left ventricular ejection fraction, stroke volume index, time in stage, aortic valve area as a continuous measure, and AS severity, the adjusted hazard ratios, compared to no AS, were 1.42 (95% CI 1.09–1.44) for mild AS, 2.47 (95% CI 2.01–3.00) for moderate AS, and 2.92 (95% CI 2.40–3.56) for severe AS.

**Table 3:** Results of model 8: Sensitivity analysis reporting results for the relationship of as severity and cardiovascular-related death in the Australian cohort.

Australian Cohort 23,995 CV-related deaths / 181,343 patients	
**Covariates**	**Adjusted Hazard Ratios (95% CI) for All-Cause Mortality**
**Age (per 1-year increase)**	**1.11** (1.10 to 1.11)
**Female**	**0.88** (0.86 to 0.91)
**Left heart disease**	**1.35** (1.31 to 1.39)
**Left ventricular ejection fraction (per 1-% increase)**	**0.97** (0.97 to 0.97)
***Aortic Stenosis stage/severity***	
**No AS**	***Reference Group***
**Mild AS**	**1.33** (1.28 to 1.37)
**Moderate AS**	**1.88** (1.79 to 1.97)
**Severe AS**	**2.63** (2.50 to 2.78)

Displayed are the results of model 8, results of a sensitivity analysis evaluating the relationship between AS stage and cardiovascular specific mortality. This model is adjusted for age, sex, presence of left heart disease, left ventricular ejection fraction, and AS severity. A total of 181,343 individuals had complete profiling with 23,995 cardiovascular related deaths and 157,348 censored individuals. Directly comparing severe to moderate AS with regard to risk of CV-related death, the adjusted hazard ratio was 1.41 (95% CI 1.32 to 1.51). Applying the Fine and Gray method to account for the competing risk of non-CV-related mortality, there were no significant changes in the results observed. All comparisons are significant at a p < 0.001 level. CV = cardiovascular.

### Sensitivity analyses

**Fig 5:**
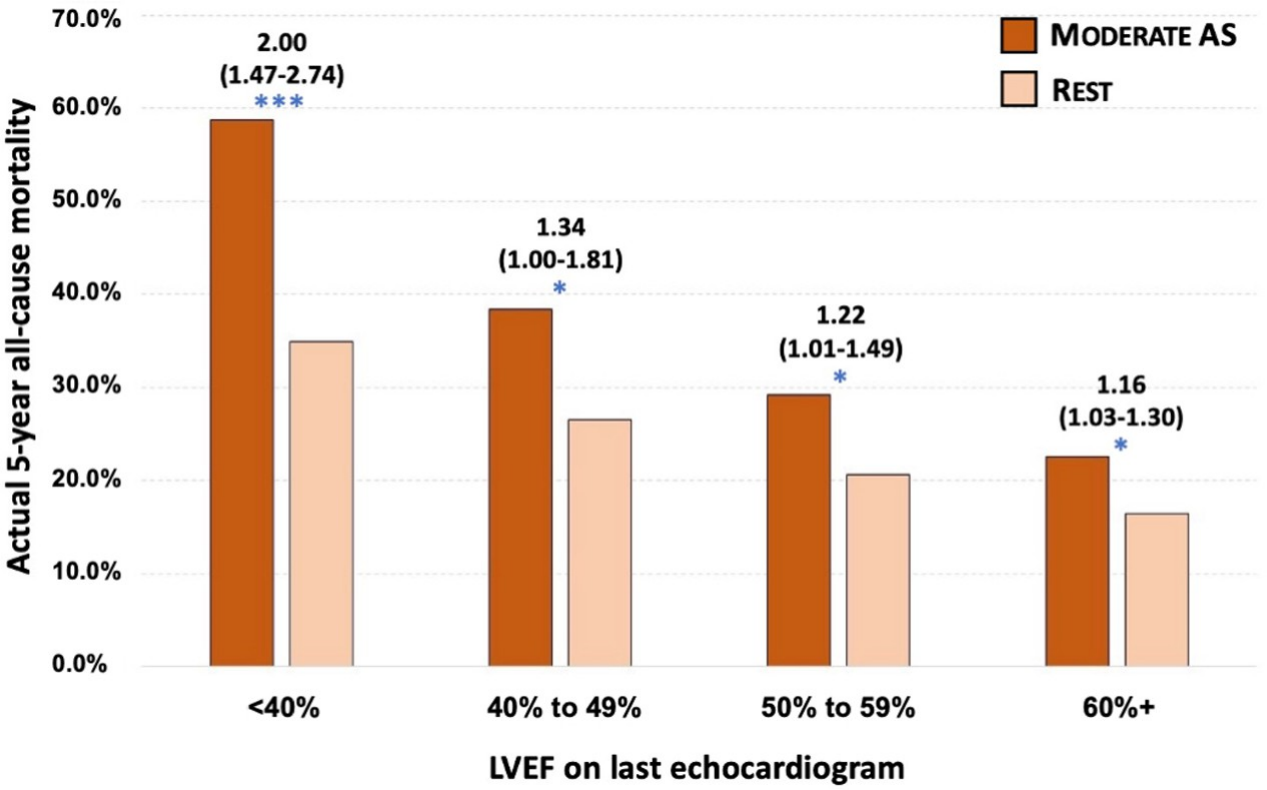
Actual 5-year rates and odds ratios for all-cause mortality by AS stage stratified by left ventricular ejection fraction category. This graph compares actual 5-year mortality (all-cause) associated with moderate AS versus the rest of the cohort when excluding all severe AS cases, according to LVEF quantified at the same time-point (last echocardiogram). Age and sex-adjusted odd ratios (95% CI) for mortality per group are shown above the bars (moderate AS group versus rest with *p<0.05, ** p<0.01 and ***p<0.001 for that group comparison) derived from multiple logistic regression. Actual 5-year mortality rates across the four LVEF subgroups (moderate AS versus rest) were—110/187 (58.8%) vs. 1427/4083 (34.9%), 81/211 (38.4%) vs. 851/3208 (26.5%), 171/585 (29.2%) vs. 1667/8038 (20.7%) and 452/1998 (22.6%) vs. 3230/19,599 (16.5%).

**Fig 6:**
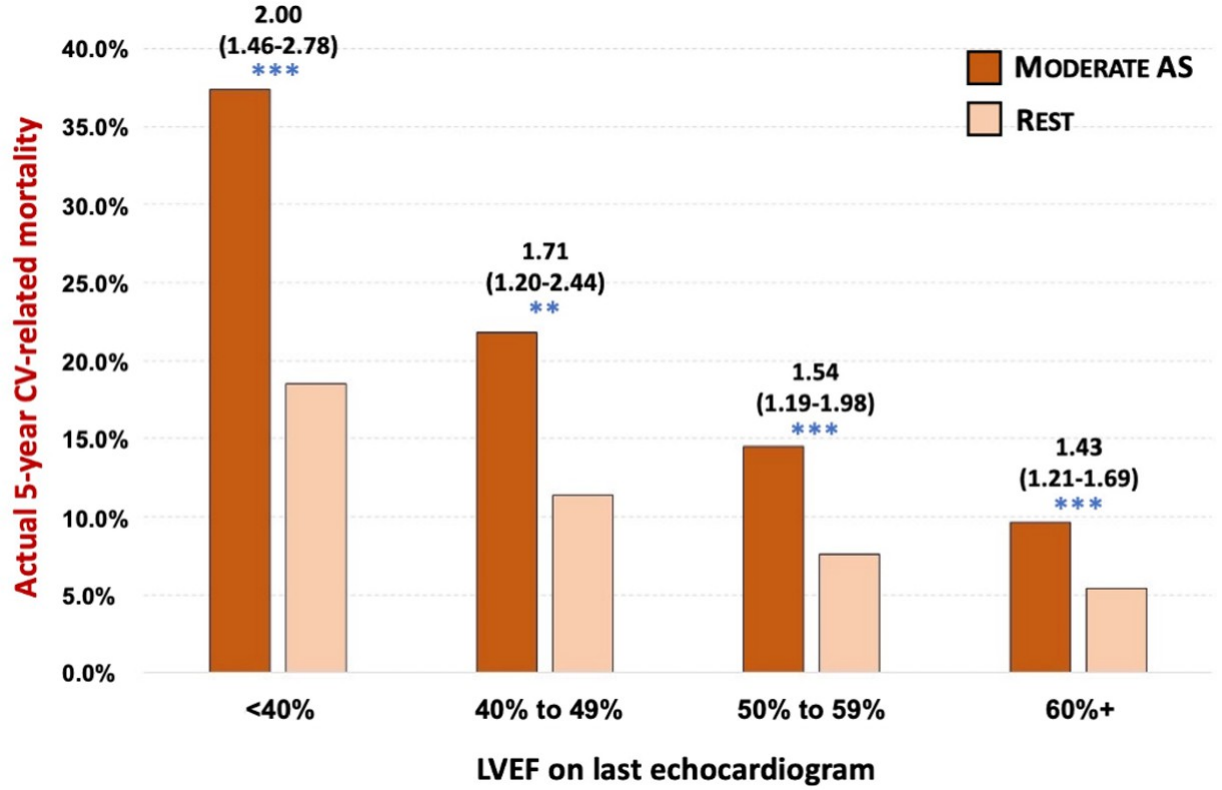
Actual 5-year rates and odds ratios for cardiovascular-related mortality by AS stage stratified by left ventricular ejection fraction category. This graph compares actual 5-year mortality (cardiovascular-related) associated with moderate AS versus the rest of the cohort when excluding all severe AS cases, according to LVEF quantified at the same time-point (last echocardiogram). Age and sex-adjusted odd ratios (95% CI) for mortality per group are shown above the bars (moderate AS group versus rest with *p<0.05, ** p<0.01 and ***p<0.001 for that group comparison) derived from multiple logistic regression. Actual 5-year cardiovascular-related mortality rates across the four LVEF subgroups (moderate AS versus rest) were—70/187 (21%) vs. 755/4083 (16%), 46/211 (21.8%) vs. 366/3208 (11.4%), 85/585 (14.5%) vs. 611/8038 (7.6%) and 191/1998 (9.6%) vs. 1049/19,599 (15.4%).

A consistent pattern of increased mortality risk amongst individuals with moderate AS was found when examining outcomes according to a) an AVA-based classification of AS, b) those aged <65 years, c) use of the first documented echocardiogram to categorise AS, d) adjustment for known time in each AS stage, e) stratified by ischemic heart disease or heart failure history, and stratified by LVEF subgroup (Figs 5 and 6 and S13–S19 Tables and S2–S4 Figs). When compared directly in fully adjusted models, individuals with moderate AS demonstrated a similar risk of mortality as those with severe AS in both the US (adjusted HR, 1.06, 95% CI 0.87–1.28) and Australian (adjusted HR, 0.99, 95% CI 0.85–1.15) patients.

## Discussion

In this unique multicentre, international, parallel-group, observational study, from two geographically diverse, real-world patient cohorts, there was a continuum of increased risk of all-cause mortality with increasing severity of AS. On a fully adjusted basis, the associated risk of mortality with moderate AS was like that of severe AS. This relationship persisted when accounting for potential confounders including the effective valve orifice areas, history of hypertension, diabetes, CAD and/or subsequent coronary revascularisation, chronic kidney disease, the syndrome HF and more than 30 other clinical, laboratory, and medication variables that would influence an individual’s clinical trajectory and prognosis. Overall, these data indicate that moderate AS is not a benign entity; thereby challenging the current “watchful wait” approach currently advocated for many patients [6]. Specifically, we demonstrated a high risk of short- and longer-term mortality associated with moderate AS that warrants further research to understand how to optimally monitor and, if necessary, intervene, in affected individuals. The urgency to apply a more nuanced and cost-effective approach to AS management will only increase as the number of individuals living with this deadly condition inevitably rises in many countries [14].

Critically, the reported perioperative and periprocedural risk for AVR has declined significantly [15] from historical mortality rates of 15% [16] to approximately 1%, with residual risk dependent on individual patient factors [17]. In this context, the risk-benefit relationship between intervention and watchful waiting requires careful consideration in light of the mortality observed in this and other studies. As acknowledged by the recent European guidelines for the management of AS [8], there has been increasingly compelling evidence to suggest poor overall survival in individuals with non-severe forms of AS [7, 18–22], with 75% either dying or requiring AVR by 5-years [7]. However, many of these studies have been limited by low enrolment and short interval follow-up. In the largest study to date of moderate AS, the NEDA Investigators demonstrated the risk of all-cause and cardiovascular-related mortality was similar in those with moderate and severe AS [7]. Though hypothesis generating, this study lacked the detailed clinical granularity required to account for confounding by associated clinical variables [22], particularly the presence of CAD (with or without concurrent HF), which is common in the AS population due to shared pathophysiology [2]. In the current study, adjusting for presence of CAD, HF and more than 30 clinical, medication, and laboratory variables, we confirm a continuum of increased risk of all-cause and cardiovascular-related mortality with increasing AS severity observed in a previous iteration of the NEDA cohort [7]. On a fully adjusted basis, the risk of death in adults with moderate and severe AS was similar. These results remained substantially unchanged when considering individuals <65 years old at the time of their index echocardiogram, using an individual’s first or last echocardiogram to define AS severity, or when employing a traditional AVA-based classification scheme [11]. Moreover, though absolute risk of both all-cause and cardiovascular-related mortality was higher amongst those with a lower LVEF, the greater risk of those with moderate AS persisted across all LVEF subgroups. Cardiovascular-related mortality accounted for most but not all of the risk in this population.

The reasons for the elevated risk of mortality we observed remain unclear. First, it is possible that unmeasured clinical variables contribute to an individual’s risk of dying with (or from) AS. Second, it is possible that individuals with moderate AS may progress to severe AS prior to death without an interval echocardiogram (such progression being observed previously within the NEDA cohort with multiple investigations [3]). Third, moderate AS may contribute directly to the substantial cardiac morbidity and mortality observed in this population via specific cardiac structural changes such as ventricular fibrosis or ischemia [23]. The observed difference in mortality between the Australian (lower) and US cohort (higher) according to the severity of AS cannot be fully explored; especially without knowing the full clinical profile of the Australian cohort. However, as reflected in a higher proportion of cases with mild AS and being largely investigated in an outpatient rather than inpatient setting, the Australian cohort is likely to have less clinically significant comorbidity contributing to their risk of dying.

Regardless of the causal mechanism, the high risk of mortality amongst those with moderate AS suggests the need for closer surveillance in this subgroup and referral to a Heart Valve Clinic when indicated: noting the resource implications for such management [14]. Furthermore, these results support a role for ongoing prospective trials in those with moderate AS to assess the role of AVR in mitigating the observed risk. In the current study, regardless of left ventricular ejection fraction, stroke volume, or AVA, there was a continuum of increased risk with increasing AS severity suggesting the importance of the haemodynamic sequelae of valvular stenosis to overall prognosis. Indeed, as AVA is considered to be prognostic only in those with preserved transvalvular flow [24] and several concerns exist about the accuracy of echocardiographic AVA-estimation [25, 26], these results support an increased role for both a gradient and flow-based AS classification scheme.

Though large and multicentre, there are several limitations of the current study to consider. First, as a retrospective study, causality cannot be inferred with the current methods. Second, findings may not generalise to countries where cardiovascular disease management may significantly differ. Third, the association of symptoms attributable to AS (e.g. HF, syncope, or angina) and outcomes could not be fully assessed in the current study. Fourth, the presence of risk in individuals with moderate AS does not imply benefit from AVR and warrants further study. Fifth, although we observed in the Australian cohort that cardiovascular mortality accounted for the majority of all-cause mortality, especially amongst those with a depressed LVEF, it was not feasible to adjust for clinical confounders in the analysis of cardiovascular mortality and despite efforts to comprehensively adjust for relevant cardiovascular and non-cardiovascular causes of mortality (e.g. cancer, heart failure, coronary artery disease) in the US cohort, it is possible that residual confounding may be present. Sixth, it was not possible to review autopsies of deceased patients and thus the cause of cardiovascular death in this population should be further examined in future study. Finally, as discussed above, despite the similarity in outcome between moderate and severe AS observed in the two cohorts, the small differences observed may have been due to the predominantly inpatient-derived US echocardiography cohort and predominantly outpatient Australian cohort.

## Conclusion

In this international observational study, enrolling patients from two geographically diverse cohorts, there was a continuum of increased risk observed with increasing AS severity. Accounting for a range of potential confounders, including the valve orifice area, history of CAD and/or coronary revascularization, and more than 30 other clinical, laboratory, and medication variables, we found that the mortality risk of moderate and severe AS was very similar. While causality cannot be fully determined, these data nevertheless indicate that individuals with moderate AS are at high (short- and long-term) risk of mortality. In addition to reinforcing recent recommendations to closely monitor such patients for disease progression [8], they also support the need for prospective trials to evaluate if this high risk of mortality, currently managed on a “watchful waiting” basis, can be more proactively mitigated with AVR.

## Supporting information

S1 Fig Rationale for parallel cohort design of current study. In the absence of large, complete registries of echocardiographic report data, we set about to run two parallel analyses, leveraging the strengths of each dataset to help address the study hypothesis. In the Australian cohort, the National Echocardiographic Database of Australia, information from a total of 631,824 patients from 23 centers across Australia with TTE report data were linked to death information. The NEDA dataset is the largest echocardiographic dataset in the world and spans both academic and community sites, all age groups, and is linked to specific cause of death information from the Australian National Death Index. It reflects a largely outpatient population receiving echocardiograms. The NEDA dataset is lacking in clinical information, however. The US Cohort is the only echocardiographic dataset (to our knowledge) linked to complete Medicare claims, permitting granular clinical phenotyping (including information on demographic, clinical, echocardiographic, laboratory, and medication variables) for individuals aged 65 or older. As the US cohort is single-center, reflects more of an inpatient population receiving echocardiograms, includes a more narrow age-group, and does not have cause of death information, the union of the two datasets complements deficiencies in each and serves as the rationale for the current parallel-cohort design.
(PDF)

S2 Fig Kaplan Meier curve of time to all cause mortality by AS stage, stratified by coronary artery disease status in the US cohort. Displayed are the results of adjusted Kaplan-Meier curves evaluating the risk of all-cause mortality over 10-years (estimates are truncated at 10 years) from the last echocardiogram, stratified by presence (S2A; left) or absence (S2B; right) of coronary artery disease (defined as history of ischemic heart disease, percutaneous coronary intervention, or coronary artery bypass grafting), according to baseline aortic stenosis severity in both the US cohorts. Models are adjusted for age, sex, race, left ventricular ejection fraction, and presence of left heart disease. Individuals with no aortic stenosis (AS) are shown in red, mild in green, moderate in orange, and severe in black. The numbers in the risk set at each time point are listed above the x-axis.
(PDF)

S3 Fig Kaplan Meier curve of time to all cause mortality by AS stage, stratified by heart failure disease status in the US cohort. Displayed are the results of adjusted Kaplan-Meier curves evaluating the risk of all-cause mortality over 10-years (estimates are truncated at 10 years) from the last echocardiogram, stratified by presence (S3A; left) or absence (S3B; right) of heart failure, according to baseline aortic stenosis severity in both the US cohorts. Models are adjusted for age, sex, race, left ventricular ejection fraction, and presence of left heart disease. Individuals with no aortic stenosis (AS) are shown in red, mild in green, moderate in orange, and severe in black. The numbers in the risk set at each time point are listed above the x-axis.
(PDF)

S4 Fig Kaplan Meier curve of time to all cause mortality by AS stage using an AVA-based classification. Displayed are the results of adjusted Kaplan-Meier curves evaluating the risk of all-cause mortality over 10-years (estimates are truncated at 10 years) from the last echocardiogram, using a traditional AVA-based classification scheme, according to baseline aortic stenosis severity in both the Australian (left) and US (right) cohorts in the fully adjusted models. Individuals with no aortic stenosis (AS) are shown in red, mild in green, moderate in orange, and low-gradient severe in purple, and high-gradient severe in black. The numbers in the risk set at each time point are listed above the x-axis.
(PDF)

S1 Table Definition and sources of covariate information. (PDF)

S2 Table Method for calculating time in stage. (PDF)

S3 Table Supplementary methods describing model development for the main analysis. (PDF)

S4 Table Supplementary methods describing model development for supplemental analyses. (PDF)

S5 Table Comparison of baseline characteristics of individuals in the us cohort with missing and non-missing aortic valve area measurements. Displayed is a comparison of baseline characteristics at the time of the last echocardiogram for individuals with nonmissing and missing aortic valve area measurements in the US cohort. Number of observations represents the number with nonmissing observations for each variable. All estimates are listed as means ± standard deviations unless otherwise indicated. Cell values < 11 are suppressed per Medicare Data Use policy.
(PDF)

S6 Table Baseline characteristics of included patients (Australian cohort). Listed is the comparison of baseline characteristics at the time of an individual’s last known echocardiogram across aortic stenosis stages in the Australian cohort. Values are presented as means ± standard deviations unless otherwise indicated. AS = aortic stenosis, N = number.
(PDF)

S7 Table Results of model 1: Univariate hazard ratios for all-cause mortality. Displayed are the univariate hazard ratios for all-cause mortality in the US and Australian cohorts according to AS stage. The US model included 30,865 patients with 14,481 deaths and 16,384 censored individuals. The Australian model includes 217,599 patients with 89,064 deaths and 128,535 censored patients. All comparisons are significant at a p < 0.001 level.
(PDF)

S8 Table Univariate comparisons of individuals dead vs. alive/censored at 10-years in the Australian cohort. Represents the univariate comparison of individuals who were alive/censored at 10-years after the last echocardiogram or dead in the Australian Cohort. P < 0.001 for all univariate comparisons. Time in AS stage also calculable in 56,026 cases. CI = confidence interval, LA = left atrial, LV = left ventricular, TR = tricuspid regurgitant.
(PDF)

S9 Table Univariate comparisons of individuals dead vs. alive/censored at 10-years in the US cohort. Represents the univariate comparison of individuals who were alive/censored at 10-years after the last echocardiogram or dead in the US Cohort. p < 0.001 for all comparisons except diuretic use (p = 0.004), AR severity (p = 0.11), and history of revascularization (p = 0.10). CI = confidence interval, N = number.
(PDF)

S10 Table Results of model 4: Adjustment for aortic valve area as a continuous variable in the Australian cohort. Displayed are the results of model 4 evaluating adjustment for aortic valve area as a continuous variable in the Australian cohort. The model is adjusted for age, sex, presence of left heart disease, left ventricular ejection fraction, AS severity, as well as aortic valve area as a continuous variable. The Australian model included 92,761 patients with complete profiling with 37,155 deaths and 55,606 censored individuals. In the Australian cohort, all comparisons are significant at a p < 0.001 level, except for aortic valve area (p = 0.001). AS = aortic stenosis, CI = confidence interval.
(PDF)

S11 Table Results of model 5A: Fully adjusted model in the Australian cohort. Displayed are the results of model 5A which represents the fully adjusted model in the Australian cohort. This model is adjusted for age, sex, body mass index, presence of left heart disease, left ventricular ejection fraction, stroke volume index, time in stage, aortic valve area as a continuous measure, and AS severity. The model included 26,659 individuals with complete profiling with 9,633 deaths and 17,026 censored individuals. All comparisons are significant at a p < 0.001 level. Directly comparing severe to moderate AS with regard to risk of all-cause death (1,553 individuals included in model with 941 deaths and 612 censored individuals), the adjusted hazard ratio was 0.99 (95% CI 0.85 to 1.15), p = 0.88. TR = tricuspid regurgitant.
(PDF)

S12 Table Results of model 5B: Fully Adjusted model in the US cohort. Displayed are the results of model 5B which represents the fully adjusted model in the US cohort. This model is adjusted for age, sex, race, body mass index, peak tricuspid regurgitant velocity, presence of left heart disease, left ventricular ejection fraction, AS severity, estimated glomerular filtration rate, history of mitral or tricuspid valve interventions, inpatient status, diabetes, hypertension, hyperlipidemia, smoking, chronic obstructive pulmonary disease, chronic kidney disease, ischemic heart disease, peripheral arterial disease, history of percutaneous coronary intervention or coronary artery bypass grafting, pacemaker or implantable defibrillator, atrial fibrillation or flutter, heart failure, stroke or transient ischemic attack, dementia, anemia, cancer, cholesterol medications, antiplatelet medications, anticoagulants, beta blockers, renin-angiotensin-neprilysn inhibitors, other antihypertensives, diuretics, anti-arrhythmic medications, insulins, other diabetic medications, nitrates, digoxin, psychiatric medications, anti-inflammatory use, and other medications. The model included 14,060 individuals with complete profiling with 7,470 deaths and 6,590 censored individuals. All comparisons are significant at a p < 0.001 level except for sex (p = 0.005), left ventricular ejection fraction (p = 0.02), estimated glomerular filtration rate (p = 0.40), black race (p = 0.11), mitral valve intervention (p = 0.25), tricuspid valve intervention (p = 0.58), ischemic heart disease (p = 0.10), pacer or implantable defibrillator (p = 0.001), atrial fibrillation/flutter (p = 0.23), cholesterol medication (p = 0.002), antiplatelet medications (p = 0.43), anticoagulants (p = 0.56), beta blockers (p = 0.46), other anti-hypertensive medications (p = 0.04), diuretics (p = 0.02), anti-arrhythmic medications (p = 0.02), other anti-diabetic medications (p = 0.36), nitrates (p = 0.20), digoxin/digitalis (p = 0.21), anti-inflammatories (p = 0.35), and other medications (p = 0.03). TR = tricuspid regurgitant.
(PDF)

S13 Table Results of model 6: Sensitivity analysis using an aortic valve area classification schema. Displayed are the results of model 6, the results of a sensitivity analysis using an aortic valve area classification scheme to reclassify individuals with velocities/gradients in the mild to moderate range who have an AVA < 1.0 cm^2^ as severe low gradient AS. These models are adjusted for age, sex, race (US cohort only), presence of left heart disease, left ventricular ejection fraction and AS severity. In the US cohort, there were 30,693 individuals with complete profiling with 14,451 deaths and 16,242 censored individuals. After full adjustment for all variables in model 5B (see S1 Raw images), there were 14,060 individuals with complete profiling of which 7,470 died and 6,590 were censored. Compared to no AS, the hazard ratio was 1.25 (95% CI 1.16–1.35) for mild AS, 1.33 (95% CI 1.16–1.52) for moderate AS, 1.45 (95% CI 1.25–1.67) for severe low-gradient AS, and 1.37 (95% CI 1.17–1.59) for severe high gradient AS. All comparisons in both models were significant at a p < 0.001 level. In the Australian cohort, there were 181,343 individuals with complete profiling with 72,928 deaths and 108,415 censored individuals. After full adjustment for age, sex, body mass index, left ventricular ejection fraction, presence of left heart disease, stroke volume index, and tricuspid regurgitant velocity (26,659 with complete profiling with 9,633 all-cause deaths and 17,026 censored individuals), compared to no AS, the hazard ratio was 1.39 (95% CI 1.29–1.49) for mild AS, 1.81 (95% CI 1.59–2.06) for moderate AS, 1.73 (95% CI 1.55–1.94) for severe low gradient AS, and 2.02 (95% CI 1.77–2.30) for severe high gradient AS. The hazard ratio for aortic valve area (for a 1-cm^2^ increase) in this fully adjusted model was 0.90 (95% CI 0.88–0.94). All comparisons in both models were significant at a p < 0.001 level.
(PDF)

S14 Table Results of model 7: Sensitivity analysis reporting results for individuals under age 65 at the time of echocardiogram in the Australian cohort. Displayed are the results of model 7, the results of a sensitivity analysis amongst individuals < 65 at the time of echocardiography in the Australian cohort. A total of 235,562 individuals were < 65 years at the time of echocardiography (124,491 men [mean age 48.2 ± 12.6 years]; 111,071 females [mean age 46.7; mean age ± 13.0 years) of which 55,125 had complete profiling and were included in the model. This model is adjusted for age, sex, presence of left heart disease, left ventricular ejection fraction, and AS severity. Of the 55,125 individuals with complete profiling, there were 7,729 deaths and 47,396 censored individuals. All comparisons are significant at a p < 0.001 level except for females vs. males (p = 0.17).
(PDF)

S15 Table Results of model 9: Sensitivity analysis reporting results for the relationship of as severity and all-cause mortality using an individual’s first echocardiogram as index. Displayed are the results of model 9, results of a sensitivity analysis, using an individual’s first rather than last echocardiogram as the index to define AS severity stage. Models are adjusted for age, sex, race (US cohort only), presence of left heart disease, left ventricular ejection fraction and AS severity. In the US cohort, there were 29,930 individuals with complete profiling of which 14,373 died and 15,557 were censored. In the Australian cohort, there were 181,343 individuals with complete profiling of which 72,928 died and 108,415 were censored. All comparisons are significant at a p < 0.001 level.
(PDF)

S16 Table Results of model 10: Sensitivity analysis reporting results for the relationship of as severity and all-cause mortality with and without adjustment for known time in AS stage in the US cohort. Displayed are the results of model 10, results of a sensitivity analysis evaluating the impact of adjustment for known time in AS stage in the US cohort. Both models are adjusted for age, sex, race, body mass index, peak tricuspid regurgitant velocity, presence of left heart disease, left ventricular ejection fraction, AS severity, estimated glomerular filtration rate, history of mitral or tricuspid valve interventions, inpatient status, diabetes, hypertension, hyperlipidemia, smoking, chronic obstructive pulmonary disease, chronic kidney disease, ischemic heart disease, peripheral arterial disease, history of percutaneous coronary intervention or coronary artery bypass grafting, pacemaker or implantable defibrillator, atrial fibrillation or flutter, heart failure, stroke or transient ischemic attack, dementia, anemia, cancer, cholesterol medications, antiplatelet medications, anticoagulants, beta blockers, renin-angiotensin-neprilysn inhibitors, other antihypertensives, diuretics, anti-arrhythmic medications, insulins, other diabetic medications, nitrates, digoxin, psychiatric medications, anti-inflammatory use, and other medications. Both models included 14,060 individuals with complete profiling with 7,470 deaths and 6,590 censored individuals. All comparisons are significant at a p < 0.001 level except for sex (p = 0.005), left ventricular ejection fraction (p = 0.02), estimated glomerular filtration rate (p = 0.40), black race (p = 0.11), mitral valve intervention (p = 0.25), tricuspid valve intervention (p = 0.58), ischemic heart disease (p = 0.10), pacer or implantable defibrillator (p = 0.001), atrial fibrillation/flutter (p = 0.23), cholesterol medication (p = 0.002), antiplatelet medications (p = 0.43), anticoagulants (p = 0.56), beta blockers (p = 0.46), other anti-hypertensive medications (p = 0.04), diuretics (p = 0.02), anti-arrhythmic medications (p = 0.02), other anti-diabetic medications (p = 0.36), nitrates (p = 0.20), digoxin/digitalis (p = 0.21), anti-inflammatories (p = 0.35), and other medications (p = 0.03). TR = tricuspid regurgitant.
(PDF)

S17 Table Results of model 10: Sensitivity analysis reporting results for the relationship of as severity and all-cause mortality with and without adjustment for known time in AS stage in the Australian cohort. Displayed are the results of model 10, results of a sensitivity analysis evaluating the impact of adjustment for known time in AS stage in the Australian cohort. Models are adjusted for age, sex, body mass index, peak tricuspid regurgitant velocity, presence of left heart disease, left ventricular ejection fraction, aortic valve area, stroke volume index, and AS severity. Both models included 26,633 individuals with 9,424 deaths and 17,209 censored individuals. All comparisons are significant at a p < 0.001 level. TR = tricuspid regurgitant.
(PDF)

S18 Table Results of a model 11: Sensitivity analysis stratifying by presence or absence of coronary artery disease in the US cohort. Displayed are the results of model 11, results of a sensitivity analysis stratifying by presence or absence of coronary artery disease (defined as ischemic heart disease or history of percutaneous coronary intervention or coronary artery bypass grafting) in the US cohort. The models are adjusted for age, sex, race, presence of left heart disease, left ventricular ejection fraction and AS severity. Of the 15,272 individuals with coronary artery disease and complete profiling, 8,940 died and 6,332 were censored. Of 15,421 individuals with complete profiling but without coronary artery disease, 5,511 died and 9,910 were censored. The p-value for interaction between presence of coronary artery disease and AS severity = 0.007. All comparisons significant at a p < 0.001 level.
(PDF)

S19 Table Results of a model 12: Sensitivity analysis stratifying by presence or absence of heart failure in the US cohort. Displayed are the results of model 12, a sensitivity analysis stratifying by presence or absence of precedent heart failure in the US cohort. Models are adjusted for age, sex, race, presence of left heart disease, left ventricular ejection fraction and AS severity. Of the 12,668 individuals with complete profiling and precedent heart failure, 8,336 died and 4,332 were censored. All comparisons are significant at a p < 0.001 level except for left heart disease (p = 0.65). Of the 18,025 individuals with complete profiling but absence of precedent heart failure, 6,115 died and 11,910 were censored. All comparisons were significant at a p < 0.001 level except for black race (p = 0.94). The p-value for interaction between presence of heart failure and AS severity = 0.0014.
(PDF)

S1 Raw images (PDF)
