# Supplementary material for: Increasing risk of mortality across the spectrum of aortic stenosis is independent of comorbidity & treatment: An international, parallel cohort study of 248,464 patients
Source: PLoS One. 2022 Jul 11;17(7):e0268580. doi: 10.1371/journal.pone.0268580 (PMC9273084; doi:10.1371/journal.pone.0268580)
Supplement: S1 Fig — In the absence of large, complete registries of echocardiographic report data, we set about to run two parallel analyses, leveraging the strengths of each dataset to help address the study hypothesis. In the Australian cohort, the National Echocardiographic Database of Australia, information from a total of 631,824 patients from 23 centers across Australia with TTE report data were linked to death information. The NEDA dataset is the largest echocardiographic dataset in the world and spans both academic and community sites, all age groups, and is linked to specific cause of death information from the Australian National Death Index. It reflects a largely outpatient population receiving echocardiograms. The NEDA dataset is lacking in clinical information, however. The US Cohort is the only echocardiographic dataset (to our knowledge) linked to complete Medicare claims, permitting granular clinical phenotyping (including information on demographic, clinical, echocardiographic, laboratory, and medication variables) for individuals aged 65 or older. As the US cohort is single-center, reflects more of an inpatient population receiving echocardiograms, includes a more narrow age-group, and does not have cause of death information, the union of the two datasets complements deficiencies in each and serves as the rationale for the current parallel-cohort design. (PDF) [file pone.0268580.s001.pdf]

S1 Fig. Rationale for Parallel Cohort Design of Current Study

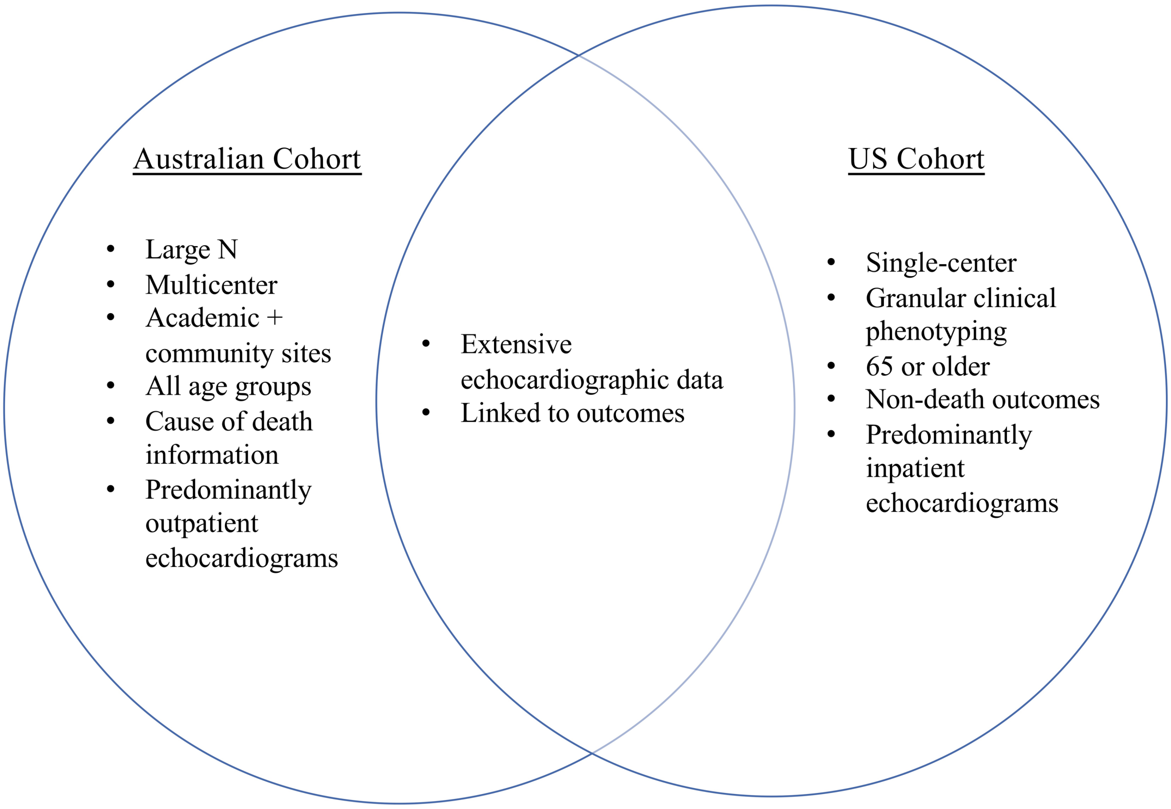

In the absence of large, complete registries of echocardiographic report data, we set about to run two parallel analyses, leveraging the strengths of each dataset to help address the study hypothesis. In the Australian cohort, the National Echocardiographic Database of Australia, information from a total of 631,824 patients from 23 centers across Australia with TTE report data were linked to death information. The NEDA dataset is the largest echocardiographic dataset in the world and spans both academic and community sites, all age groups, and is linked to specific cause of death information from the Australian National Death Index. It reflects a largely outpatient population receiving echocardiograms. The NEDA dataset is lacking in clinical information, however. The US Cohort is the only echocardiographic dataset (to our knowledge) linked to complete Medicare claims, permitting granular clinical phenotyping (including information on demographic, clinical, echocardiographic, laboratory, and medication variables) for individuals aged 65 or older. As the US cohort is single-center, reflects more of an inpatient population receiving echocardiograms, includes a more narrow age-group, and does not have cause of death information, the union of the two datasets complements deficiencies in each and serves as the rationale for the current parallel-cohort design.
