# Supplementary material for: Increasing risk of mortality across the spectrum of aortic stenosis is independent of comorbidity & treatment: An international, parallel cohort study of 248,464 patients
Source: PLoS One. 2022 Jul 11;17(7):e0268580. doi: 10.1371/journal.pone.0268580 (PMC9273084; doi:10.1371/journal.pone.0268580)
Supplement: S3 Fig — Displayed are the results of adjusted Kaplan-Meier curves evaluating the risk of all-cause mortality over 10-years (estimates are truncated at 10 years) from the last echocardiogram, stratified by presence (S3A; left) or absence (S3B; right) of heart failure, according to baseline aortic stenosis severity in both the US cohorts. Models are adjusted for age, sex, race, left ventricular ejection fraction, and presence of left heart disease. Individuals with no aortic stenosis (AS) are shown in red, mild in green, moderate in orange, and severe in black. The numbers in the risk set at each time point are listed above the x-axis. (PDF) [file pone.0268580.s003.pdf]

S3 Fig. Kaplan Meier Curve of Time to All-Cause Mortality by AS Stage, Stratified by Heart Failure Disease Status in the US Cohort

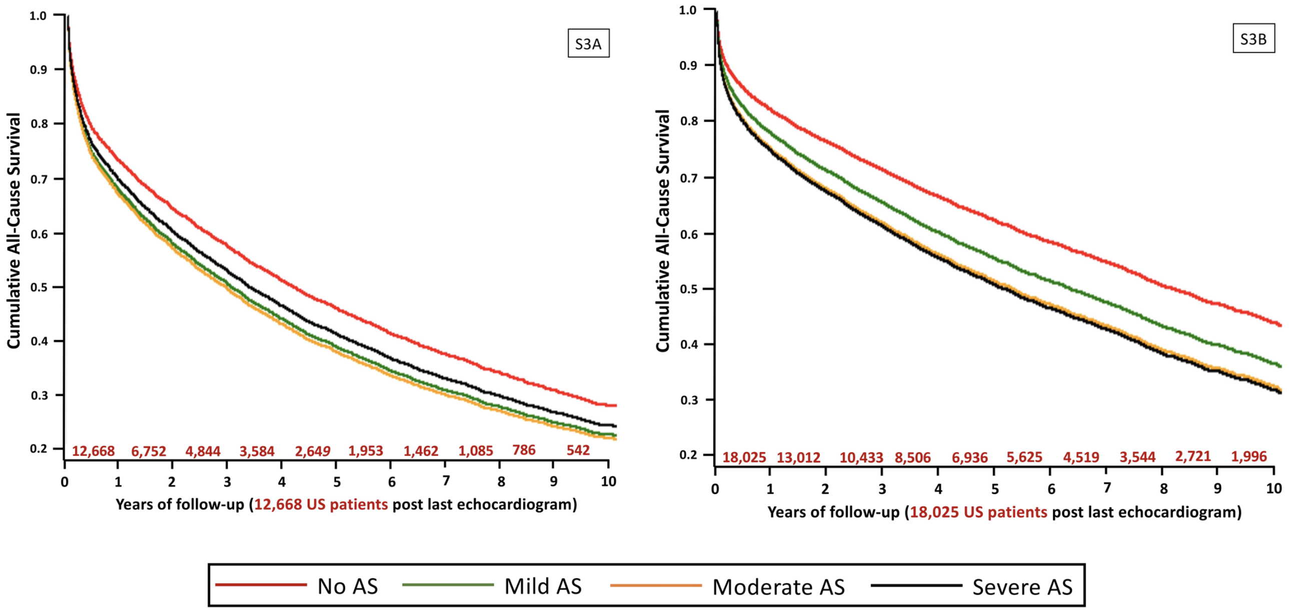

Displayed are the results of adjusted Kaplan-Meier curves evaluating the risk of all-cause mortality over 10-years (estimates are truncated at 10 years) from the last echocardiogram, stratified by presence (S3A; left) or absence (S3B; right) of heart failure, according to baseline aortic stenosis severity in both the US cohorts. Models are adjusted for age, sex, race, left ventricular ejection fraction, and presence of left heart disease. Individuals with no aortic stenosis (AS) are shown in red, mild in green, moderate in orange, and severe in black. The numbers in the risk set at each time point are listed above the x-axis.
