# Supplementary material for: Increasing risk of mortality across the spectrum of aortic stenosis is independent of comorbidity & treatment: An international, parallel cohort study of 248,464 patients
Source: PLoS One. 2022 Jul 11;17(7):e0268580. doi: 10.1371/journal.pone.0268580 (PMC9273084; doi:10.1371/journal.pone.0268580)
Supplement: S4 Fig — Displayed are the results of adjusted Kaplan-Meier curves evaluating the risk of all-cause mortality over 10-years (estimates are truncated at 10 years) from the last echocardiogram, using a traditional AVA-based classification scheme, according to baseline aortic stenosis severity in both the Australian (left) and US (right) cohorts in the fully adjusted models. Individuals with no aortic stenosis (AS) are shown in red, mild in green, moderate in orange, and low-gradient severe in purple, and high-gradient severe in black. The numbers in the risk set at each time point are listed above the x-axis. (PDF) [file pone.0268580.s004.pdf]

S4 Fig. Kaplan Meier Curve of Time to All-Cause Mortality by AS Stage Using an AVA-Based Classification

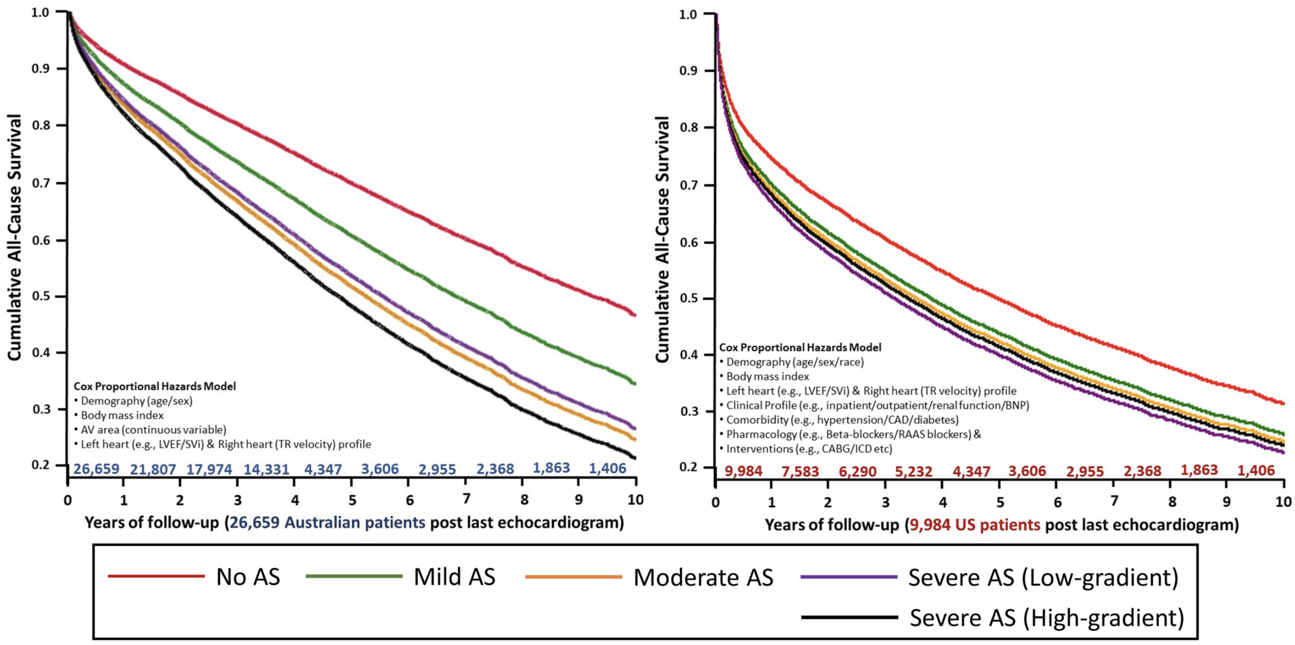

Displayed are the results of adjusted Kaplan-Meier curves evaluating the risk of all-cause mortality over 10-years (estimates are truncated at 10 years) from the last echocardiogram, using a traditional AVA-based classification scheme, according to baseline aortic stenosis severity in both the Australian (left) and US (right) cohorts in the fully adjusted models. Individuals with no aortic stenosis (AS) are shown in red, mild in green, moderate in orange, and low-gradient severe in purple, and high-gradient severe in black. The numbers in the risk set at each time point are listed above the x-axis.
