# Supplementary material for: Increasing risk of mortality across the spectrum of aortic stenosis is independent of comorbidity & treatment: An international, parallel cohort study of 248,464 patients
Source: PLoS One. 2022 Jul 11;17(7):e0268580. doi: 10.1371/journal.pone.0268580 (PMC9273084; doi:10.1371/journal.pone.0268580)
Supplement: S1 Table — (PDF) [file pone.0268580.s005.pdf]

S1 Table. Definition and Sources of Covariate Information

Variable	Definition	Timing	Data Source	Cohorts Available
**Demographics**				
Age	Age in years	At the time of TTE	TTE report	Australia + US
Sex	Self-assigned sex	At the time of TTE	TTE report	Australia + US
Race	Self-assigned race: black, white, or other	At the time of TTE	Medicare beneficiary summary file	US
Blood pressure	Systolic and diastolic blood pressures in mmHg	At the time of TTE	TTE report	Australia + US
Heart rate	Heart rate in beats per minute	At the time of left ventricular outflow tract VTI acquisition	TTE report	Australia + US
Height	Reported height (cm)	At the time of TTE	TTE report	Australia + US
Weight	Reported weight (kg)	At the time of TTE	TTE report	Australia + US
Body surface area	Defined using the Mosteller formula[1] (m^2)	At the time of TTE	TTE report	Australia + US
Body mass index	Weight per height-squared (kg/m^2)	At the time of TTE	TTE report	Australia + US
Inpatient/outpatient status	Status of patient (admitted to inpatient facility or not)	At the time of TTE	TTE report	US
**Echocardiographic Data**				
Technical quality	Defined by interpreting physician as adequate or suboptimal (≥2 or more nonvisualized segments)	At the time of TTE	TTE report	Australia + US
Left ventricular end-diastolic dimension	Left ventricular end-diastolic dimension in the apical long-axis view at the level of the mitral valve (cm)	At the time of TTE	TTE report	Australia + US
Left ventricular end-systolic dimension	Left ventricular end-systolic dimension in the apical long-axis view at the level of the mitral valve (cm)	At the time of TTE	TTE report	Australia + US
Left ventricular ejection fraction	Defined by the interpreting physician on the TTE report (includes visual assessment, biplane method of disks, and 3-dimensional volumetric quantification) using percentages.	At the time of TTE	TTE report	Australia + US
E/e’ ratio	Ratio of transmitral peak E-wave velocity and the average of septal and mitral tissue doppler e’ values	At the time of TTE	TTE report	Australia + US
E/A ratio	Ratio of the transmitral peak E-wave velocity and peak A-wave velocity	At the time of TTE	TTE report	Australia + US
Stroke volume index	Defined as stroke volume divided by body surface area. Stroke volume was defined as the left ventricular outflow tract area multiplied by the left ventricular outflow tract velocity-time-integral (mL/m^2)	At the time of TTE	TTE report	Australia + US

Left ventricular outflow tract velocity/VTI	Peak left ventricular outflow tract velocity or velocity-time-integral (m/s for velocity, cm for VTI)	At the time of TTE	TTE report	Australia + US
Left ventricular mass index	Left ventricular mass was defined using the Devereux formula[2] and divided by body surface area (g/m^2)	At the time of TTE	TTE report	Australia + US
Peak tricuspid regurgitant velocity	Peak tricuspid regurgitant Doppler velocity (m/s)	At the time of TTE	TTE report	Australia + US
Right ventricular basal dimension	Measurement of the basal right ventricle in a modified right ventricular focused apical 4-chamber view at end-diastole (cm)	At the time of TTE	TTE report	Australia + US
Right atrial length	Maximal length from annulus to right atrial roof in the apical 4-chamber view at end-systole (cm)	At the time of TTE	TTE report	Australia + US
Left atrial volume index	Biplane left atrial volume (obtained in apical 4 and 2 chamber views) divided by body surface area (mL/m^2)	At the time of TTE	TTE report	Australia + US
Aortic regurgitation severity	Semiquantitative measure of aortic regurgitation severity (0+, 1+, 2+, 3+, 4+) by interpreting physician using an integrative approach	At the time of TTE	TTE report	Australia + US
Mitral regurgitation severity	Semiquantitative measure of mitral regurgitation severity (0+, 1+, 2+, 3+, 4+) by interpreting physician using an integrative approach	At the time of TTE	TTE report	Australia + US
Presence of a bicuspid aortic valve	Bicuspid or bicommissural aortic valve on visual inspection	At the time of TTE	TTE report	Australia + US
Transmitral mean pressure gradient	Mean pressure gradient across the mitral valve during diastole, using the VTI of pulsed-wave Doppler at the mitral leaflets tips	At the time of TTE	TTE report	Australia + US
Peak aortic velocity	Peak transaortic velocity (m/s)	At the time of TTE	TTE report	Australia + US
Mean aortic valve gradient	Mean transaortic gradient during systole using the VTI of the aortic Doppler (mmHg). In the US cohort, mean gradient (per lab protocol) was only recorded in those with a peak aortic velocity > 2.0 m/s.	At the time of TTE	TTE report	Australia + US
Aortic valve area	Calculated aortic valve area using the continuity equation (cm^2). In the US cohort, aortic valve area (per lab protocol) was only recorded in those with a peak aortic velocity > 2.0 m/s.	At the time of TTE	TTE report	Australia + US
Left heart disease	Defined as either left ventricular ejection fraction < 55%, transmitral E/e' > 12.0, left atrial volume index > 34.0 mL/m^2, or presence of left sided valvular heart disease (transmitral mean gradient ≥ 5 mmHg, 2+ or greater aortic or mitral regurgitation)	At the time of TTE	TTE report	Australia + US
**Clinical Variables**				
Diabetes Mellitus	Presence of an inpatient or outpatient claim for diabetes mellitus using Medicare Chronic Condition Warehouse validated algorithm[3-4]	Within 2-years prior to TTE	Medicare inpatient and outpatient claims	US

Hypertension	Presence of an inpatient or outpatient claim for hypertension using Medicare Chronic Condition Warehouse validated algorithm[4,5-9]	Within 1-year prior to TTE	Medicare inpatient and outpatient claims	US
Hyperlipidemia	Presence of an inpatient or outpatient claim for hyperlipidemia using Medicare Chronic Condition Warehouse validated algorithm[9-11]	Within 1-year prior to TTE	Medicare inpatient and outpatient claims	US
Smoking	Presence of an inpatient or outpatient claim for smoking using Medicare Chronic Condition Warehouse validated algorithm[12]	Within 2-years prior to TTE	Medicare inpatient and outpatient claims	US
Chronic obstructive pulmonary disease	Presence of an inpatient or outpatient claim for chronic obstructive pulmonary disease using Medicare Chronic Condition Warehouse validated algorithm[13]	Within 1-year prior to TTE	Medicare inpatient and outpatient claims	US
Chronic kidney disease	Presence of an inpatient or outpatient claim for chronic kidney disease using Medicare Chronic Condition Warehouse validated algorithm[14-16]	Within 2-years prior to TTE	Medicare inpatient and outpatient claims	US
Ischemic heart disease	Presence of an inpatient or outpatient claim for ischemic heart disease using Medicare Chronic Condition Warehouse validated algorithm[17-18]	Within 2-years prior to TTE	Medicare inpatient and outpatient claims	US
Peripheral arterial disease	Presence of an inpatient claim for peripheral arterial disease using the Charlson Comorbidity Index[19]	Within 2-years prior to TTE	Medicare inpatient and outpatient claims	US
Atrial fibrillation	Presence of an inpatient or outpatient claim for atrial fibrillation using Medicare Chronic Condition Warehouse validated algorithm[20-21]	Within 1-year prior to TTE	Medicare inpatient and outpatient claims	US
Heart failure	Presence of an inpatient or outpatient claim for heart failure using Medicare Chronic Condition Warehouse validated algorithm[4,22]	Within 2-years prior to TTE	Medicare inpatient and outpatient claims	US
Ischemic stroke / transient ischemic attack	Presence of an inpatient claim using validated algorithms for ischemic stroke or transient ischemia attack[23]	Within 2-years prior to TTE	Medicare inpatient and outpatient claims	US
Dementia	Presence of an inpatient or outpatient claim for dementia using Medicare Chronic Condition Warehouse validated algorithm[24]	Within 2-years prior to TTE	Medicare inpatient and outpatient claims	US
Anemia	Presence of an inpatient or outpatient claim for anemia using Medicare Chronic Condition Warehouse validated algorithm[25-26]	Within 1-year prior to TTE	Medicare inpatient and outpatient claims	US
Cancer	Presence of an inpatient or outpatient claim for lung, prostate, breast, colorectal, or endometrial cancer using Medicare Chronic Condition Warehouse validated algorithm[27-40]	Within 1-year prior to TTE	Medicare inpatient and outpatient claims	US
**Interventions**				
History of percutaneous coronary intervention	Presence of an ICD-9-CM (17.55, 36.04, 36.06, 36.07, 36.09) or ICD-10-CM (02703DZ, 02713DZ, 02723DZ, 02733DZ, 027034Z,	Within 1-year prior to TTE	Medicare inpatient and outpatient claims	US

	027134Z, 027234Z, 027334Z) procedural code for percutaneous coronary intervention			
History of coronary artery bypass grafting	Presence of an ICD-9-CM (36.10, 36.11, 36.12, 36.13, 36.14, 36.15, 36.16, 36.17, 36.19) or ICD-10-CM procedural code for coronary artery bypass grafting[41]	Within 1-year prior to TTE	Medicare inpatient and outpatient claims	US
Presence of pacemaker or implantable cardioverter defibrillator	Presence of a pacemaker or right ventricular wire on TTE	At the time of TTE	TTE report	US
History of aortic valve replacement	Presence of an aortic valve replacement on TTE	At the time of TTE	TTE report	Australia + US
History of mitral valve replacement/repair	Presence of a mitral valve intervention (surgical or transcatheter) on TTE	At the time of TTE	TTE report	Australia + US
History of tricuspid valve replacement/repair	Presence of a tricuspid valve intervention (surgical or transcatheter) on TTE	At the time of TTE	TTE report	Australia + US
**Medications (prescribed)**				
Antiplatelets	Includes platelet-aggregation inhibitors, salicylates, and platelet-reducing agents	Within the 1 month preceding TTE	Linkage with institutional data	US
Anticoagulants	Includes coumarin derivatives, direct factor Xa inhibitors, direct thrombin inhibitors, and other anticoagulants	Within the 1 month preceding TTE	Linkage with institutional data	US
Beta blockers	Includes nonselective and selective beta-adrenergic blockers	Within the 1 month preceding TTE	Linkage with institutional data	US
Cholesterol modification agents	Includes statin medications, fibrates, bile acid sequestrants, proprotein convertase subtilisin/kexin type 9 (PCSK9) inhibitors, or other antilipidemic agents	Within the 1 month preceding TTE	Linkage with institutional data	US
Renin-angiotensin-neprilysn inhibitors	Includes angiotensin-converting enzyme inhibitors, angiotensin II receptor antagonists, renin inhibitors, and neprilysn inhibitors	Within the 1 month preceding TTE	Linkage with institutional data	US
Other anti-hypertensives	Includes miscellaneous hypotensive or vasodilating agents, calcium channel blockers, alpha-adrenergic blockers, and central alpha-adrenergic agonists	Within the 1 month preceding TTE	Linkage with institutional data	US
Anti-arrhythmic medications	Includes classes 1A, 1B, 1C, II, III, IV, and miscellaneous	Within the 1 month preceding TTE	Linkage with institutional data	US
Diuretics	Includes loop diuretics, thiazide diuretics, potassium-sparing diuretics, mineralocorticoid (aldosterone) antagonists, thiazide-like diuretics, and miscellaneous diuretics	Within the 1 month preceding TTE	Linkage with institutional data	US
Insulin	Includes short and long-acting insulins	Within the 1 month	Linkage with	US

		preceding TTE	institutional data	
Anti-diabetic medications	Includes biguanides, dipeptidyl peptidase-4 inhibitors, sulfonylureas, thiazolidinediones, alpha-glucosidase inhibitors, sodium-glucose cotransporter 2 inhibitors, and miscellaneous glycemic agents	Within the 1 month preceding TTE	Linkage with institutional data	US
Nitrates	Includes nitrates and nitrites	Within the 1 month preceding TTE	Linkage with institutional data	US
Digoxin/digitalis	Includes cardiotonic agents such as digoxin and digitalis-containing compounds	Within the 1 month preceding TTE	Linkage with institutional data	US
Psychiatric treatments	Includes selective serotonin reuptake inhibitors, anticonvulsants, antimuscarinics, tricyclic antidepressants, serotonin norepinephrine reuptake inhibitors, amphetamines, anticholinergic and parasympathomimetic agents, antipsychotic agents, and monoamine oxidase inhibitors	Within the 1 month preceding TTE	Linkage with institutional data	US
Anti-inflammatory medications	Includes salicylates, cyclooxygenase-2 inhibitors, and other non-steroidal anti-inflammatory drugs	Within the 1 month preceding TTE	Linkage with institutional data	US
Other	Includes hormonal agents (corticosteroids, estrogens, antithyroid medications), anticholinergic and beta agonist inhalers, vasopressors, antigout agents, iron agents, histamine-2 antagonists, phosphodiesterase type 5 inhibitors, phosphodiesterase type 4 inhibitors, and anorexigens	Within the 1 month preceding TTE	Linkage with institutional data	US
**Laboratory Variables**				
Hemoglobin a1c	Glycated hemoglobin (% of total hemoglobin)	Most recent value within 3 months preceding TTE	Linkage with institutional data	US
Hemoglobin	Calculated using the Sysmex ® XN Series assay (g/dL)	Most recent value within 3 months preceding TTE	Linkage with institutional data	US
Low density lipoprotein cholesterol	Fasting low-density lipoprotein (mg/dL)	Most recent value within 3 months preceding TTE	Linkage with institutional data	US
Estimated glomerular filtration rate	Using the Modification of Diet in Renal Disease (MDRD) equation, estimated using serum creatinine (mg/dL; Roche® Cobas, Creatinine (CREJ2) Assay) and reported in mL/minute	Most recent value within 3 months preceding TTE	Linkage with institutional data	US

N-terminal pro-brain natriuretic peptide	Calculated using the Roche® Cobas, N-terminal pro B-type natriuretic peptide (proBNP) Assay (pg/mL)	Most recent value within 3 months preceding TTE	Linkage with institutional data	US
Troponin T	Calculated using the Roche® Cobas, Troponin T Stat ElectroChemiLuminesnce ImmunoAssay (ECLIA) (ng/mL)	Most recent value within 3 months preceding TTE	Linkage with institutional data	US
Blood urea nitrogen	Blood urea nitrogen concentration (mg/dL)	Most recent value within 3 months preceding TTE	Linkage with institutional data	US

TTE = transthoracic echocardiogram; US = United States

References:

1) Mosteller RD. Simplified calculation of body-surface area. N Engl J Med. 198;317(17): 1098.
2) Devereux RB, Alonso DR, Lutas EM, et al. Echocardiographic assessment of left ventricular hypertrophy: comparison to necropsy findings. *Am J Cardiol.* 1986;57: 540.
3) Rector TS, Wickstrom SL, Shah M, et al. Specificity and sensitivity of claims-based algorithms for identifying members of Medicare+Choice health plans that have chronic medical conditions. *Health Serv Res.* 2004;39(6 Pt 1):1839-1857.
4) Hebert PL, Geiss LS, Tierney EF, Engelgau MM, Yawn BP, McBean AM. Identifying persons with diabetes using Medicare claims data. *Am J Med Quality.* 1999;14(6):270-277.
5) Borzecki AM, Wong AT, Hickey EC, Ash AS, Berlowitz DR. Identifying hypertension-related comorbidities from administrative data: what's the optimal approach? *Am J Med Quality.* 2004;19(5):201-206.
6) Dutro MP, Gerthoffer TD, Peterson ED, Tang SS, Goldberg GA. Treatment of hypertension and dyslipidemia or their combination among US managed-care patients. *J Clin Hypertension* 2007;9(9):684-691.
7) Halpern MT, Khan ZM, Schmier JK, et al. Recommendations for evaluating compliance and persistence with hypertension therapy using retrospective data. *Hypertension.* 2006;47(6):1039-1048.
8) Quam L, Ellis LB, Venus P, Clouse J, Taylor CG, Leatherman S. Using claims data for epidemiologic research. The concordance of claims-based criteria with the medical record and patient survey for identifying a hypertensive population. *Med Care.* 1993;31(6):498-507.
9) Black L, Runken MC, Eaddy M, Shah M. Chronic disease prevalence and burden in elderly men: an analysis of Medicare medical claims data. *J Health Care Finance.* 2007;33(4):68-78.
10) Issets BJ, Schondelmeyer SW, Artz MB, et al. Clinical and economic outcomes of medication therapy management services: the Minnesota experience. *J Am Pharmacists Assoc.* 2008;48(2):203-211; 203 p following 211.
11) Southern DA, Norris CM, Quan H, et al. An administrative data merging solution for dealing with missing data in a clinical registry: adaptation from ICD-9 to ICD-10. *BMC Med Research Methodology.* 2008;8:1.
12) Gorina Y, Kramarow EA. Identifying chronic conditions in Medicare claims data: evaluating the Chronic Condition Data Warehouse algorithm. *Health Serv Res.* 2011;46(5):1610-1627.
13) Singh JA. Accuracy of Veterans Affairs databases for diagnoses of chronic diseases. *Preventing Chronic Disease.* 2009;6(4):A126.
14) Collins AJ, Chen SC, Gilbertson DT, Foley RN. CKD surveillance using administrative data: impact on the health care system. *Am J Kidney Diseases.* 2009;53(3 Suppl 3):S27-36.
15) Foley RN, Murray AM, Li S, et al. Chronic kidney disease and the risk for cardiovascular disease, renal replacement, and death in the United States Medicare population, 1998 to 1999. *J Am Soc Nephrology.* 2005;16(2):489-495.
16) Winkelmayer WC, Schneeweiss S, Mogun H, Patrick AR, Avorn J, Solomon DH. Identification of individuals with CKD from Medicare claims data: a validation study. *Am J Kidney Diseases.* 2005;46(2):225-232.

17) DiMartino LD, Hammill BG, Curtis LH, et al. External validity of the cardiovascular health study: a comparison with the Medicare population. *Med Care.* 2009;47(8):916-923.

18) Johnson ML, Petersen LA, Sundaravaradan R, et al. The association of Medicare drug coverage with use of evidence-based medications in the Veterans Health Administration. *Ann Pharmacotherapy.* 2009;43(10):1565-1575.
19) Radanovic D, Seifert B, Urban P, et al. Validity of Charlson Comorbidity Index in patients hospitalised with acute coronary syndrome. Insights from the nationwide AMIS Plus registry 2002-2012. *Heart.* 2014;100(4):288-294.
20) Ellis ER, Culler SD, Simon AW, Reynolds MR. Trends in utilization and complications of catheter ablation for atrial fibrillation in Medicare beneficiaries. *Heart Rhythm.* 2009;6(9):1267-1273.
21) Gage BF, Boechler M, Doggette AL, et al. Adverse outcomes and predictors of underuse of antithrombotic therapy in medicare beneficiaries with chronic atrial fibrillation. *Stroke.* 2000;31(4):822-827.
22) Birman-Deych E, Waterman AD, Yan Y, Nilasena DS, Radford MJ, Gage BF. Accuracy of ICD-9-CM codes for identifying cardiovascular and stroke risk factors. *Med Care.* 2005;43(5):480-485.
23) Strom JB, Zhao Y, Faridi KF, et al. Comparison of Clinical Trials and Administrative Claims to Identify Stroke Among Patients Undergoing Aortic Valve Replacement: Findings From the EXTEND Study. *Circ Cardiovasc Interv.* 2019;12(11):e008231.
24) Taylor DH, Fillenbaum GG, Ezell ME. The accuracy of Medicare claims data in identifying Alzheimer's disease. *J Clin Epidemiol*. 2002; 55:929-937.
25) Herzog CA, Muster HA, Li S, Collins AJ. Impact of congestive heart failure, chronic kidney disease, and anemia on survival in the Medicare population. *J Cardiac Fail*; 2004:10(6), 467-472.
26) Sandgren, PE, Murray AM, Herzog CA, Solid CA, Gilbertson, DT, Collins, AJ, et al. Anemia and new-onset congestive heart failure in the general Medicare population. *J Cardiac Fail.* 2005; 11(2), 99-105.
27) Cooper GS, Yuan Z, Stange KC, Dennis LK, Amini SB, Rimm AA. The sensitivity of Medicare claims data for case ascertainment of six common cancers. *Med Care*. 1999; 37(5):436-44.
28) Cooper GS, Yuan Z, Stange KC, Rimm AA. Use of Medicare claims data to measure county-level variations in the incidence of colorectal carcinoma. *Cancer*. 1998; 83(4):673-8.
29) Freeman JL, Zhang D, Freeman DH, Goodwin JS. An approach to identifying incident breast cancer cases using Medicare claims data. *J Clin Epidemiol*. 2000; 53(6):605-14.
30) Koroukian SM, Cooper GS, Rimm AA. Ability of Medicaid claims data to identify incident cases of breast cancer in the Ohio Medicaid population. *Health Serv Res*. 2003; 38(3):947-60.
31) McBean AM, Warren JL, Babish JD. Measuring the incidence of cancer in elderly Americans using Medicare claims data. *Cancer*. 1994; 73(9):2417-25.
32) McBean AM, Babish JD, Warren JL. Determination of lung cancer incidence in the elderly using Medicare claims data. *Am J Epidemiol.* 1993; 137(2):226-34.
33) McClish D, Penberthy L, Pugh A. Using Medicare claims to identify second primary cancers and recurrences in order to supplement a cancer registry. *J Clin Epidemiol*. 2003; 56(8):760-7.
34) McClish DK, Penberthy L, Whittemore M, et al. Ability of Medicare claims data and cancer registries to identify cancer cases and treatment. *Am J Epidemiol.* 1997; 145(3):227-33.
35) McClish D, Penberthy L. Using Medicare data to estimate the number of cases missed by a cancer registry: a 3-source capture- recapture model. *Med Care.* 2004; 42(11):1111-6.
36) Moy CS, McCarty DJ, Libman IM, LaPorte RE. Determination of lung cancer incidence in the elderly using Medicare claims data. *Am J Epidemiol*. 1994; 139(4):447-9.
37) Nattinger AB, Laud PW, Bajorunaite R, Sparapani RA, Freeman JL. An algorithm for the use of Medicare claims data to identify women with incident breast cancer. *Health Serv Res*. 2004; 39(6 Pt 1):1733-49.
38) Wang PS, Walker AM, Tsuang MT, Orav EJ, Levin R, Avorn J. Finding incident breast cancer cases through US claims data and a state cancer registry. *Cancer Causes Control*. 2001; 12(3):257-65.
39) Warren JL, Feuer E, Potosky AL, Riley GF, Lynch CF. Use of Medicare hospital and physician data to assess breast cancer incidence. *Med Care*. 1999; 37(5):445-56.
40) Whittle J, Steinberg EP, Anderson GF, Herbert R. Accuracy of Medicare claims data for estimation of cancer incidence and resection rates among elderly Americans. *Med Care*. 1991; 29(12):1226-36.
41) Agency for Healtcare Research and Quality. Inpatient Quality Indicators #26 (IQI #26): Coronary Artery Bypass Graft (CABG) Rate. AHRQ Quality Indicators (AHRQ QI) ICD-9-CM and ICD-10-CM/PCS Specification Enhanced Version 5.0. October 2015. Available from: https://www.qualityindicators.ahrq.gov/Downloads/Modules/IQI/V50-ICD10/TechSpecs/IQI%2026%20Coronary%20Artery%20Bypass%20Graft%20(CABG)%20Rate.pdf
