# Supplementary material for: Increasing risk of mortality across the spectrum of aortic stenosis is independent of comorbidity & treatment: An international, parallel cohort study of 248,464 patients
Source: PLoS One. 2022 Jul 11;17(7):e0268580. doi: 10.1371/journal.pone.0268580 (PMC9273084; doi:10.1371/journal.pone.0268580)
Supplement: S2 Table — (PDF) [file pone.0268580.s006.pdf]

S2 Table. Method for Calculating Time in Stage

Time in Stage: To calculate time in stage, one can estimate the time in stage as the sum of the whole time period plus the transit time period.

STEP 1: Identify the AS severity stage for the last TTE (TTE_{last}).

STEP 2: Based on the AS stage of TTE_{last}, find the first TTE (TTE_{first}) showing the same AS stage as TTE_{last}; Calculate the whole time period in this stage as the time interval between these two TTEs.

STEP 3: After obtaining the whole time period in one stage, one must identify the transit time period:

a. If the TTE_{first} is the index TTE (for AS staging), then the transit time period would be zero. The time in stage would be the whole time period.
b. If the TTE_{first} is not the index TTE, then select the most recent TTE with the AS severity stage less severe than the TTE_{last} (TTE_{prior}) (e.g. if TTE_{first} AS severity = severe, then TTE_{prior} would be the most recent TTE with an AS severity = moderate or less). The transit time period would be half the time interval between these two TTEs (e.g. [TTE_{first} - TTE_{prior}]/2).

For analyses, the proportion of an individual's total time observed was used for multivariable adjustment. This was calculated as the time in stage divided by the total time observed (time from the date of the first to last echocardiogram).
