# Supplementary material for: Increasing risk of mortality across the spectrum of aortic stenosis is independent of comorbidity & treatment: An international, parallel cohort study of 248,464 patients
Source: PLoS One. 2022 Jul 11;17(7):e0268580. doi: 10.1371/journal.pone.0268580 (PMC9273084; doi:10.1371/journal.pone.0268580)
Supplement: S3 Table — (PDF) [file pone.0268580.s007.pdf]

S3 Table. Supplementary Methods Describing Model Development for the Main Analysis

In the main analysis, a series of nested Cox Proportional Hazards models were used to serially adjust for relevant covariates. Variables were chosen for model inclusion based on availability and clinical relevance:

1) Model 1 (both Australian and US cohorts) includes the univariate analysis of AS stage (results in **Table S7**)
2) Model 2 (both Australian and US cohorts), includes age and sex (results in **Table 2**)
3) Model 3 (both Australian and US cohorts) includes AS stage, age, sex, race (US cohort only), left ventricular ejection fraction, and presence of left heart disease (results in text)
4) Model 4 (Australian cohort only) includes aortic valve area (as a continuous measure) AS stage, age, sex, left ventricular ejection fraction, and presence of left heart disease (results in **Table S10**). In the US cohort, aortic valve area is only measured in individuals with a peak aortic velocity > 2.0 m/s as per echocardiographic laboratory policy. As such, individuals with missing and non-missing aortic valve area in the US cohort differed across important characteristics (**Table S5**). As a result, aortic valve area as a continuous measure was adjusted for only in the Australian cohort. As the same bias does not extend to use of an aortic valve area cut off in Model 6 (**Table S13**), both cohorts are included in this supplementary analysis.
5) Model 5A (fully adjusted; Australian cohort only) includes stroke volume index, aortic valve area (as a continuous measure) AS stage, age, sex, body mass index, left ventricular ejection fraction, peak tricuspid regurgitant velocity, and presence of left heart disease (results in **Table S11**)
6) Model 5B (fully adjusted; US cohort only) includes AS stage, age, sex, race, left ventricular ejection fraction, presence of mitral or tricuspid valve repair/replacement, presence of left heart disease, tricuspid regurgitant velocity, body mass index, diabetes mellitus, hypertension, hyperlipidemia, smoking, chronic obstructive pulmonary disease, chronic kidney disease, ischemic heart disease, peripheral arterial disease, history of percutaneous coronary intervention or coronary artery bypass grafting, presence of a pacemaker or implantable defibrillator, atrial fibrillation, heart failure, history of stroke or transient ischemic attack, dementia, anemia, cancer, estimated glomerular filtration rate, cholesterol medications, antiplatelet medications, anticoagulants, beta blockers, renin-angiotensin-neprilysin inhibitors, other antihypertensive medications, diuretics, anti-arrhythmic medications, insulin, other diabetic medications, nitrates, digoxin/digitalis, psychiatric medications, anti-inflammatory use, or use of other medications (results in **Table S12**).
