# Supplementary material for: Increasing risk of mortality across the spectrum of aortic stenosis is independent of comorbidity & treatment: An international, parallel cohort study of 248,464 patients
Source: PLoS One. 2022 Jul 11;17(7):e0268580. doi: 10.1371/journal.pone.0268580 (PMC9273084; doi:10.1371/journal.pone.0268580)
Supplement: S4 Table — (PDF) [file pone.0268580.s008.pdf]

S4 Table. Supplementary Methods Describing Model Development for Supplemental Analyses

In supplemental analyses, a series of Cox Proportional Hazards models were used to test assumptions the evaluate the effect of AS stage amongst relevant subgroups:

1) Model 6 (both Australian and US cohorts) – AVA-based classification - includes AS stage, age, sex, race (US cohort only), left ventricular ejection fraction, and presence of left heart disease. AS stage is determined using an aortic valve area (AVA) based classification. In this scheme, those individuals with mild-moderate AS using the prior scheme were reclassified as having severe low-gradient AS if AVA was nonmissing and < 1.0 cm^2 on the index echocardiogram. Individuals with severe AS by the prior categorization (i.e. peak velocity ≥ 4 m/s or mean gradient ≥ 40 mmHg) were classified as severe high-gradient AS (results in **Table S13**).
2) Model 7 (Australian cohort only) – Individuals < 65 years old at the time of echocardiogram - includes AS stage, age, sex, left ventricular ejection fraction, and presence of left heart disease. Mortality data for US individuals < 65 years old were not available (results in **Table S14**).
3) Model 8 (Australian cohort only) – Cardiovascular-related death - includes AS stage, age, sex, left ventricular ejection fraction, and presence of left heart disease. Information on cause-specific, cardiovascular death was not available for US individuals (results in **Table S15**).
4) Model 9 (both Australian and US cohorts) – First echocardiogram as index - includes AS stage, age, sex, race (US cohort only), left ventricular ejection fraction, and presence of left heart disease. In order to test whether use of the first or last recorded echocardiogram (for those with multiple echocardiograms) as the index echocardiogram to define AS severity impacted results, model 2 results were repeated using the first echocardiogram (results in **Table S16**).
5) Model 10 (both Australian and US cohorts) – Adjusted for known time in AS stage – In order to test whether adjustment for known time in AS stage (for those with multiple echocardiograms) impacted findings, model 5A and 5B (fully adjusted model) results were repeated adjusting for time in AS stage (results in **Tables S17 and S18**)
6) Model 11 (US cohort only) – Stratified by presence of coronary artery disease - includes AS stage, age, sex, race, left ventricular ejection fraction, and presence of left heart disease. Estimates are stratified by a history of coronary artery disease, defined as presence of ischemic heart disease, percutaneous coronary intervention, or coronary artery bypass grafting. A predetermined interaction for coronary artery disease status on the relationship of AS and all-cause mortality was evaluated (results in **Table S19**).
7) Model 12 (US cohort only) - Stratified by presence of heart failure - includes AS stage, age, sex, race, left ventricular ejection fraction, and presence of left heart disease. Estimates are stratified by a history of heart failure. A predetermined interaction for heart failure status on the relationship of AS and all-cause mortality was evaluated (results in **Table S20**).
8) Model 13 (both Australian and US cohorts) – Direct comparison of severe to moderate AS – includes all variables in fully adjusted models (Models 5A and 5B). Directly compares severe and moderate AS (as the reference group) (results in text).
