# Supplementary material for: Increasing risk of mortality across the spectrum of aortic stenosis is independent of comorbidity & treatment: An international, parallel cohort study of 248,464 patients
Source: PLoS One. 2022 Jul 11;17(7):e0268580. doi: 10.1371/journal.pone.0268580 (PMC9273084; doi:10.1371/journal.pone.0268580)
Supplement: S5 Table — Displayed is a comparison of baseline characteristics at the time of the last echocardiogram for individuals with nonmissing and missing aortic valve area measurements in the US cohort. Number of observations represents the number with nonmissing observations for each variable. All estimates are listed as means ± standard deviations unless otherwise indicated. Cell values < 11 are suppressed per Medicare Data Use policy. (PDF) [file pone.0268580.s009.pdf]

S5 Table. Comparison of Baseline Characteristics of Individuals in the US Cohort with Missing and Non-missing Aortic Valve Area Measurements

	Number of observations	Aortic Valve Area missing (N = 26,889)	Aortic Valve Area Non-missing (N = 3,976)
Age - years	30865	81.0 ± 8.4	76.0 ± 9.2
Female – number (%)	30865	14107 (52.5)	2009 (50.5)
Body mass index – kg/m^2	22669	27.5 ± 6.0	27.5 ± 6.1
Peak tricuspid regurgitant velocity – m/s	24778	2.9 ± 0.5	2.7 ± 0.5
Left heart disease – number (%)	30835	9371 (34.9)	1762 (44.3)
Left ventricular ejection fraction - %	30827	61.8 ± 16.5	60.9 ± 17.4
Glomerular filtration rate – mL/min	24110	149.8 ± 181.4	121.4 ± 157.8
Race – number (%)	30731		
White		21812 (81.1)	3527 (88.7)
Black		2572 (9.6)	228 (5.7)
Other		2388 (8.9)	204 (5.1)
Mitral valve intervention – number (%)	30865	422 (1.6)	51 (1.3)
Tricuspid valve intervention – number (%)	30865	34 (0.1)	< 11
Inpatient – number (%)	30865	10521 (39.1)	2177 (54.8)
Diabetes mellitus – number (%)	30865	7898 (29.4)	1355 (34.1)
Hypertension – number (%)	30865	16953 (63.1)	2905 (73.1)
Hyperlipidemia – number (%)	30865	12489 (46.5)	2189 (55.1)
Smoking – number (%)	30865	1354 (5.0)	162 (4.1)
Chronic obstructive pulmonary disease – number (%)	30865	4727 (17.6)	872 (21.9)
Chronic kidney disease – number (%)	30865	9218 (34.3)	1770 (44.5)
Ischemic heart disease – number (%)	30865	12697 (47.2)	2358 (59.3)
Peripheral arterial disease – number (%)	30865	218 (8.1)	504 (12.7)
Percutaneous Coronary Intervention – number (%)	30865	1273 (4.7)	183 (4.6)
Coronary artery bypass grafting – number (%)	30865	648 (2.4)	102 (2.6)
Pacer or Implantable Cardioverter Defibrillator – number (%)	30865	1625 (6.0)	321 (8.1)
Atrial fibrillation/flutter – number (%)	30865	6041 (22.5)	1149 (28.9)
Congestive Heart Failure – number (%)	30865	10521 (39.1)	2177 (54.8)
Stroke or Transient Ischemic Attack – number (%)	30865	3345 (12.4)	467 (11.8)
Dementia – number (%)	30865	4244 (15.8)	829 (20.9)
Anemia – number (%)	30865	10886 (40.5)	2064 (51.9)

Cancer – number (%)	30865	3864 (14.4)	585 (14.7)
Cholesterol medications – number (%)	30865	10697 (39.8)	1729 (43.5)
Antiplatelets – number (%)	30865	9696 (36.1)	1573 (39.6)
Anticoagulants – number (%)	30865	3418 (12.7)	572 (14.4)
Beta blockers – number (%)	30865	10936 (40.7)	1679 (42.2)
Renin, angiotensin, neprilysn inhibitors – number (%)	30865	8542 (31.8)	1366 (34.4)
Other hypertension medications – number (%)	30865	7327 (27.3)	1249 (31.4)
Diuretics – number (%)	30865	7716 (28.7)	1384 (34.8)
Anti-arrhythmic medications – number (%)	30865	10359 (38.5)	1659 (41.7)
Insulin – number (%)	30865	1771 (6.6)	274 (6.9)
Other diabetic medications – number (%)	30865	2891 (10.8)	462 (11.6)
Nitrates – number (%)	30865	2460 (9.2)	418 (10.5)
Digoxin/digitalis – number (%)	30865	584 (2.2)	128 (3.2)
Psychiatric medications – number (%)	30865	7058 (29.3)	1056 (26.6)
Anti-inflammatory medications – number (%)	30865	10330 (38.4)	1627 (40.9)
Other medications – number (%)	30865	7277 (27.1)	1174 (29.5)
Aortic Stenosis Stage – number (%)<br>None<br>Mild<br>Moderate<br>Severe	30865	25763 (95.8)<br>891 (3.3)<br>180 (0.7)<br>55 (0.2)	919 (23.1)<br>1549 (39.0)<br>1018 (25.6)<br>490 (12.3)
10-year mortality – number (%)	30865	11916 (44.3)	2329 (58.6)

Displayed is a comparison of baseline characteristics at the time of the last echocardiogram for individuals with nonmissing and missing aortic valve area measurements in the US cohort. Number of observations represents the number with nonmissing observations for each variable. All estimates are listed as means ± standard deviations unless otherwise indicated. Cell values < 11 are suppressed per Medicare Data Use policy.
