# Supplementary material for: Increasing risk of mortality across the spectrum of aortic stenosis is independent of comorbidity & treatment: An international, parallel cohort study of 248,464 patients
Source: PLoS One. 2022 Jul 11;17(7):e0268580. doi: 10.1371/journal.pone.0268580 (PMC9273084; doi:10.1371/journal.pone.0268580)
Supplement: S6 Table — Listed is the comparison of baseline characteristics at the time of an individual’s last known echocardiogram across aortic stenosis stages in the Australian cohort. Values are presented as means ± standard deviations unless otherwise indicated. AS = aortic stenosis, N = number. (PDF) [file pone.0268580.s010.pdf]

S6 Table. Baseline Characteristics of Included Patients (Australian Cohort)

**Baseline Characteristics (Australian Cohort)**					
	**Total (N = 217,599)**	**No AS (N = 173,776)**	**Mild AS (N = 27,921)**	**Moderate AS (N = 10,789)**	**Severe AS (N = 5,113)**
**Demographic profile**					
Age, years	76.0 ± 7.4	75.4 ± 7.1	78.0 ± 7.5	79.3 ± 7.5	80.4 ± 7.5
Female, %	107,312 (49.3%)	86,409 (49.7%)	13,800 (49.4%)	4737 (43.9%)	2366 (46.3%)
Race	N/A				
**Clinical Profile**					
Inpatient, %	N/A				
Body mass index, m/kg^2	27.6 ± 5.7	27.5 ± 5.7	28.1 ± 6.1	27.9 ± 5.7	26.8 ± 5.5
Systolic blood pressure, mmHg	140 ± 23.1	139 ± 22.9	142 ± 23.3	144 ± 24.0	141 ± 24.5
Diastolic blood pressure, mmHg	77.3 ± 11.3	77.4 ± 11.2	76.0 ± 11.9	77.8 ± 11.2	76.5 ± 11.6
Heart rate, beats per minute	72.2 ± 15.7	72.2 ± 15.8	71.6 ± 15.8	72.8 ± 15.4	73.4 ± 15.8
Estimated glomerular filtration rate, mL/min/1.73m^2	N/A				
NT-proBNP, pg/ml	N/A				
**Past Medical History**					
Specific past history	N/A				
Any left heart disease, %	76,014 (43.7%)	13,534 (48.5%)	6676 (61.9%)	3806 (74.4%)	3806 (74.4%)
**Aortic Valve Profile**					
Peak aortic velocity, m/s	1.7 ± 0.7	1.4 ± 0.3	2.4 ± 0.3	3.3± 0.3	4.5 ± 0.5
Mean aortic gradient, mmHg	9.6 ± 11.2	4.5 ± 1.8	13.5 ± 2.7	25.8 ± 5.6	49.7 ± 12.8
Aortic valve area, cm^2	2.3 ± 0.9	2.6 ± 0.8	1.7 ± 0.6	1.1 ± 0.4	0.8 ± 0.3
Aortic regurgitation, %	3981 (4.4%)	2,425 (3.1%)	889 (11.7%)	265 (15.2%)	251 (11.4%)
**Right Ventricular Function & Dimensions**					
Peak tricuspid regurgitant velocity, m/s	2.8 ± 0.5	2.7 ± 0.5	2.9 ± 0.5	3.0 ± 0.5	3.1 ± 0.6
Moderate or greater tricuspid regurgitation, %	19,826 (26.7%)	15,300 (26.2%)	2674 (29.0%)	1119 (28.7%)	563 (28.7%)
**Left Ventricular Function & Dimensions**					
Left atrial volume index, mL/m^2	37.0 (27.0, 58.0)	36.0 (27.0, 57.0)	38.0 (29.0, 52.0)	47.0 (33.0, 82.0)	48.0 (35.0, 77.3)
Left ventricular end-diastolic dimension, cm	4.7 ± 0.7	4.7 ± 0.8	4.6 ± 0.7	4.6 ± 0.8	4.6 ± 0.8
Left ventricular end-systolic dimension, cm	3.0 ± 0.8	3.0 ± 0.8	2.9 ± 0.8	2.9 ± 0.8	2.9 ± 0.8
Left ventricular ejection fraction, %	61.1 ± 13.9	60.7 ± 13.8	62.4 ± 13.4	63.2 ± 14.5	63.2 ± 14.3
Transmitral E/e' ratio	12.1 ± 5.4	11.7 ± 5.1	13.8 ± 6.3	14.5 ± 6.4	15.9 ± 7.5
Transmitral E/A ratio	1.0 ± 0.7	1.0 ± 0.7	1.0 ± 0.5	1.0 ± 0.8	1.0 ± 0.9
Stroke volume index, mL/m^2	40.8 ± 12.3	39.9 ± 11.7	44.9 ± 13.9	44.8 ± 13.9	43.9 ± 14.0
Moderate or greater mitral regurgitation, %	20,032 (14.9%)	15,497 (14.3%)	2605 (15.8%)	1246 (18.5%)	684 (21.1%)
**Pharmacotherapy**					
Specific treatments	N/A				

Listed is the comparison of baseline characteristics at the time of an individual's last known echocardiogram across aortic stenosis stages in the Australian cohort. Values are presented as means ± standard deviations unless otherwise indicated. AS = aortic stenosis, N = number.
