# Supplementary material for: Increasing risk of mortality across the spectrum of aortic stenosis is independent of comorbidity & treatment: An international, parallel cohort study of 248,464 patients
Source: PLoS One. 2022 Jul 11;17(7):e0268580. doi: 10.1371/journal.pone.0268580 (PMC9273084; doi:10.1371/journal.pone.0268580)
Supplement: S7 Table — Displayed are the univariate hazard ratios for all-cause mortality in the US and Australian cohorts according to AS stage. The US model included 30,865 patients with 14,481 deaths and 16,384 censored individuals. The Australian model includes 217,599 patients with 89,064 deaths and 128,535 censored patients. All comparisons are significant at a p < 0.001 level. (PDF) [file pone.0268580.s011.pdf]

S7 Table. Results of Model 1: Univariate Hazard Ratios for All-Cause Mortality

	US Cohort<br>14,481 deaths / 30,865 patients	Australian Cohort<br>89,064 deaths / 217,599 patients
**Covariates**	**Univariate Hazard Ratios (95% CI) for All-Cause Mortality**	
**AS Severity**		
**No AS**	***Reference Group***	***Reference Group***
**Mild AS**	**1.70** (1.61-1.79)	**1.46** (1.43-1.49)
**Moderate AS**	**2.24** (2.09-2.41)	**1.82** (1.77-1.87)
**Severe AS**	**2.17** (2.00-2.40)	**2.18** (2.10-2.25)

Displayed are the univariate hazard ratios for all-cause mortality in the US and Australian cohorts according to AS stage. The US model included 30,865 patients with 14,481 deaths and 16,384 censored individuals. The Australian model includes 217,599 patients with 89,064 deaths and 128,535 censored patients. All comparisons are significant at a $p < 0.001$ level.
