# Supplementary material for: Increasing risk of mortality across the spectrum of aortic stenosis is independent of comorbidity & treatment: An international, parallel cohort study of 248,464 patients
Source: PLoS One. 2022 Jul 11;17(7):e0268580. doi: 10.1371/journal.pone.0268580 (PMC9273084; doi:10.1371/journal.pone.0268580)
Supplement: S8 Table — Represents the univariate comparison of individuals who were alive/censored at 10-years after the last echocardiogram or dead in the Australian Cohort. P < 0.001 for all univariate comparisons. Time in AS stage also calculable in 56,026 cases. CI = confidence interval, LA = left atrial, LV = left ventricular, TR = tricuspid regurgitant. (PDF) [file pone.0268580.s012.pdf]

S8 Table. Univariate Comparisons of Individuals Dead vs. Alive/Censored at 10-Years in the Australian Cohort

**10-Year Mortality According to Baseline Characteristics (Australian Cohort)**			
	**Alive/Censored in 10 years (n=134,006)**	**All-Cause Mortality in 10 years (n=83,593)**	**Odds Ratio (95% CI) / Mean difference (95% CI)**
**Demographic profile (N=217,599)**			
Age, years	74.0 ± 6.6	79.2 ± 7.4	-5.2 (-5.3 to -5.2)
Female vs. Male, %	68,985 (64.3%) vs. 65,021 (59.0%)	38,327 (43.9%) vs. 45,266 (41.0%)	0.80 (0.78 to 0.81)
Race	N/A		
**Clinical Profile**			
Inpatient, %	N/A		
Body mass index, m/kg^2	28.2 ± 5.7 (96,190)	26.6 ± 5.8 (54,112)	+1.62 (+1.56 to +1.68)
Systolic blood pressure, mmHg	140.5 ± 22.2 (19,359)	139.0 ± 25.0 (8,506)	+1.54 (+0.95 to +2.13)
Diastolic blood pressure, mmHg	77.7 ± 11.0 (19,284)	76.4 ± 11.8 (8,474)	+1.35 (+1.06 to +1.64)
Heart rate, beats per minute	70.9 ± 15.1 (60,844)	74.7 ± 16.7 (32,745)	-3.79 (-4.01 to -3.58)
Estimated glomerular filtration rate, $mL/min/1.73m^2$	N/A		
NT-proBNP, pg/ml	N/A		
**Past Medical History**			
Specific past history	N/A		
Any left heart disease vs. none, %	55,782 (55.8%) vs. 78,224 (66.5%)	44,248 (44.2%) vs. 39,345 (33.5%)	1.58 (1.55 to 1.61)
**Aortic Valve Profile**			
Peak aortic velocity, m/s	1.7 ± 0.7 (131,303)	2.4 ± 0.3 (80,332)	-0.20 (-0.21 to -0.19)
Mean aortic gradient, mmHg	8.0 ± 9.2 (69,856)	12.0 ± 32.2 (48,277)	-4.03 (-4.16 to -3.59)
Aortic valve area, cm^2	2.4 ± 2.1 (64,106)	0.86 ± 0.91 (40,583)	+0.34 (+0.33 to +0.35)
Aortic regurgitation vs. none	4,192 (50.2%) vs. 63,504 (59.9%)	4,156 (49.8%) vs. 42,563 (40.1%)	1.48 (1.42 to 1.55)
**Right Heart Function & Dimensions**			
Peak tricuspid regurgitant velocity, m/s	2.6 ± 0.4 (83,797)	2.9 ± 0.5 (57,151)	-0.25 (-0.25 to -0.24)
Moderate tricuspid regurgitation vs. none, %	8,026 (40.5%) vs. 31,552 (58.1%)	11,800 (59.5%) vs. 22,747 (41.9%)	2.04 (1.97 to 2.11)
**Left Heart Function & Dimensions**			
Left atrial volume index, mL/m^2	44.2 ± 28.3 (53,906)	56.2 ± 37.9 (25,084)	-11.9 (-12.4 to -11.5)
Left ventricular end-diastolic dimension, cm	4.6 ± 0.7 (102,713)	4.7 ± 0.7 (57,369)	-0.07 (-0.08 to -0.06)
Left ventricular end-systolic dimension, cm	2.9 ± 0.7 (91,561)	3.1 ± 1.0 (50,301)	-0.20 (-0.21 to -0.19)

Left ventricular ejection fraction, %	62.7 ± 12.3 (113,463)	58.4 ± 15.8 (67,880)	+4.26 (+4.13 to +4.41)
Transmitral E/e’ ratio	11.3 ± 4.5 (59,550)	13.9 ± 6.6 (27,474)	-2.52 (-2.62 to -2.44)
Transmitral E/A ratio	1.0 ± 0.58 (100,232)	1.0 ± 0.78 (51,685)	-0.08 (-0.09 to -0.07)
Stroke volume index, mL/m^2	41.0 ± 11.8 (39,581)	40.3 ± 13.2 (20,265)	+0.72 (+0.51 to +0.93)
Moderate or greater mitral regurgitation, %	8,787 (43.9%) vs. 72,093 (62.9%)	11,245 (56.1%) vs. 42,448 (37.1%)	2.17 (2.11 to 2.24)

Represents the univariate comparison of individuals who were alive/censored at 10-years after the last echocardiogram or dead in the Australian Cohort. P < 0.001 for all univariate comparisons. Time in AS stage also calculable in 56,026 cases. CI = confidence interval, LA = left atrial, LV = left ventricular, TR = tricuspid regurgitant.
