# Supplementary material for: Increasing risk of mortality across the spectrum of aortic stenosis is independent of comorbidity & treatment: An international, parallel cohort study of 248,464 patients
Source: PLoS One. 2022 Jul 11;17(7):e0268580. doi: 10.1371/journal.pone.0268580 (PMC9273084; doi:10.1371/journal.pone.0268580)
Supplement: S9 Table — Represents the univariate comparison of individuals who were alive/censored at 10-years after the last echocardiogram or dead in the US Cohort. p < 0.001 for all comparisons except diuretic use (p = 0.004), AR severity (p = 0.11), and history of revascularization (p = 0.10). CI = confidence interval, N = number. (PDF) [file pone.0268580.s013.pdf]

S9 Table. Univariate Comparisons of Individuals Dead vs. Alive/Censored at 10-Years in the US Cohort

**10-Year Mortality According to Baseline Characteristics (US Cohort)**			
	**Alive/Censored in 10 years (N = 16,620)**	**All-Cause Mortality in 10 years (N = 14,245)**	**Odds Ratio (95% CI) / Mean difference (95% CI)**
**Demographic profile (N = 30,865)**			
Age, years	74.8 ± 7.4	80.4 ± 8.3	- 5.6 (-5.7 to -5.4)
Female vs. Male, %	8835 (53.2%) vs. 7785 (46.8%)	7281 (51.1%) vs. 6964 (48.9%)	0.92 (0.88 to 0.96)
White Race vs. Black/Other, %	12993 (78.8%)<br>3493 (21.2%)	12346 (86.7%)<br>1899 (13.3%)	1.75 (1.64 to 1.86)
**Past Medical History**			
Diabetes Mellitus, %	4058 (24.4%)	5195 (36.5%)	1.78 (1.69 to 1.87)
Hypertension, %	9465 (57.0%)	10393 (73.0%)	2.04 (1.94 to 2.14)
Ischemic heart disease, % /<br>Revascularized, %	6280 (37.8%)<br>1199 (7.2%)	8775 (61.6%)<br>959 (6.7%)	2.64 (2.52 to 2.77)<br>0.93 (0.85 to 1.01)
Heart failure, %	4417 (26.6%)	8281 (58.1%)	3.84 (3.66 to 4.02)
**Aortic Valve Profile**			
Peak aortic velocity, m/s	1.6 ± 0.6	1.8 ± 0.8	-0.2 (-0.2 to -0.2)
Mean aortic gradient, mmHg	18.1 ± 13.6	22.2 ± 15.3	-4.2 (-5.0 to -3.4)
Aortic valve area, cm^2	1.44 ± 0.65	1.18 ± 0.49	+0.27 (+0.23 to +0.30)
Moderate or greater aortic regurgitation, %	127 (0.8%)	149 (1.0%)	1.22 (0.96 to 1.55)
**Right Ventricular Function & Dimensions**			
Peak tricuspid regurgitant velocity, m/s	2.6 ± 0.4	2.9 ± 0.5	-0.3 (-0.3 to -0.3)
Moderate or greater tricuspid regurgitation, %	1240 (7.5%)	2804 (19.7%)	2.70 (2.50 to 2.92)
**Left Ventricular Function & Dimensions**			
Left atrial volume index, mL/m^2	29.6 ± 10.6	35.5 ± 13.0	-5.9 (-7.0 to -4.8)
Left ventricular end-diastolic dimension, cm	4.4 ± 0.7	4.5 ± 0.8	-0.1 (-0.1 to -0.1)
Left ventricular end-systolic dimension, cm	2.8 ± 0.7	2.9 ± 0.9	-0.1 (-0.1 to -0.1)
Left ventricular ejection fraction, %	63.7 ± 14.2	59.3 ± 18.8	+4.4 (+4.0 to +4.8)
Transmitral E/e’ ratio	11.0 ± 4.4	13.2 ± 6.0	-2.2 (-2.3 to -2.0)
Transmitral E/A ratio	0.99 ± 0.51	1.12 ± 0.73	-0.12 (-0.14 to -0.11)
Stroke volume index, mL/m^2	39.5 ± 11.1	38.3 ± 13.1	+1.3 (+0.8 to +1.8)
Moderate or greater mitral regurgitation, %	1103 (6.6%)	2240 (15.7%)	2.44 (2.26 to 2.64)
**Pharmacotherapy**			
Anticoagulant, %	2215 (13.3%)	1775 (12.5%)	0.93 (0.87 to 0.99)
Diuretic, %	5017 (30.2%)	4083 (28.7%)	0.93 (0.88 to 0.98)
Neurohormonal antagonist, %	6333 (38.1%)	3575 (25.1%)	0.54 (0.52 to 0.57)
Antiplatelet, %	6887 (41.4%)	4382 (30.8%)	0.63 (0.60 to 0.66)
Anti-arrhythmic, %	6762 (40.7%)	5256 (36.9%)	0.85 (0.81 to 0.89)
Beta-blocker, %	7168 (43.1%)	5447 (38.2%)	0.82 (0.78 to 0.85)

Represents the univariate comparison of individuals who were alive/censored at 10-years after the last echocardiogram or dead in the US Cohort. $p < 0.001$ for all comparisons except diuretic use ($p = 0.004$), AR severity ($p = 0.11$), and history of revascularization ($p = 0.10$). CI = confidence interval, N = number.
