# Supplementary material for: Increasing risk of mortality across the spectrum of aortic stenosis is independent of comorbidity & treatment: An international, parallel cohort study of 248,464 patients
Source: PLoS One. 2022 Jul 11;17(7):e0268580. doi: 10.1371/journal.pone.0268580 (PMC9273084; doi:10.1371/journal.pone.0268580)
Supplement: S10 Table — Displayed are the results of model 4 evaluating adjustment for aortic valve area as a continuous variable in the Australian cohort. The model is adjusted for age, sex, presence of left heart disease, left ventricular ejection fraction, AS severity, as well as aortic valve area as a continuous variable. The Australian model included 92,761 patients with complete profiling with 37,155 deaths and 55,606 censored individuals. In the Australian cohort, all comparisons are significant at a p < 0.001 level, except for aortic valve area (p = 0.001). AS = aortic stenosis, CI = confidence interval. (PDF) [file pone.0268580.s014.pdf]

S10 Table. Results of Model 4: Adjustment for Aortic Valve Area as a Continuous Variable in the Australian Cohort

Australian Cohort<br>37,155 deaths / 92,761 patients	
**Covariates**	**Adjusted Hazard Ratios (95% CI) for All-Cause Mortality**
**Age (per 1-year increase)**	**1.08** (1.08 to 1.08)
**Female**	**0.80** (0.78 to 0.82)
**Left heart disease**	**1.14** (1.12 to 1.17)
**Left ventricular ejection fraction (per 1-% increase)**	**0.98** (0.98 to 0.98)
**Aortic valve area (per 1-cm^2 increase)**	**0.91** (0.89 to 0.98)
Aortic Stenosis Stage/Severity	
**No AS**	***Reference Group***
**Mild AS**	**1.06** (1.02 to 1.09)
**Moderate AS**	**1.22** (1.16 to 1.27)
**Severe AS**	**1.36** (1.29 to 1.44)

Displayed are the results of model 4 evaluating adjustment for aortic valve area as a continuous variable in the Australian cohort. The model is adjusted for age, sex, presence of left heart disease, left ventricular ejection fraction, AS severity, as well as aortic valve area as a continuous variable. The Australian model included 92,761 patients with complete profiling with 37,155 deaths and 55,606 censored individuals. In the Australian cohort, all comparisons are significant at a $p < 0.001$ level, except for aortic valve area ($p = 0.001$). AS = aortic stenosis, CI = confidence interval.
