# Supplementary material for: Increasing risk of mortality across the spectrum of aortic stenosis is independent of comorbidity & treatment: An international, parallel cohort study of 248,464 patients
Source: PLoS One. 2022 Jul 11;17(7):e0268580. doi: 10.1371/journal.pone.0268580 (PMC9273084; doi:10.1371/journal.pone.0268580)
Supplement: S11 Table — Displayed are the results of model 5A which represents the fully adjusted model in the Australian cohort. This model is adjusted for age, sex, body mass index, presence of left heart disease, left ventricular ejection fraction, stroke volume index, time in stage, aortic valve area as a continuous measure, and AS severity. The model included 26,659 individuals with complete profiling with 9,633 deaths and 17,026 censored individuals. All comparisons are significant at a p < 0.001 level. Directly comparing severe to moderate AS with regard to risk of all-cause death (1,553 individuals included in model with 941 deaths and 612 censored individuals), the adjusted hazard ratio was 0.99 (95% CI 0.85 to 1.15), p = 0.88. TR = tricuspid regurgitant. (PDF) [file pone.0268580.s015.pdf]

S11 Table. Results of Model 5A: Fully Adjusted Model in the Australian Cohort

Australian Cohort<br>9,633 deaths /26,659 patients	
**Covariates**	**Adjusted Hazard Ratios (95% CI) for All-Cause Mortality**
**Age (per 1-year increase)**	**1.06** (1.06 to 1.07)
**Female**	**0.83** (0.79 to 0.87)
**Body mass index (per 1-kg/m^2 increase)**	**0.97** (0.97 to 0.97)
**Peak TR velocity (per 1-m/s increase)**	**1.99** (1.91 to 2.07)
**Left heart disease**	**1.14** (1.12 to 1.16)
**Left ventricular ejection fraction (per 1-% increase)**	**0.99** (0.98 to 0.99)
**Stroke volume index (per 1-mL/m^2 increase)**	**0.99** (0.99 to 0.99)
**Aortic valve area (per 1-cm^2 increase)**	**0.90** (0.88 to 0.94)
Aortic Stenosis Stage/Severity	
**No AS**	***Reference Group***
**Mild AS**	**1.36** (1.14 to 1.61)
**Moderate AS**	**2.06** (1.80 to 2.32)
**Severe AS**	**2.03** (1.78 to 2.32)

Displayed are the results of model 5A which represents the fully adjusted model in the Australian cohort. This model is adjusted for age, sex, body mass index, presence of left heart disease, left ventricular ejection fraction, stroke volume index, time in stage, aortic valve area as a continuous measure, and AS severity. The model included 26,659 individuals with complete profiling with 9,633 deaths and 17,026 censored individuals. All comparisons are significant at a $p < 0.001$ level. Directly comparing severe to moderate AS with regard to risk of all-cause death (1,553 individuals included in model with 941 deaths and 612 censored individuals), the adjusted hazard ratio was 0.99 (95% CI 0.85 to 1.15), $p = 0.88$. TR = tricuspid regurgitant.
