# Supplementary material for: Increasing risk of mortality across the spectrum of aortic stenosis is independent of comorbidity & treatment: An international, parallel cohort study of 248,464 patients
Source: PLoS One. 2022 Jul 11;17(7):e0268580. doi: 10.1371/journal.pone.0268580 (PMC9273084; doi:10.1371/journal.pone.0268580)
Supplement: S13 Table — Displayed are the results of model 6, the results of a sensitivity analysis using an aortic valve area classification scheme to reclassify individuals with velocities/gradients in the mild to moderate range who have an AVA < 1.0 cm2 as severe low gradient AS. These models are adjusted for age, sex, race (US cohort only), presence of left heart disease, left ventricular ejection fraction and AS severity. In the US cohort, there were 30,693 individuals with complete profiling with 14,451 deaths and 16,242 censored individuals. After full adjustment for all variables in model 5B (see S1 Raw images), there were 14,060 individuals with complete profiling of which 7,470 died and 6,590 were censored. Compared to no AS, the hazard ratio was 1.25 (95% CI 1.16–1.35) for mild AS, 1.33 (95% CI 1.16–1.52) for moderate AS, 1.45 (95% CI 1.25–1.67) for severe low-gradient AS, and 1.37 (95% CI 1.17–1.59) for severe high gradient AS. All comparisons in both models were significant at a p < 0.001 level. In the Australian cohort, there were 181,343 individuals with complete profiling with 72,928 deaths and 108,415 censored individuals. After full adjustment for age, sex, body mass index, left ventricular ejection fraction, presence of left heart disease, stroke volume index, and tricuspid regurgitant velocity (26,659 with complete profiling with 9,633 all-cause deaths and 17,026 censored individuals), compared to no AS, the hazard ratio was 1.39 (95% CI 1.29–1.49) for mild AS, 1.81 (95% CI 1.59–2.06) for moderate AS, 1.73 (95% CI 1.55–1.94) for severe low gradient AS, and 2.02 (95% CI 1.77–2.30) for severe high gradient AS. The hazard ratio for aortic valve area (for a 1-cm2 increase) in this fully adjusted model was 0.90 (95% CI 0.88–0.94). All comparisons in both models were significant at a p < 0.001 level. (PDF) [file pone.0268580.s017.pdf]

S13 Table. Results of Model 6: Sensitivity Analysis Using an Aortic Valve Area Classification Schema

	US Cohort 14,451 deaths / 30,693 patients	Australian Cohort 72,928 deaths / 181,343 patients
**Covariates**	**Adjusted Hazard Ratios (95% CI) for All-Cause Mortality**	
**Age (per 1-year increase)**	**1.06** (1.06 to 1.06)	**1.09** (1.09 to 1.09)
**Female**	**0.86** (0.83 to 0.89)	**0.79** (0.78 to 0.80)
**Race**		
**White**	*Reference Group*	**N/A**
**Black**	**1.07** (1.01 to 1.13)	
**Other**	**0.66** (0.61 to 0.71)	
**Left heart disease**	**1.24** (1.19 to 1.30)	**1.14** (1.12 to 1.15)
**Left ventricular ejection fraction (per 1-% increase)**	**0.99** (0.99 to 0.99)	**0.98** (0.98 to 0.98)
Aortic Stenosis Stage/severity		
**No AS**	***Reference Group***	***Reference Group***
**Mild AS**	**1.34** (1.27 to 1.42)	**1.23** (1.20 to 1.25)
**Moderate AS**	**1.61** (1.47 to 1.77)	**1.42** (1.37 to 1.47)
**Severe AS (Low Gradient)**	**1.73** (1.57 to 1.91)	**1.52** (1.47 to 1.58)
**Severe AS (High Gradient)**	**1.63** (1.47 to 1.80)	**1.67** (1.61 to 1.73)

Displayed are the results of model 6, the results of a sensitivity analysis using an aortic valve area classification scheme to reclassify individuals with velocities/gradients in the mild to moderate range who have an AVA < 1.0 cm^2 as severe low gradient AS. These models are adjusted for age, sex, race (US cohort only), presence of left heart disease, left ventricular ejection fraction and AS severity. In the US cohort, there were 30,693 individuals with complete profiling with 14,451 deaths and 16,242 censored individuals. After full adjustment for all variables in model 5B (see **Supplemental Appendix Table S12**), there were 14,060 individuals with complete profiling of which 7,470 died and 6,590 were censored. Compared to no AS, the hazard ratio was 1.25 (95% CI 1.16-1.35) for mild AS, 1.33 (95% CI 1.16-1.52) for moderate AS, 1.45 (95% CI 1.25-1.67) for severe low-gradient AS, and 1.37 (95% CI 1.17-1.59) for severe high gradient AS. All comparisons in both models were significant at a p < 0.001 level. In the Australian cohort, there were 181,343 individuals with complete profiling with 72,928 deaths and 108,415 censored individuals. After full adjustment for age, sex, body mass index, left ventricular ejection fraction, presence of left heart disease, stroke volume index, and tricuspid regurgitant velocity (26,659 with complete profiling with 9,633 all-cause deaths and 17,026 censored individuals), compared to no AS, the hazard ratio was 1.39 (95% CI 1.29-1.49) for mild AS, 1.81 (95% CI 1.59-2.06) for moderate AS, 1.73 (95% CI 1.55-1.94) for severe low gradient AS, and 2.02 (95% CI 1.77-2.30) for severe high gradient AS. The hazard ratio for aortic valve area (for a 1-cm^2 increase) in this fully adjusted model was 0.90 (95% CI 0.88-0.94). All comparisons in both models were significant at a p < 0.001 level.
