# Supplementary material for: Increasing risk of mortality across the spectrum of aortic stenosis is independent of comorbidity & treatment: An international, parallel cohort study of 248,464 patients
Source: PLoS One. 2022 Jul 11;17(7):e0268580. doi: 10.1371/journal.pone.0268580 (PMC9273084; doi:10.1371/journal.pone.0268580)
Supplement: S14 Table — Displayed are the results of model 7, the results of a sensitivity analysis amongst individuals < 65 at the time of echocardiography in the Australian cohort. A total of 235,562 individuals were < 65 years at the time of echocardiography (124,491 men [mean age 48.2 ± 12.6 years]; 111,071 females [mean age 46.7; mean age ± 13.0 years) of which 55,125 had complete profiling and were included in the model. This model is adjusted for age, sex, presence of left heart disease, left ventricular ejection fraction, and AS severity. Of the 55,125 individuals with complete profiling, there were 7,729 deaths and 47,396 censored individuals. All comparisons are significant at a p < 0.001 level except for females vs. males (p = 0.17). (PDF) [file pone.0268580.s018.pdf]

S14 Table. Results of Model 7: Sensitivity Analysis Reporting Results for Individuals Under Age 65 at the Time of Echocardiogram in the Australian Cohort

	Australian Cohort (Aged <65 years) 7,729 deaths / 55,125 patients
**Covariates**	**Adjusted Hazard Ratios (95% CI) for All-Cause Mortality**
**Age (per 1-year increase)**	**1.04** (1.03 to 1.04)
**Female**	**0.97** (0.92 to 1.01)
**Left heart disease**	**1.13** (1.11 to 1.14)
**Left ventricular ejection fraction (per 1-% increase)**	**0.97** (0.97 to 0.97)
Aortic Stenosis stage/severity	
**No AS**	***Reference Group***
**Mild AS**	**1.76** (1.62 to 1.91)
**Moderate AS**	**1.90** (1.66 to 2.17)
**Severe AS**	**2.19** (1.83 to 2.61)

Displayed are the results of model 7, the results of a sensitivity analysis amongst individuals < 65 at the time of echocardiography in the Australian cohort. A total of 235,562 individuals were < 65 years at the time of echocardiography (124,491 men [mean age 48.2 ± 12.6 years]; 111,071 females [mean age 46.7; mean age ± 13.0 years) of which 55,125 had complete profiling and were included in the model. This model is adjusted for age, sex, presence of left heart disease, left ventricular ejection fraction, and AS severity. Of the 55,125 individuals with complete profiling, there were 7,729 deaths and 47,396 censored individuals. All comparisons are significant at a p < 0.001 level except for females vs. males (p = 0.17).
