# Supplementary material for: Increasing risk of mortality across the spectrum of aortic stenosis is independent of comorbidity & treatment: An international, parallel cohort study of 248,464 patients
Source: PLoS One. 2022 Jul 11;17(7):e0268580. doi: 10.1371/journal.pone.0268580 (PMC9273084; doi:10.1371/journal.pone.0268580)
Supplement: S15 Table — Displayed are the results of model 9, results of a sensitivity analysis, using an individual’s first rather than last echocardiogram as the index to define AS severity stage. Models are adjusted for age, sex, race (US cohort only), presence of left heart disease, left ventricular ejection fraction and AS severity. In the US cohort, there were 29,930 individuals with complete profiling of which 14,373 died and 15,557 were censored. In the Australian cohort, there were 181,343 individuals with complete profiling of which 72,928 died and 108,415 were censored. All comparisons are significant at a p < 0.001 level. (PDF) [file pone.0268580.s019.pdf]

S15 Table. Results of Model 9: Sensitivity Analysis Reporting Results for the Relationship of AS Severity and All-Cause Mortality Using an Individual's First Echocardiogram as Index

	US Cohort 14,373 deaths / 29,930 patients	Australian Cohort 72,928 deaths / 181,343 patients
**Covariates**	**Adjusted Hazard Ratios (95% CI) for All-Cause Mortality**	
**Age (per 1-year increase)**	**1.06** (1.05 to 1.07)	**1.09** (1.09 to 1.09)
**Female**	**0.86** (0.84 to 0.89)	**0.79** (0.78 to 0.80)
**Race**		**0.79** (0.78 to 0.80)
**White**	*Reference Group*	
**Black**	**1.01** (0.95 to 1.07)	**N/A**
**Other**	**0.65** (0.60 to 0.68)	
**Left heart disease**	**1.19** (1.13 to 1.24)	**1.13** (1.11 to 1.14)
**Left ventricular ejection fraction (per 1-% increase)**	**0.99** (0.99 to 0.99)	**0.98** (0.98 to 0.98)
Aortic Stenosis stage/severity		
**No AS**	*Reference Group*	*Reference Group*
**Mild AS**	**1.29** (1.22 to 1.37)	**1.29** (1.26 to 1.32)
**Moderate AS**	**1.52** (1.41 to 1.63)	**1.65** (1.60 to 1.70)
**Severe AS**	**1.50** (1.36 to 1.66)	**1.92** (1.84 to 2.01)

Displayed are the results of model 9, results of a sensitivity analysis, using an individual's first rather than last echocardiogram as the index to define AS severity stage. Models are adjusted for age, sex, race (US cohort only), presence of left heart disease, left ventricular ejection fraction and AS severity. In the US cohort, there were 29,930 individuals with complete profiling of which 14,373 died and 15,557 were censored. In the Australian cohort, there were 181,343 individuals with complete profiling of which 72,928 died and 108,415 were censored. All comparisons are significant at a $p < 0.001$ level.
