# Supplementary material for: Increasing risk of mortality across the spectrum of aortic stenosis is independent of comorbidity & treatment: An international, parallel cohort study of 248,464 patients
Source: PLoS One. 2022 Jul 11;17(7):e0268580. doi: 10.1371/journal.pone.0268580 (PMC9273084; doi:10.1371/journal.pone.0268580)
Supplement: S16 Table — Displayed are the results of model 10, results of a sensitivity analysis evaluating the impact of adjustment for known time in AS stage in the US cohort. Both models are adjusted for age, sex, race, body mass index, peak tricuspid regurgitant velocity, presence of left heart disease, left ventricular ejection fraction, AS severity, estimated glomerular filtration rate, history of mitral or tricuspid valve interventions, inpatient status, diabetes, hypertension, hyperlipidemia, smoking, chronic obstructive pulmonary disease, chronic kidney disease, ischemic heart disease, peripheral arterial disease, history of percutaneous coronary intervention or coronary artery bypass grafting, pacemaker or implantable defibrillator, atrial fibrillation or flutter, heart failure, stroke or transient ischemic attack, dementia, anemia, cancer, cholesterol medications, antiplatelet medications, anticoagulants, beta blockers, renin-angiotensin-neprilysn inhibitors, other antihypertensives, diuretics, anti-arrhythmic medications, insulins, other diabetic medications, nitrates, digoxin, psychiatric medications, anti-inflammatory use, and other medications. Both models included 14,060 individuals with complete profiling with 7,470 deaths and 6,590 censored individuals. All comparisons are significant at a p < 0.001 level except for sex (p = 0.005), left ventricular ejection fraction (p = 0.02), estimated glomerular filtration rate (p = 0.40), black race (p = 0.11), mitral valve intervention (p = 0.25), tricuspid valve intervention (p = 0.58), ischemic heart disease (p = 0.10), pacer or implantable defibrillator (p = 0.001), atrial fibrillation/flutter (p = 0.23), cholesterol medication (p = 0.002), antiplatelet medications (p = 0.43), anticoagulants (p = 0.56), beta blockers (p = 0.46), other anti-hypertensive medications (p = 0.04), diuretics (p = 0.02), anti-arrhythmic medications (p = 0.02), other anti-diabetic medications (p = 0.36), nitrates (p = 0.20), digoxin/digitalis (p = 0.21), [file pone.0268580.s020.pdf]

S16 Table. Results of Model 10: Sensitivity Analysis Reporting Results for the Relationship of AS Severity and All-Cause Mortality with and Without Adjustment for Known Time in AS Stage in the US Cohort

US Cohort 7,470 deaths /14,060 patients		
	**Adjusted for Time in AS Stage**	**No Adjustment for Time in AS Stage**
**Covariates**	**Adjusted Hazard Ratio (95% CI) for All-Cause Mortality**	
**Age (per 1-year increase)**	**1.03** (1.03 to 1.04)	**1.03** (1.03 to 1.04)
**Female**	**0.89** (0.82 to 0.96)	**0.89** (0.82 to 0.96)
**Body mass index (per 1-kg/m^2 increase)**	**0.99** (0.98 to 0.99)	**0.99** (0.98 to 0.99)
**Peak TR velocity (per 1-m/s increase)**	**1.54** (1.46 to 1.61)	**1.54** (1.46 to 1.61)
**Left heart disease**	**1.14** (1.07 to 1.27)	**1.14** (1.07 to 1.27)
**Left ventricular ejection fraction (per 1-% increase)**	**0.99** (0.99 to 0.99)	**0.99** (0.99 to 0.99)
**Estimated glomerular filtration rate (per 1-mL/min increase)**	**0.99** (0.99 to 1.00)	**0.99** (0.99 to 1.00)
**Race**		
**White**	*Reference Group*	*Reference Group*
**Black**	**0.93** (0.85 to 1.02)	**0.93** (0.85 to 1.02)
**Other**	**0.75** (0.67 to 0.83)	**0.75** (0.67 to 0.83)
**Mitral valve intervention**	**1.14** (0.91 to 1.43)	**1.14** (0.91 to 1.43)
**Tricuspid valve intervention**	**0.78** (0.31 to 1.92)	**0.78** (0.31 to 1.92)
**Inpatient status (vs. outpatient)**	**1.53** (1.43 to 1.63)	**1.53** (1.43 to 1.63)
**Diabetes mellitus**	**1.12** (1.06 to 1.19)	**1.12** (1.06 to 1.19)
**Hypertension**	**0.80** (0.75 to 0.86)	**0.80** (0.75 to 0.86)
**Hyperlipidemia**	**0.74** (0.70 to 0.78)	**0.74** (0.70 to 0.78)
**Smoking history**	**1.24** (1.11 to 1.39)	**1.24** (1.11 to 1.39)
**Chronic obstructive pulmonary disease**	**1.37** (1.29 to 1.45)	**1.37** (1.29 to 1.45)
**Chronic kidney disease**	**1.46** (1.38 to 1.55)	**1.46** (1.38 to 1.55)
**Ischemic heart disease**	**0.95** (0.89 to 1.01)	**0.95** (0.89 to 1.01)
**Peripheral arterial disease**	**1.22** (1.13 to 1.32)	**1.22** (1.13 to 1.32)
**Percutaneous Coronary Intervention**	**0.73** (0.66 to 0.82)	**0.73** (0.66 to 0.82)
**Coronary Artery Bypass Grafting**	**0.43** (0.35 to 0.52)	**0.43** (0.35 to 0.52)
**Pacer or implantable defibrillator**	**1.16** (1.06 to 1.27)	**1.16** (1.06 to 1.27)
**Atrial fibrillation/flutter**	**0.97** (0.91 to 1.02)	**0.97** (0.91 to 1.02)
**Heart Failure**	**1.31** (1.23 to 1.39)	**1.31** (1.23 to 1.39)
**Stroke or transient ischemic attack**	**1.13** (1.06 to 1.21)	**1.13** (1.06 to 1.21)
**Dementia**	**1.26** (1.19 to 1.33)	**1.26** (1.19 to 1.33)
**Anemia**	**1.29** (1.22 to 1.36)	**1.29** (1.22 to 1.36)
**Cancer**	**1.36** (1.28 to 1.44)	**1.36** (1.28 to 1.44)
**Cholesterol medications**	**0.89** (0.83 to 0.96)	**0.89** (0.83 to 0.96)
**Antiplatelet medications**	**0.95** (0.83 to 1.08)	**0.95** (0.83 to 1.08)
**Anticoagulants**	**0.97** (0.88 to 1.07)	**0.97** (0.88 to 1.07)
**Beta blockers**	**0.96** (0.86 to 1.07)	**0.96** (0.86 to 1.07)

**Renin-angiotensin-neprilysin inhibitors**	**0.84** (0.78 to 0.90)	**0.84** (0.78 to 0.90)
**Other anti-hypertensive medications**	**0.93** (0.86 to 0.99)	**0.93** (0.86 to 0.99)
**Diuretics**	**1.09** (1.02 to 1.17)	**1.09** (1.02 to 1.17)
**Anti-arrhythmic medications**	**1.13** (1.02 to 1.26)	**1.13** (1.02 to 1.26)
**Insulins**	**1.28** (1.14 to 1.44)	**1.28** (1.14 to 1.44)
**Other anti-diabetic medications**	**1.05** (0.95 to 1.16)	**1.05** (0.95 to 1.16)
**Nitrates**	**1.06** (0.97 to 1.17)	**1.06** (0.97 to 1.17)
**Digoxin/digitalis**	**1.11** (0.95 to 1.29)	**1.11** (0.95 to 1.29)
**Psychiatric treatments**	**1.19** (1.11 to 1.27)	**1.19** (1.11 to 1.27)
**Anti-inflammatories**	**0.94** (0.83 to 1.07)	**0.94** (0.83 to 1.07)
**Other medications**	**1.08** (1.01 to 1.15)	**1.08** (1.01 to 1.15)
Aortic Stenosis Stage/Severity		
**No AS**	***Reference Group***	***Reference Group***
**Mild AS**	**1.26** (1.16 to 1.36)	**1.26** (1.16 to 1.36)
**Moderate AS**	**1.38** (1.24 to 1.53)	**1.38** (1.24 to 1.53)
**Severe AS**	**1.36** (1.17 to 1.59)	**1.36** (1.17 to 1.59)

Displayed are the results of model 10, results of a sensitivity analysis evaluating the impact of adjustment for known time in AS stage in the US cohort. Both models are adjusted for age, sex, race, body mass index, peak tricuspid regurgitant velocity, presence of left heart disease, left ventricular ejection fraction, AS severity, estimated glomerular filtration rate, history of mitral or tricuspid valve interventions, inpatient status, diabetes, hypertension, hyperlipidemia, smoking, chronic obstructive pulmonary disease, chronic kidney disease, ischemic heart disease, peripheral arterial disease, history of percutaneous coronary intervention or coronary artery bypass grafting, pacemaker or implantable defibrillator, atrial fibrillation or flutter, heart failure, stroke or transient ischemic attack, dementia, anemia, cancer, cholesterol medications, antiplatelet medications, anticoagulants, beta blockers, renin-angiotensin-neprilysn inhibitors, other antihypertensives, diuretics, anti-arrhythmic medications, insulins, other diabetic medications, nitrates, digoxin, psychiatric medications, anti-inflammatory use, and other medications. Both models included 14,060 individuals with complete profiling with 7,470 deaths and 6,590 censored individuals. All comparisons are significant at a $p < 0.001$ level except for sex ($p = 0.005$), left ventricular ejection fraction ($p = 0.02$), estimated glomerular filtration rate ($p = 0.40$), black race ($p = 0.11$), mitral valve intervention ($p = 0.25$), tricuspid valve intervention ($p = 0.58$), ischemic heart disease ($p = 0.10$), pacer or implantable defibrillator ($p = 0.001$), atrial fibrillation/flutter ($p = 0.23$), cholesterol medication ($p = 0.002$), antiplatelet medications ($p = 0.43$), anticoagulants ($p = 0.56$), beta blockers ($p = 0.46$), other anti-hypertensive medications ($p = 0.04$), diuretics ($p = 0.02$), anti-arrhythmic medications ($p = 0.02$), other anti-diabetic medications ($p = 0.36$), nitrates ($p = 0.20$), digoxin/digitalis ($p = 0.21$), anti-inflammatories ($p = 0.35$), and other medications ($p = 0.03$). TR = tricuspid regurgitant.
