# Supplementary material for: Increasing risk of mortality across the spectrum of aortic stenosis is independent of comorbidity & treatment: An international, parallel cohort study of 248,464 patients
Source: PLoS One. 2022 Jul 11;17(7):e0268580. doi: 10.1371/journal.pone.0268580 (PMC9273084; doi:10.1371/journal.pone.0268580)
Supplement: S17 Table — Displayed are the results of model 10, results of a sensitivity analysis evaluating the impact of adjustment for known time in AS stage in the Australian cohort. Models are adjusted for age, sex, body mass index, peak tricuspid regurgitant velocity, presence of left heart disease, left ventricular ejection fraction, aortic valve area, stroke volume index, and AS severity. Both models included 26,633 individuals with 9,424 deaths and 17,209 censored individuals. All comparisons are significant at a p < 0.001 level. TR = tricuspid regurgitant. (PDF) [file pone.0268580.s021.pdf]

S17 Table. Results of Model 10: Sensitivity Analysis Reporting Results for the Relationship of AS Severity and All-Cause Mortality with and Without Adjustment for Known Time in AS Stage in the Australian Cohort

Australian Cohort 9,424 deaths / 26,633 patients		
	**Adjusted for time in AS stage**	**No adjustment for time in AS stage**
**Covariates**	**Adjusted Hazard Ratios (95% CI) for All-Cause Mortality**	
**Age (per 1-year increase)**	**1.06** (1.06 to 1.07)	**1.06** (1.06 to 1.07)
**Female**	**0.83** (0.79 to 0.87)	**0.83** (0.79 to 0.87)
**Body mass index (per 1-kg/m^2 increase)**	**0.97** (0.97 to 0.97)	**0.97** (0.97 to 0.97)
**TR peak velocity (per 1-m/s increase)**	**2.01** (1.91 to 2.09)	**2.01** (1.92 to 2.09)
**Left heart disease**	**1.11** (1.06 to 1.16)	**1.11** (1.06 to 1.16)
**Left ventricular ejection fraction (per 1-% increase)**	**0.99** (0.98 to 0.99)	**0.99** (0.98 to 0.99)
**Stroke volume index (per 1-mL/m^2 increase)**	**0.99** (0.98 to 0.99)	**0.99** (0.98 to 0.99)
**Aortic valve area (per 1-cm^2 increase)**	**0.91 (**0.86 to 0.94)	**0.90 (**0.86 to 0.89)
Aortic Stenosis stage/severity		
**No AS**	***Reference Group***	***Reference Group***
**Mild AS**	**1.37** (1.15 to 1.63)	**1.41** (1.31 to 1.51)
**Moderate AS**	**2.07** (1.81 to 2.34)	**1.97** (1.77 to 2.18)
**Severe AS**	**2.05** (1.80 to 2.34)	**2.07** (1.82 to 2.36)

Displayed are the results of model 10, results of a sensitivity analysis evaluating the impact of adjustment for known time in AS stage in the Australian cohort. Models are adjusted for age, sex, body mass index, peak tricuspid regurgitant velocity, presence of left heart disease, left ventricular ejection fraction, aortic valve area, stroke volume index, and AS severity. Both models included 26,633 individuals with 9,424 deaths and 17,209 censored individuals. All comparisons are significant at a $p < 0.001$ level. TR = tricuspid regurgitant.
