# Supplementary material for: Increasing risk of mortality across the spectrum of aortic stenosis is independent of comorbidity & treatment: An international, parallel cohort study of 248,464 patients
Source: PLoS One. 2022 Jul 11;17(7):e0268580. doi: 10.1371/journal.pone.0268580 (PMC9273084; doi:10.1371/journal.pone.0268580)
Supplement: S18 Table — Displayed are the results of model 11, results of a sensitivity analysis stratifying by presence or absence of coronary artery disease (defined as ischemic heart disease or history of percutaneous coronary intervention or coronary artery bypass grafting) in the US cohort. The models are adjusted for age, sex, race, presence of left heart disease, left ventricular ejection fraction and AS severity. Of the 15,272 individuals with coronary artery disease and complete profiling, 8,940 died and 6,332 were censored. Of 15,421 individuals with complete profiling but without coronary artery disease, 5,511 died and 9,910 were censored. The p-value for interaction between presence of coronary artery disease and AS severity = 0.007. All comparisons significant at a p < 0.001 level. (PDF) [file pone.0268580.s022.pdf]

S18 Table. Results of a Model 11: Sensitivity Analysis Stratifying by Presence or Absence of Coronary Artery Disease in the US Cohort

	Coronary Artery Disease 8,940 deaths / 15,272 patients	No Coronary Artery Disease 5,511 deaths / 15,421 patients
**Covariates**	**Adjusted Hazard Ratio (95% CI) for All-Cause Mortality**	
**Age (per 1-year increase)**	**1.05** (1.05 to 1.05)	**1.07** (1.06 to 1.07)
**Female**	**0.94** (0.90 to 0.98)	**0.81** (0.76 to 0.85)
**Race**		
**White**	***Reference Group***	***Reference Group***
**Black**	**1.22** (1.13 to 1.33)	**0.96** (0.88 to 1.06)
**Other**	**0.78** (0.71 to 0.87)	**0.61** (0.55 to 0.68)
**Left heart disease**	**1.08** (1.02 to 1.59)	**1.35** (1.26 to 1.45)
**Left ventricular ejection fraction (per 1-% increase)**	**0.99** (0.99 to 0.99)	**0.99** (0.99-0.99)
Aortic Stenosis stage/severity		
**No AS**	***Reference Group***	***Reference Group***
**Mild AS**	**1.35** (1.26 to 1.45)	**1.31** (1.19 to 1.44)
**Moderate AS**	**1.61** (1.48 to 1.75)	**1.68** (1.47 to 1.93)
**Severe AS**	**1.50** (1.33 to 1.68)	**1.84** (1.51 to 2.24)

Displayed are the results of model 11, results of a sensitivity analysis stratifying by presence or absence of coronary artery disease (defined as ischemic heart disease or history of percutaneous coronary intervention or coronary artery bypass grafting) in the US cohort. The models are adjusted for age, sex, race, presence of left heart disease, left ventricular ejection fraction and AS severity. Of the 15,272 individuals with coronary artery disease and complete profiling, 8,940 died and 6,332 were censored. Of 15,421 individuals with complete profiling but without coronary artery disease, 5,511 died and 9,910 were censored. The p-value for interaction between presence of coronary artery disease and AS severity = 0.007. All comparisons significant at a $p < 0.001$ level.
