# Supplementary material for: Increasing risk of mortality across the spectrum of aortic stenosis is independent of comorbidity & treatment: An international, parallel cohort study of 248,464 patients
Source: PLoS One. 2022 Jul 11;17(7):e0268580. doi: 10.1371/journal.pone.0268580 (PMC9273084; doi:10.1371/journal.pone.0268580)
Supplement: S19 Table — Displayed are the results of model 12, a sensitivity analysis stratifying by presence or absence of precedent heart failure in the US cohort. Models are adjusted for age, sex, race, presence of left heart disease, left ventricular ejection fraction and AS severity. Of the 12,668 individuals with complete profiling and precedent heart failure, 8,336 died and 4,332 were censored. All comparisons are significant at a p < 0.001 level except for left heart disease (p = 0.65). Of the 18,025 individuals with complete profiling but absence of precedent heart failure, 6,115 died and 11,910 were censored. All comparisons were significant at a p < 0.001 level except for black race (p = 0.94). The p-value for interaction between presence of heart failure and AS severity = 0.0014. (PDF) [file pone.0268580.s023.pdf]

**S19 Table. Results of a Model 12: Sensitivity Analysis Stratifying by Presence or Absence of Heart Failure in the US Cohort**

| | Heart Failure 8,336 deaths / 12,668 patients | No Heart Failure 6,115 deaths / 18,025 patients |
|---|---|---|
| **Covariates** | **Adjusted Hazard Ratio (95% CI) for All-Cause Mortality** | |
| **Age (per 1-year increase)** | **1.04** (1.03 to 1.04) | **1.07** (1.06 to 1.07) |
| **Female** | **0.88** (0.84 to 0.92) | **0.82** (0.78 to 0.86) |
| **Race** | | |
| **White** | *Reference Group* | *Reference Group* |
| **Black** | **1.15** (1.06 to 1.24) | **1.00** (0.91 to 1.09) |
| **Other** | **0.82** (0.73 to 0.90) | **0.62** (0.56 to 0.69) |
| **Left heart disease** | **0.99** (0.93 to 1.05) | **1.29** (1.21 to 1.38) |
| **Left ventricular ejection fraction (per 1-% increase)** | **0.99** (0.99 to 0.99) | **0.99** (0.99 to 0.99) |
| ***Aortic Stenosis stage/severity*** | | |
| **No AS** | *Reference Group* | *Reference Group* |
| **Mild AS** | **1.31** (1.22 to 1.41) | **1.30** (1.18 to 1.42) |
| **Moderate AS** | **1.49** (1.37 to 1.62) | **1.65** (1.44 to 1.89) |
| **Severe AS** | **1.44** (1.27 to 1.63) | **1.68** (1.40 to 2.02) |

Displayed are the results of model 12, a sensitivity analysis stratifying by presence or absence of precedent heart failure in the US cohort. Models are adjusted for age, sex, race, presence of left heart disease, left ventricular ejection fraction and AS severity. Of the 12,668 individuals with complete profiling and precedent heart failure, 8,336 died and 4,332 were censored. All comparisons are significant at a $p < 0.001$ level except for left heart disease ($p = 0.65$). Of the 18,025 individuals with complete profiling but absence of precedent heart failure, 6,115 died and 11,910 were censored. All comparisons were significant at a $p < 0.001$ level except for black race ($p = 0.94$). The p-value for interaction between presence of heart failure and AS severity = 0.0014.
