# Supplementary figures and images for: Increasing risk of mortality across the spectrum of aortic stenosis is independent of comorbidity & treatment: An international, parallel cohort study of 248,464 patients
Source: PLoS One. 2022 Jul 11;17(7):e0268580. doi: 10.1371/journal.pone.0268580 (PMC9273084; doi:10.1371/journal.pone.0268580)

Raw images in Fig 3.

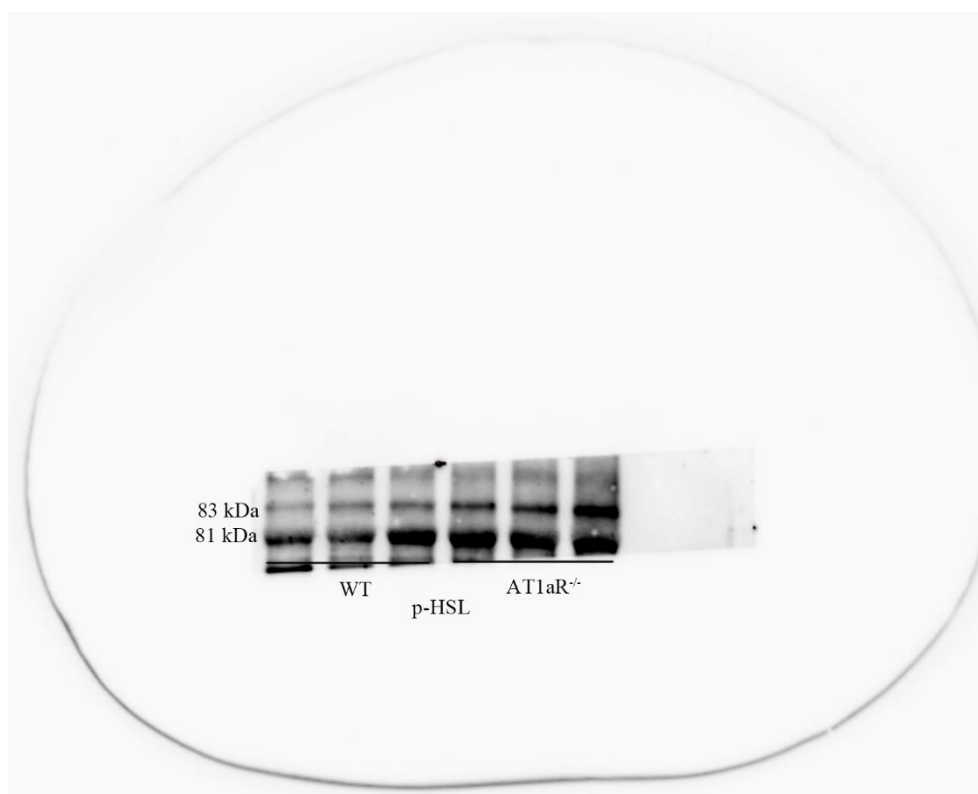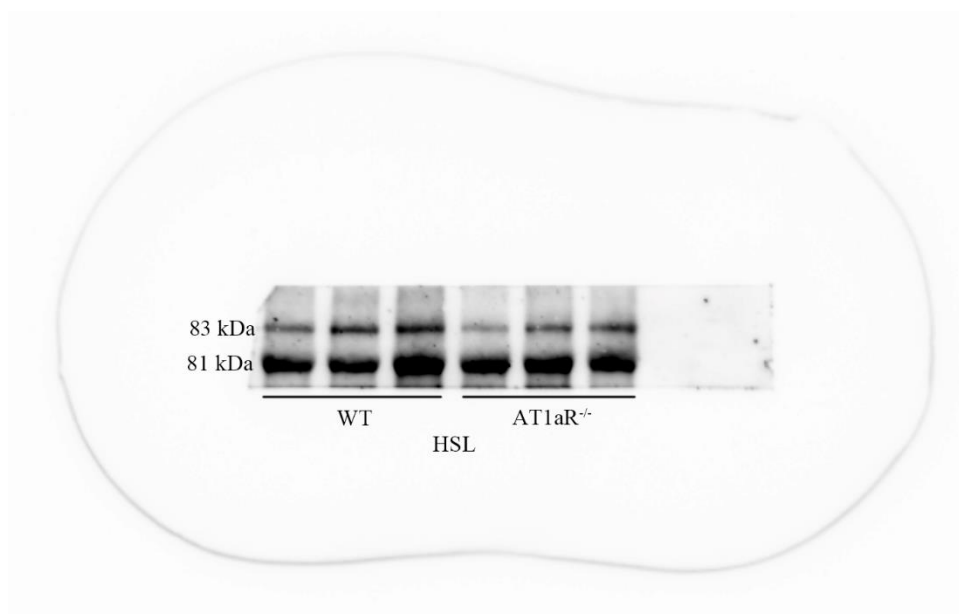

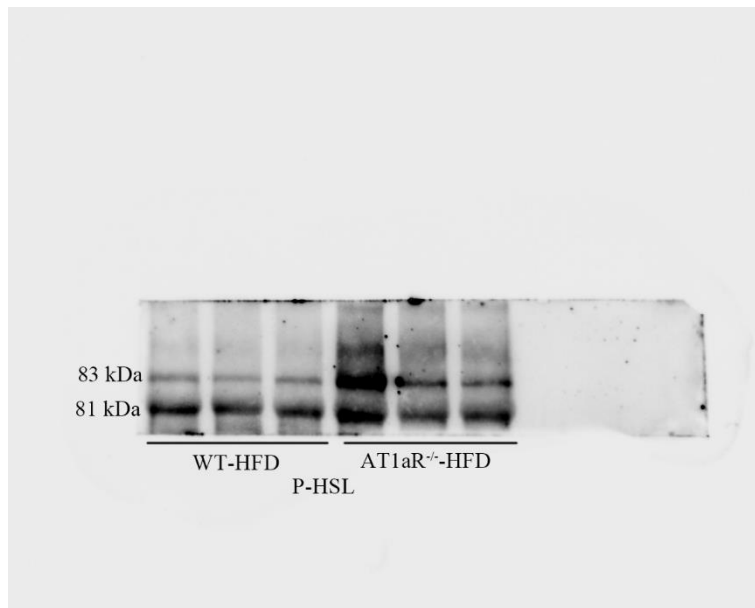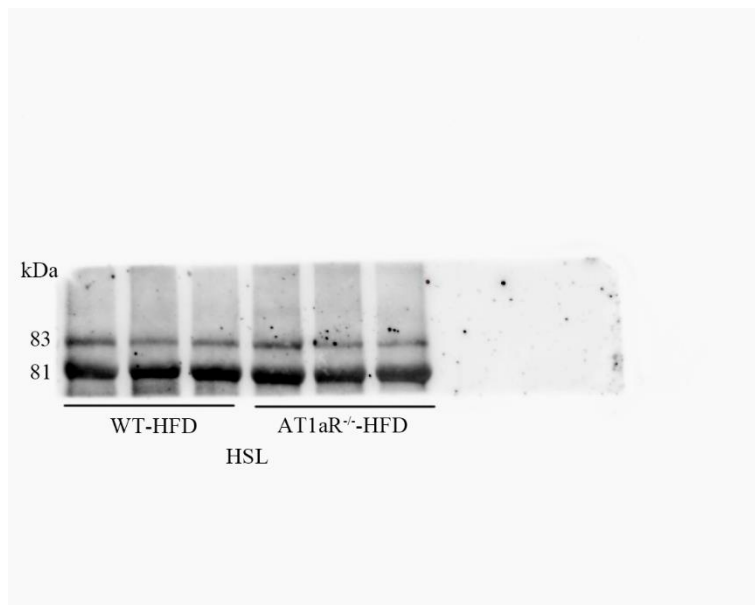

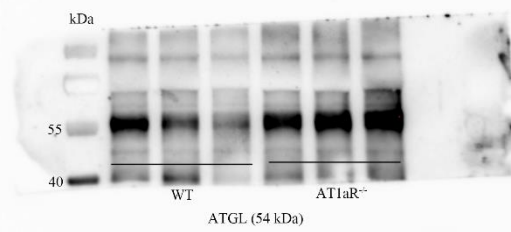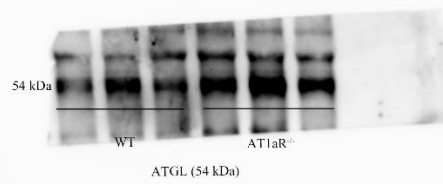

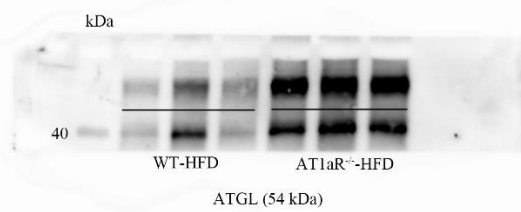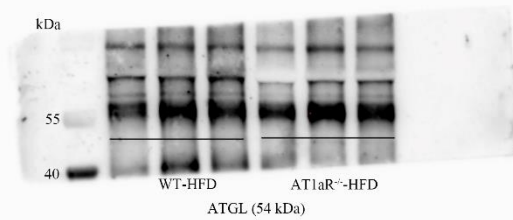

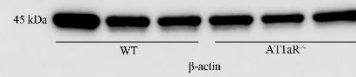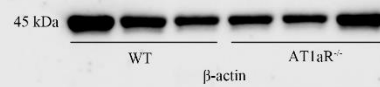

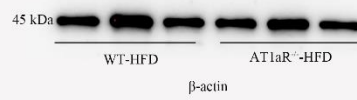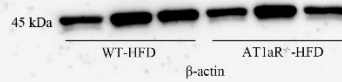

Raw images in Fig 5.

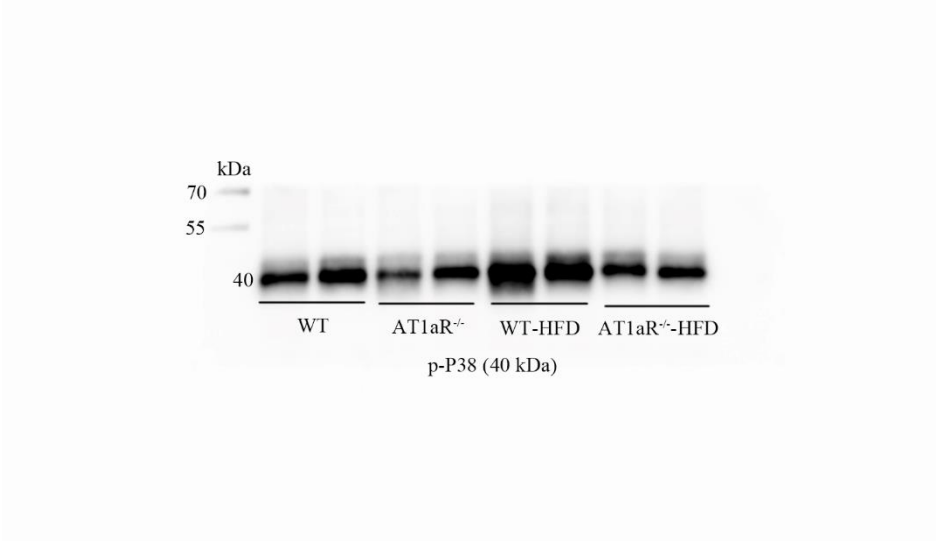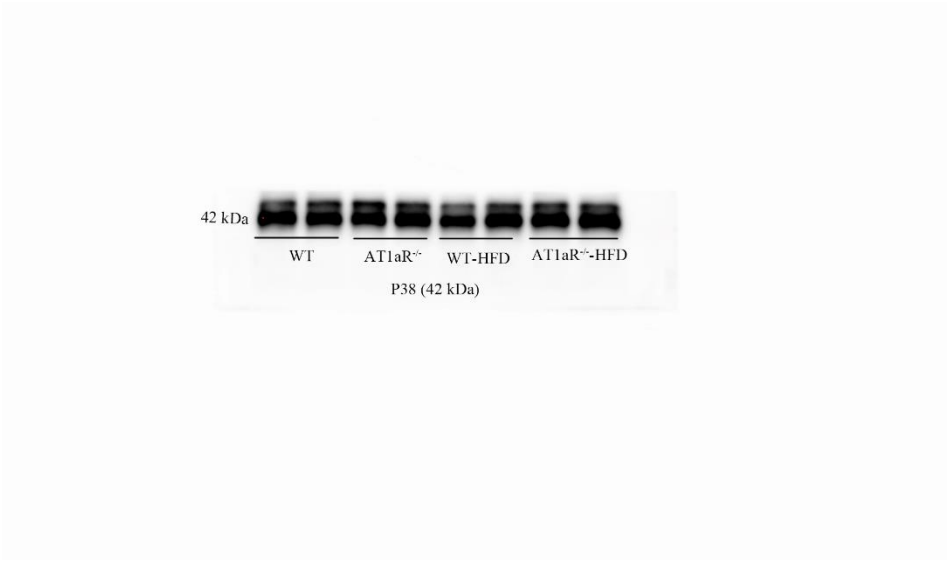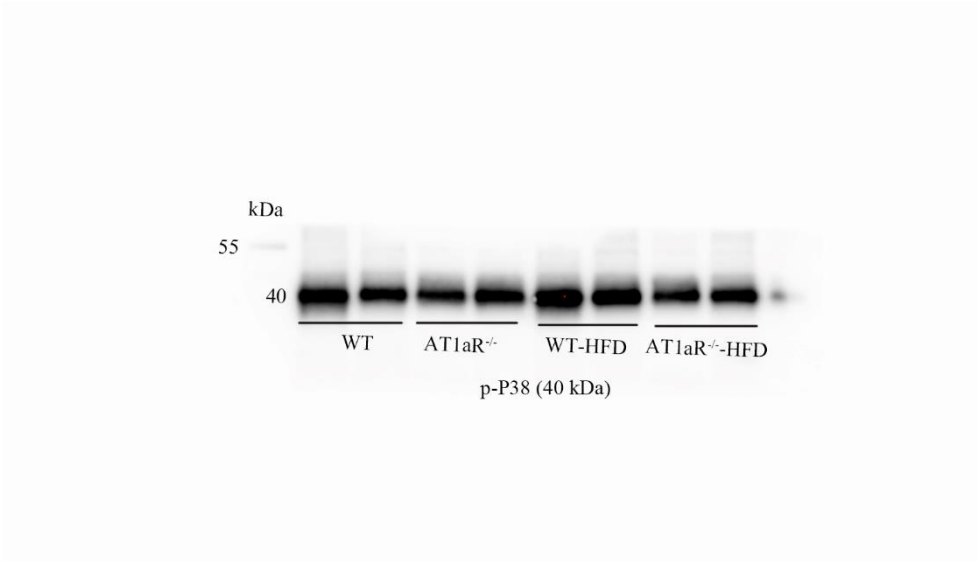

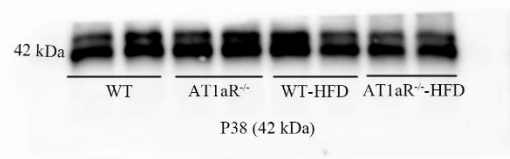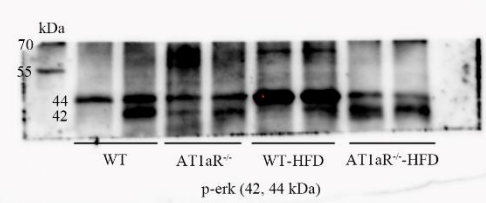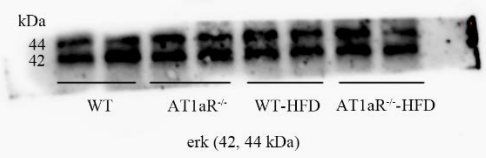

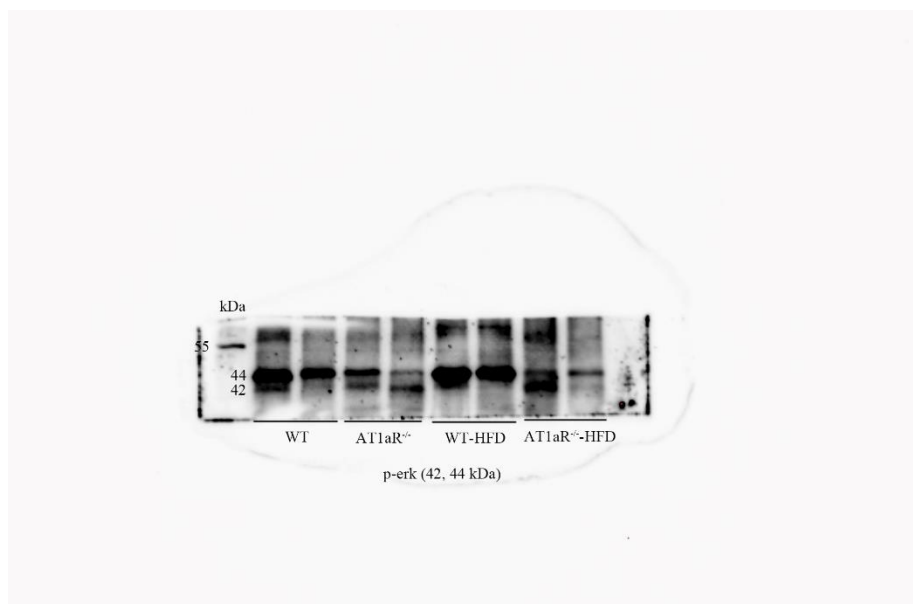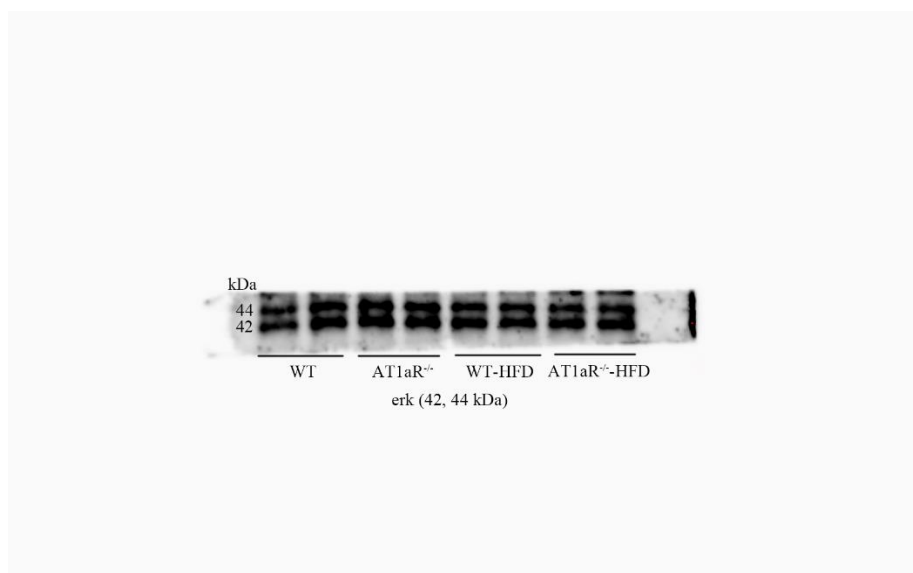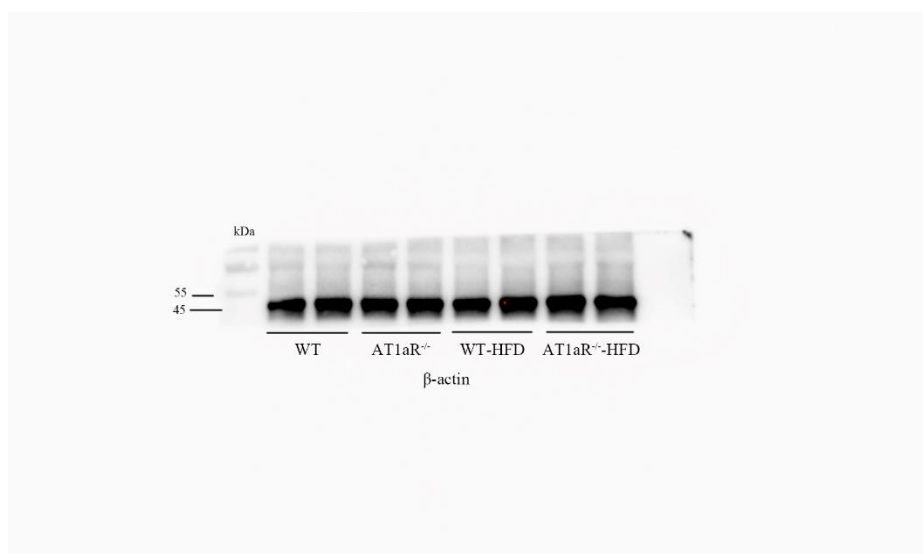

Raw images in Fig 6

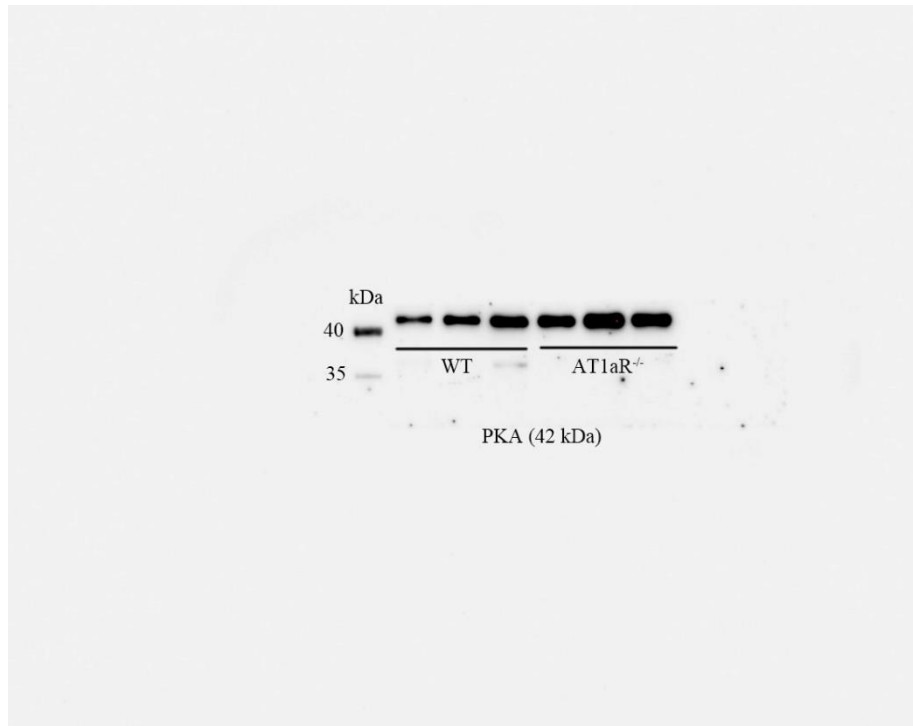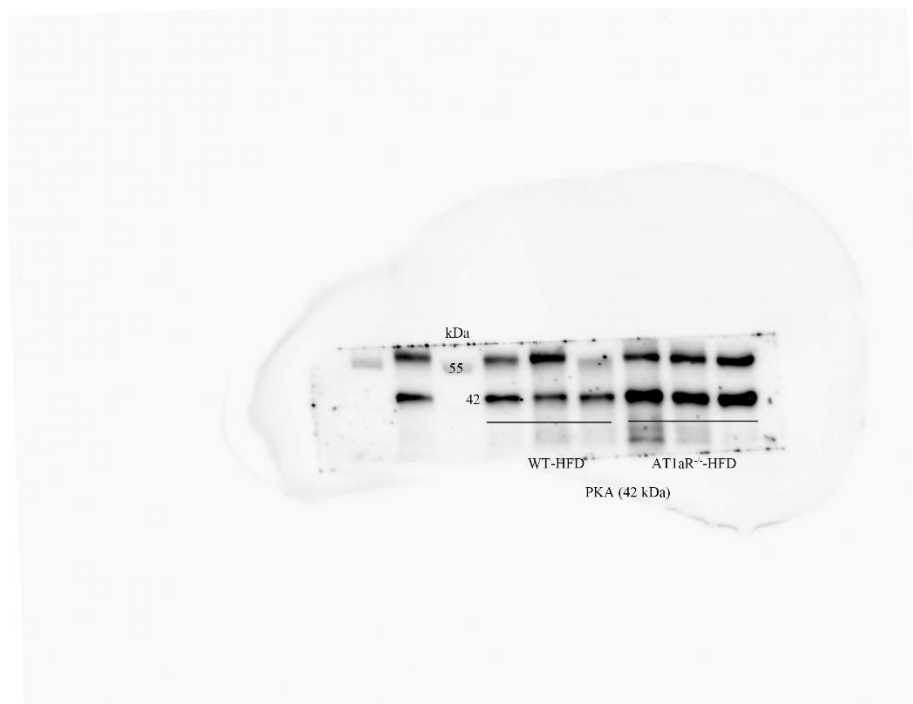

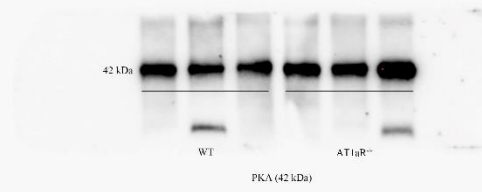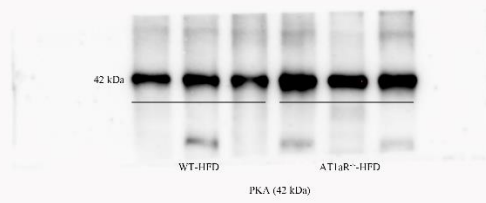

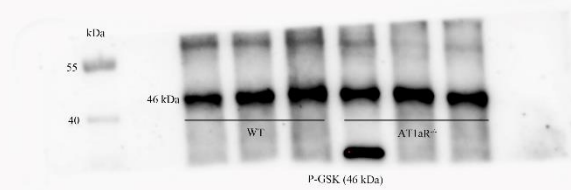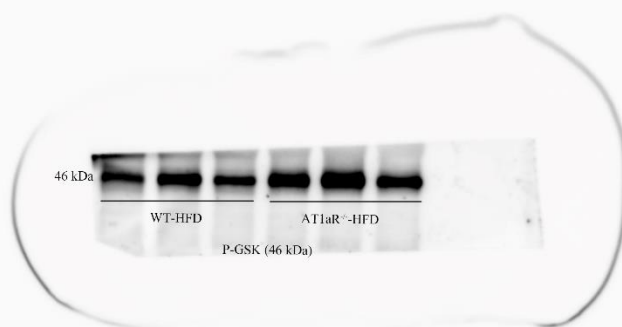

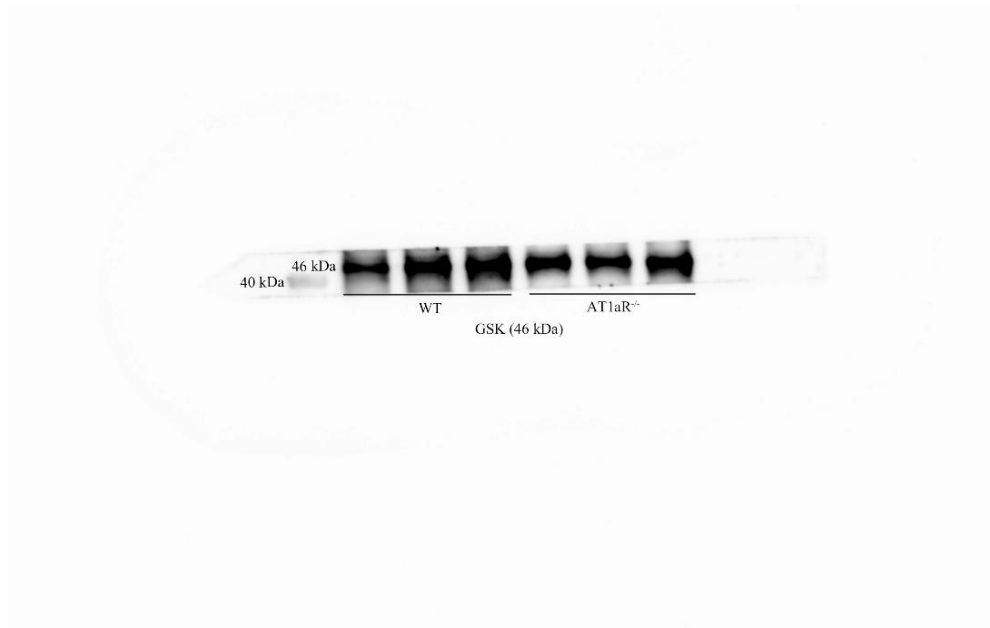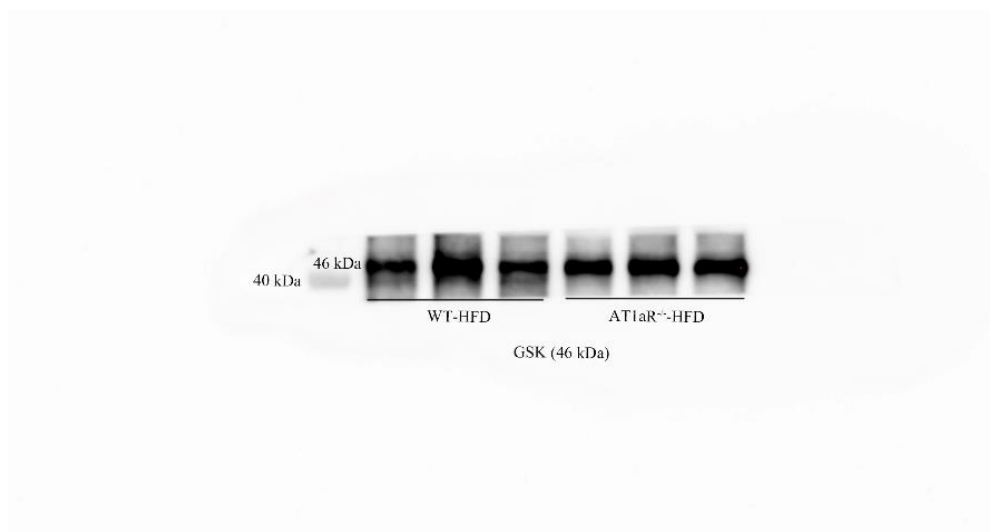

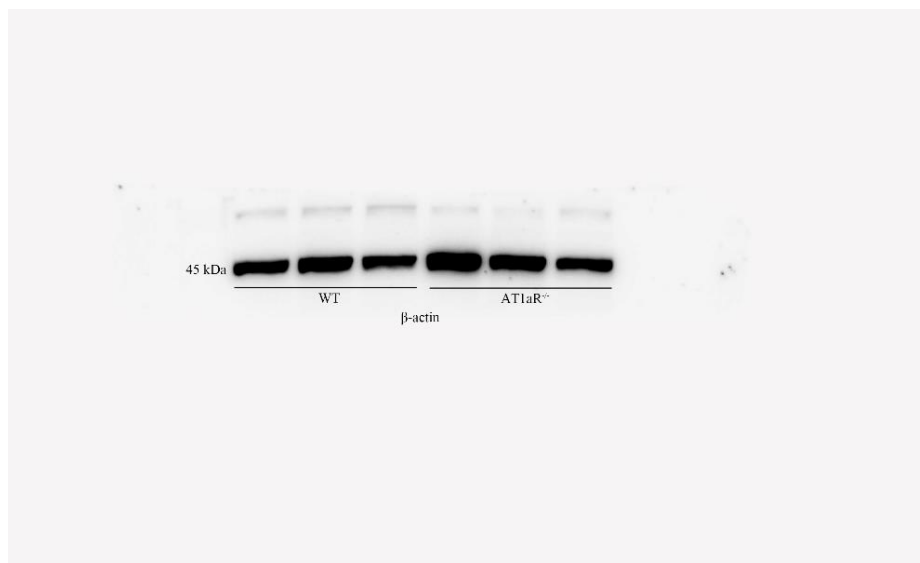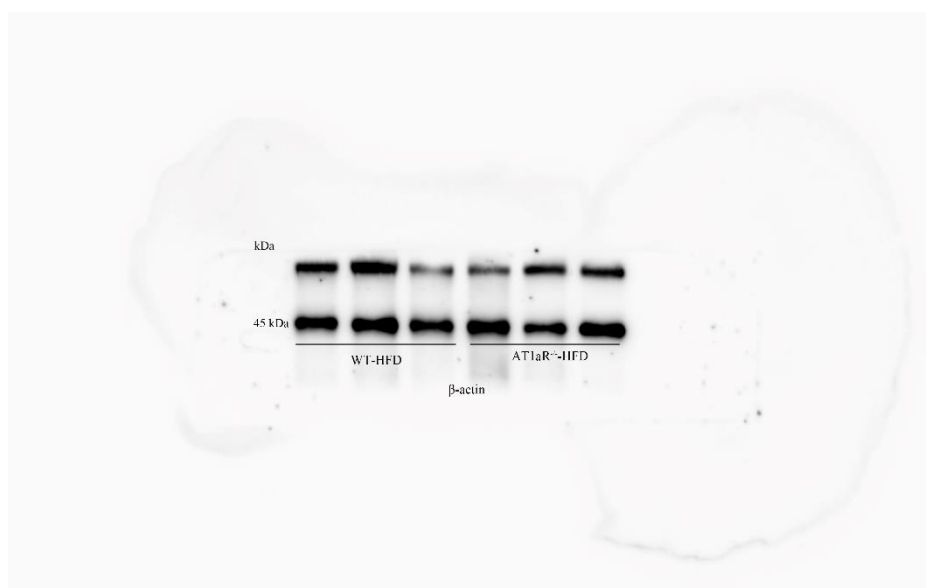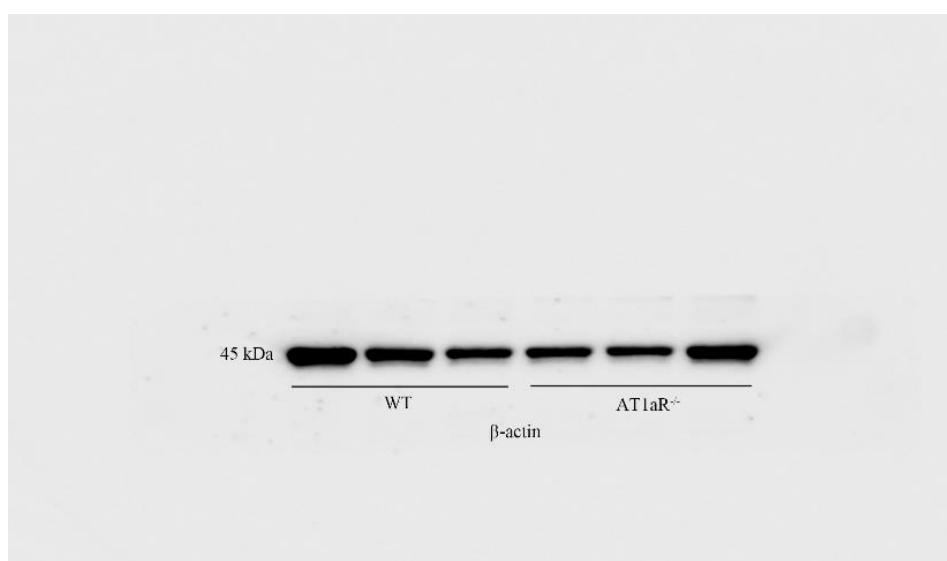

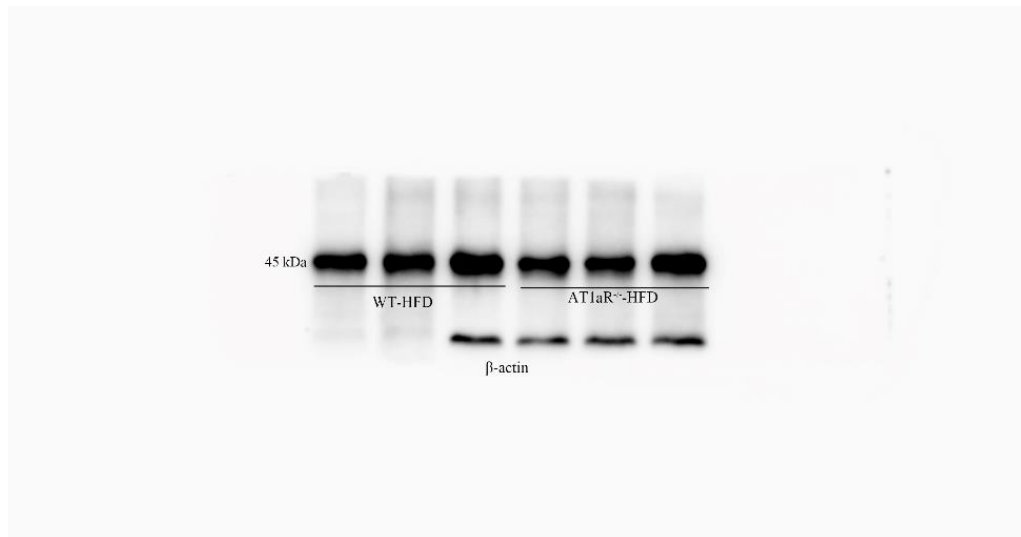

Raw images in Extended Fig 3

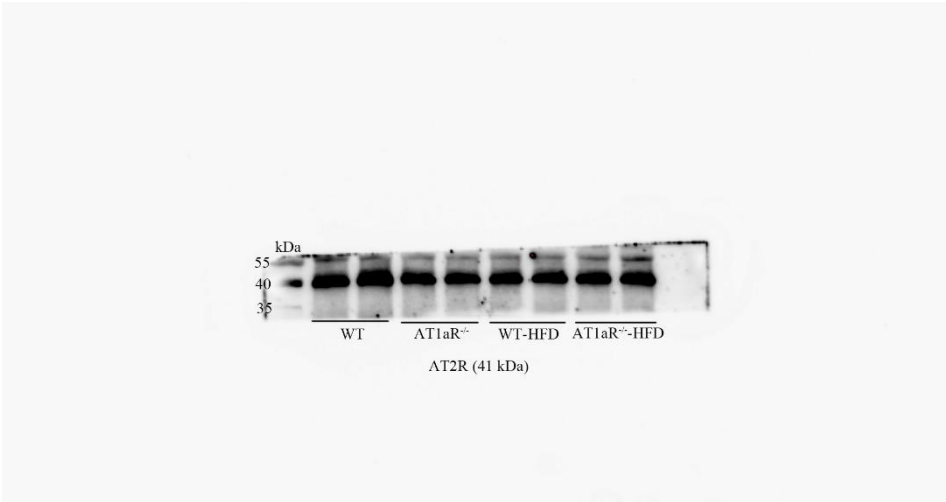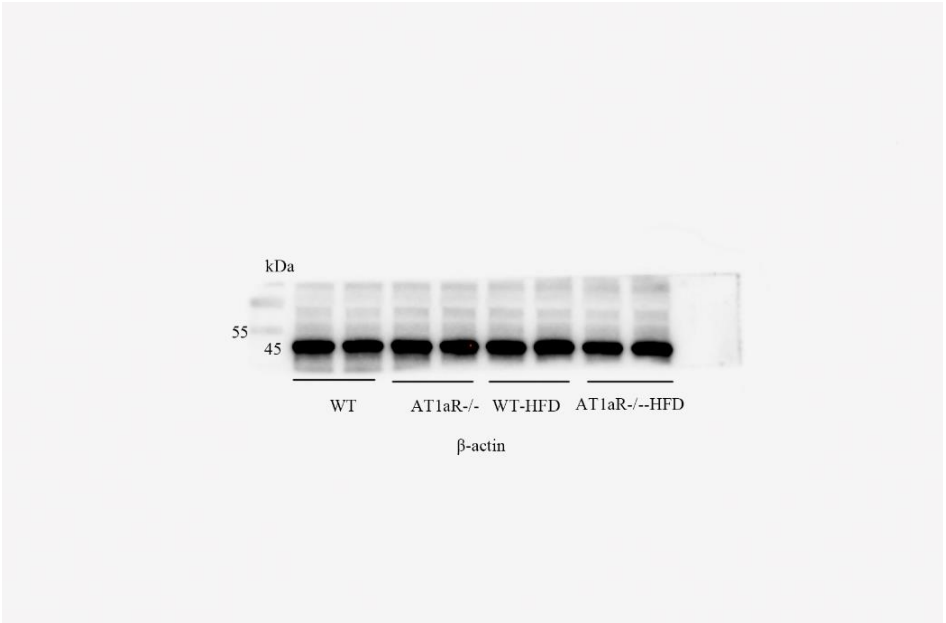

Supplement: S1 Raw images — (PDF) [file pone.0268580.s024.pdf]
